# PEX39 facilitates the peroxisomal import of PTS2-containing proteins

**DOI:** 10.1038/s41556-025-01711-z

**Published:** 2025-07-30

**Authors:** Walter W. Chen, Tony A. Rodrigues, Daniel Wendscheck, Ana G. Pedrosa, Chendong Yang, Tânia Francisco, Till Möcklinghoff, Alexandros Zografakis, Bernardo Nunes-Silva, Reut E. Avraham, Ana R. Silva, Maria J. Ferreira, Hirak Das, Janet Koster, Simone Neuwirth, Julian Bender, Silke Oeljeklaus, Varun Sondhi, Christos Gatsogiannis, Maya Schuldiner, Einat Zalckvar, Kay Hofmann, Hans R. Waterham, Ralph J. DeBerardinis, Jorge E. Azevedo, Bettina Warscheid

**Affiliations:** 1 Division of Neonatal-Perinatal Medicine, Department of Pediatrics, University of Texas Southwestern Medical Center, Dallas, TX USA; 2 Children’s Medical Center Research Institute, University of Texas Southwestern Medical Center, Dallas, TX USA; 3 Instituto de Investigação e Inovação em Saúde (i3S), Universidade do Porto, Porto, Portugal; 4 Instituto de Biologia Molecular e Celular, Universidade do Porto, Porto, Portugal; 5 Instituto de Ciências Biomédicas Abel Salazar, Universidade do Porto, Porto, Portugal; 6 Biochemistry II, Theodor-Boveri-Institute, Biocenter and Faculty of Chemistry and Pharmacy, University of Würzburg, Würzburg, Germany; 7 Biochemistry and Functional Proteomics, Institute of Biology II, Faculty of Biology, University of Freiburg, Freiburg, Germany; 8 Department of Molecular Genetics, Weizmann Institute of Science, Rehovot, Israel; 9 Laboratory Genetic Metabolic Diseases, Amsterdam UMC—location AMC, University of Amsterdam, Amsterdam, the Netherlands; 10 Institute of Medical Physics and Biophysics, University of Münster, Münster, Germany; 11 Center for Soft Nanoscience, University of Münster, Münster, Germany; 12 Division of Pulmonary and Critical Care Medicine, Department of Internal Medicine, University of Texas Southwestern Medical Center, Dallas, TX USA; 13 The Mina and Everard Goodman Faculty of Life Sciences, Bar-Ilan University, Ramat Gan, Israel; 14 Institute for Genetics, University of Cologne, Cologne, Germany; 15 Amsterdam Gastroenterology Endocrinology Metabolism, Amsterdam, the Netherlands; 16 Eugene McDermott Center for Human Growth and Development, University of Texas Southwestern Medical Center, Dallas, TX USA; 17 Howard Hughes Medical Institute, University of Texas Southwestern Medical Center, Dallas, TX USA; 18 Present Address: German Cancer Research Center, Systems Biology of Signal Transduction, Heidelberg, Germany

**Keywords:** Peroxisomes, Protein translocation, Protein-protein interaction networks, Proteins

## Abstract

Peroxisomes are metabolic organelles essential for human health. Defects in peroxisomal biogenesis proteins (also known as peroxins (PEXs)) cause devastating disease. PEX7 binds proteins containing a type 2 peroxisomal targeting signal (PTS2) to enable their import from the cytosol into peroxisomes, although many aspects of this process remain enigmatic. Utilizing in vitro assays, yeast and human cells, we show that PEX39, a previously uncharacterized protein, is a cytosolic peroxin that facilitates the import of PTS2-containing proteins by binding PEX7 and stabilizing its interaction with cargo proteins containing a PTS2. PEX39 and PEX13, a peroxisomal membrane translocon protein, both possess an (R/K)PWE motif necessary for PEX7 binding. Handover of PEX7 from PEX39 to PEX13 via these motifs provides a new paradigm for peroxisomal protein import and biogenesis. Collectively, this work reveals how PEX39 and (R/K)PWE motifs facilitate the import of PTS2-containing proteins and advances our understanding of peroxisomal disease.

## Abstract (web summary)

Chen et al. show that PEX39 cooperates with PEX7 in the peroxisomal import of proteins containing a PTS2 site and uncover an (R/K)PWE motif in PEX39 and PEX13 that binds to PEX7 and facilitates the import of PTS2-containing proteins.

## Main

Present in nearly all eukaryotes, peroxisomes are organelles that house important biochemical processes, such as fatty acid oxidation^1^. Peroxins (PEXs) are proteins essential for peroxisomal biogenesis, with multiple peroxins facilitating the import of enzymes from the cytosol into the peroxisomal matrix (that is, lumen)^2^. Defects in peroxins cause devastating human diseases, such as Zellweger spectrum disorders^3^.

The import of peroxisomal enzymes occurs via a remarkable mechanism through which even folded proteins and large protein complexes can reach the organellar matrix^4–6^. Peroxisomal enzymes harbour a type 1 peroxisomal targeting signal (PTS1; that is, a carboxy (C)-terminal tripeptide SKL sequence or a variant of it) or, less commonly, a PTS2 (that is, an amino (N)-terminal R–(L/V/I/Q)–X–X–(L/V/I/H)–(L/S/G/A)–X–(H/Q)–(L/A) sequence, where X can be any amino acid)^7,8^. Although few in number, PTS2-containing proteins serve crucial roles in cellular metabolism, with defects in the PTS2-containing proteins phytanoyl-CoA 2-hydroxylase (PHYH; required for fatty acid α-oxidation) and alkyl-DHAP synthase (AGPS; required for myelin synthesis) causing human diseases^9,10^. In the cytosol, the receptors PEX5 and PEX7 bind cargo through their PTS1 and PTS2, respectively, to initiate the import cycle. Notably, cargo-loaded PEX7 also requires the binding of a co-receptor (for example, Pex18/Pex21 in *Saccharomyces cerevisiae* and the long isoform of PEX5 in humans) to engage the downstream import machinery^7,8^. Cargo-loaded PEX5 or PEX7–co-receptor complexes are then recruited to the peroxisomal docking/translocation module containing the membrane proteins PEX13 and PEX14 (refs. ^11–19^), and in yeast Pex17 (refs. ^20–22^). Notably, it has been proposed that PEX13 forms the conduit through which peroxisomal matrix proteins are translocated^23,24^. However, our understanding of various aspects of peroxisomal protein import remains incomplete. In this Article, we demonstrate that the previously uncharacterized *S. cerevisiae* (yeast) protein Yjr012c and human protein C6ORF226 are orthologues of a peroxin we have named PEX39, which facilitates the import of PTS2-containing proteins.

## Results

### PEX39 interacts with members of the import pathway of PTS2-containing proteins

**Fig. 1:**
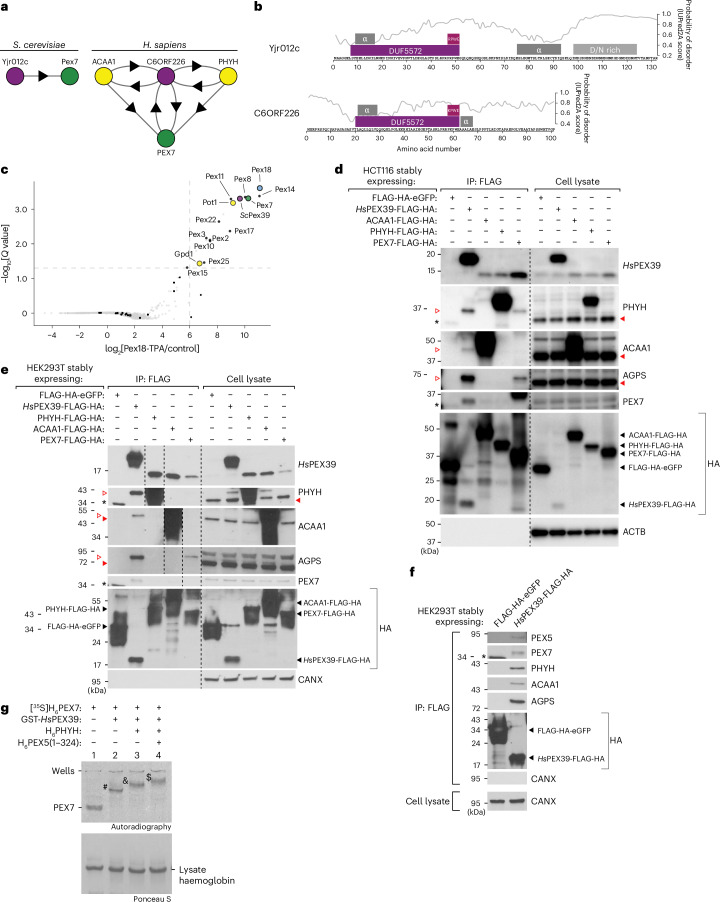
PEX39 interacts with members of the import pathway of PTS2-containing proteins. **a**, Depiction of protein–protein interactions for Yjr012c and C6ORF226, per BioGRID^25^ and BioPlex^26,27^, respectively. The circles represent proteins and the arrows point from purified proteins to interactors. For Yjr012c, only interactions found in at least two independent studies were considered. For C6ORF226, the combined interactome from HCT116 and HEK293T cells is shown. **b**, Domain analysis and alignment of Yjr012c and C6ORF226. Probabilities of disorder were determined using IUPred2A^56^. **c**, Quantitative proteomic analysis of Pex18 complexes that were affinity purified from soluble fractions of wild-type and Pex18-TPA-expressing yeast grown in oleic acid medium (*n* = 3). The enrichment of proteins in Pex18 complexes and *Q* values were determined using the rank-sum method^57^. Peroxins and PTS2-containing proteins are labelled and/or marked by black dots. The dashed lines indicate a *Q*-value threshold of 0.05 and a fold-enrichment of 64. **d**, Immunoblots of anti-FLAG immunoprecipitates prepared from HCT116 cells expressing the indicated proteins. HA denotes the detection of HA-tagged proteins. The dashed lines indicate where different lanes of the same membrane were brought together. For the PHYH, ACAA1 and AGPS blots, solid and open red arrowheads indicate mature and precursor forms of these proteins, respectively. An asterisk indicates a non-specific band. The control protein β-actin (ACTB) should be absent from immunoprecipitates. **e**, Immunoblots of anti-FLAG immunoprecipitates prepared from HEK293T cells expressing the indicated proteins. The control protein calnexin (CANX) should be absent from immunoprecipitates. The annotation of the immunoblots is otherwise the same as for **d**. **f**, Immunoblots of anti-FLAG immunoprecipitates prepared from HEK293T cells expressing FLAG-HA-eGFP or *Hs*PEX39-FLAG-HA. An asterisk indicates a non-specific band. **g**, Native PAGE and autoradiography of [^35^S]H_6_PEX7 pre-incubated with the recombinant proteins GST-*Hs*PEX39, H_6_PHYH and H_6_PEX5(1–324), as indicated. The in-gel positions of PEX7 alone, lysate haemoglobin and the complexes PEX7–*Hs*PEX39 (hashtag), PEX7–PHYH–*Hs*PEX39 (ampersand) and PEX7–PEX5–PHYH–*Hs*PEX39 (dollar sign) are indicated. The autoradiograph and corresponding Ponceau S-stained membrane are shown. α, α-helix; IP, immunoprecipitate. Source data

To better understand the import of PTS2-containing proteins, we examined PEX7 in the interactome databases BioGRID^25^ and BioPlex^26,27^. Our analysis revealed that two proteins of unknown function—*S. cerevisiae* Yjr012c and human C6ORF226—interact with PEX7 orthologues despite lacking a PTS2. The C6ORF226 interactome data also contain PHYH and peroxisomal 3-ketoacyl-CoA thiolase (ACAA1), two PTS2-containing proteins involved in fatty acid oxidation (Fig. 1a)^28^.

**Extended Data Fig. 1:**
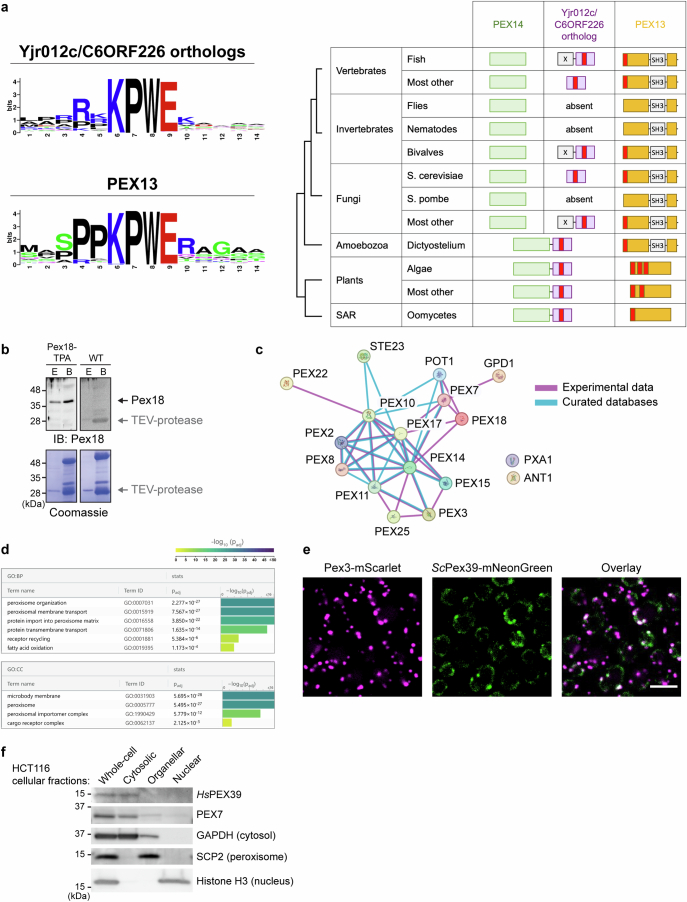
PEX39 and [R/K]PWE motif conservation, Pex18 enrichment, Pex18 interactome analysis, and PEX39 subcellular localization. **a**, Examination of [R/K]PWE motifs and Yjr012c/C6ORF226 orthologs across eukaryotes. Left: Sequence logos for the regions containing [R/K]PWE motifs in the indicated proteins. At each position, stack height indicates sequence conservation, while symbol height indicates the relative frequency of each residue. Right: Domain architecture of the indicated proteins across major eukaryotic taxa. Colored boxes indicate regions typical for PEX14 (green), Yjr012c/C6ORF226 orthologs (purple), and PEX13 (orange). Red boxes denote [R/K]PWE motifs. Grey boxes indicate additional domains only found in some taxa. In most fungi, invertebrates and fishes, Yjr012c/C6ORF226 orthologs possess an additional region of unknown function (“X”) that is distinct from the DUF5572. SAR, *Stramenopiles-Alveolata-Rhizaria*. **b**, Immunoblots of eluates (E) and bead (B) samples of the soluble fractions of wild-type (WT) yeast cells and cells expressing Pex18-TPA. TEV-protease was added to beads to cleave the TPA tag and elute Pex18 complexes. **c**, STRING network analysis representing network of proteins (nodes) from Pex18-TPA complexes dataset with a Q-value of < 0.05. The network is based on data received from curated databases and experiments and was filtered by a “minimum required interaction score” of 0.4 (medium confidence). Interactions without experimental/biochemical data or those that are based on putative homologues in other organisms were not considered. **d**, Gene Ontology (GO) enrichment analysis of proteins with a Q-value of < 0.05 for the domains “biological process” (BP) and “cellular component” (CC). Listed are selected terms with an adjusted P value (P_adj_) of < 0.05. GO term enrichment analysis was performed using the g:Profiler application (https://biit.cs.ut.ee/gprofiler/gost)^83^ and adjusted P values determined via the Benjamini-Hochberg method. **e**, Fluorescence microscopy of *Sc*Pex39-mNeonGreen in yeast grown in medium with oleic acid as the primary energy source, a condition that stimulates peroxisomal biogenesis^35^. Peroxisomes were visualized with Pex3-mScarlet. Scale bar: 5 μm. **f**, Immunoblots of cellular fractions from wild-type HCT116 cells. In parentheses are the subcellular compartments that GAPDH, SCP2, and Histone H3 are markers for. SCP2 represents the ~15 kDa protein that results from transcription of a downstream promoter for the *SCP2* gene and has been used previously as a marker of peroxisomes^111,112^. Source data

A comparison of the C6ORF226 and Yjr012c sequences revealed notable similarities, such as the presence of a domain of unknown function (DUF5572) and an (R/K)PWE motif at the DUF5572 C terminus, indicating that they are orthologues (Fig. 1b). An examination of numerous organisms revealed that the most conserved feature of Yjr012c/C6ORF226 orthologues is the (R/K)PWE motif (Extended Data Fig. 1a, left). Despite lacking a DUF5572 domain, PEX13 also possesses a conserved KPWE motif at its N terminus, as observed previously^29^. Notably, Yjr012c/C6ORF226 orthologues occur in all eukaryotic kingdoms, suggesting a similar evolutionary age to known peroxins (Extended Data Fig. 1a, right). Interestingly, orthologues are absent from *Caenorhabditis elegans* and *Drosophila melanogaster*, which lack the PTS2-containing protein pathway^30,31^, as well as from *Schizosaccharomyces pombe*, which does not import cargo proteins containing a PTS2 (ref. ^32^). Remarkably, outside of opisthokonts (for example, fungi and animals), Yjr012c-/C6ORF226-related sequences are fused to PEX14. Collectively, these data suggest that Yjr012c/C6ORF226 are peroxins involved in protein import. Hereafter, we refer to *Homo sapiens* C6ORF226 as *Hs*PEX39 and *S. cerevisiae* Yjr012c as *Sc*Pex39.

Next, we explored the interactions reported for *Sc*Pex39 and *Hs*PEX39 (Fig. 1a). In *S. cerevisiae*, quantitative affinity purification–mass spectrometry experiments using Pex18 fused C terminally with the tobacco etch virus (TEV) protease cleavage site and protein A (TPA) tag (Pex18-TPA) as bait revealed *Sc*Pex39 as a specific interactor, along with Pex7, other peroxins and the PTS2-containing protein ACAA1 (Pot1) (Fig. 1c, Extended Data Fig. 1b–d and Supplementary Table 2). In the human cell lines HCT116 (Fig. 1d) and HEK293T (Fig. 1e), PHYH, ACAA1 and PEX7 tagged with FLAG-HA co-immunoprecipitated endogenous *Hs*PEX39, whereas an enhanced green fluorescent protein (eGFP) control did not. *Hs*PEX39-FLAG-HA also co-immunoprecipitated endogenous PEX7 and the three PTS2-containing proteins PHYH, ACAA1 and AGPS. In addition, PHYH, ACAA1 and AGPS co-immunoprecipitating with *Hs*PEX39-FLAG-HA or PEX7-FLAG-HA migrated at the expected molecular weights of their precursor forms, consistent with PEX7 only being able to bind precursor PTS2-containing proteins due to removal of the PTS2 when the protein is converted to the mature form in the peroxisomal lumen; of note, this maturation process for PTS2-containing proteins does not occur in yeast^8^. In agreement with the interaction between Pex18 and *Sc*Pex39 in yeast (Fig. 1c), *Hs*PEX39-FLAG-HA also co-immunoprecipitated PEX5 from human cell lysates (Fig. 1f). Consistent with PEX39 interacting with PEX7, PEX5/Pex18 and cargo proteins containing a PTS2, fluorescence microscopy revealed that the fusion protein *Sc*Pex39-mNeonGreen localized to both the cytosol and peroxisomes (Extended Data Fig. 1e), whereas cellular fractionation revealed that endogenous *Hs*PEX39 is cytosolic (Extended Data Fig. 1f).

**Extended Data Fig. 2:**
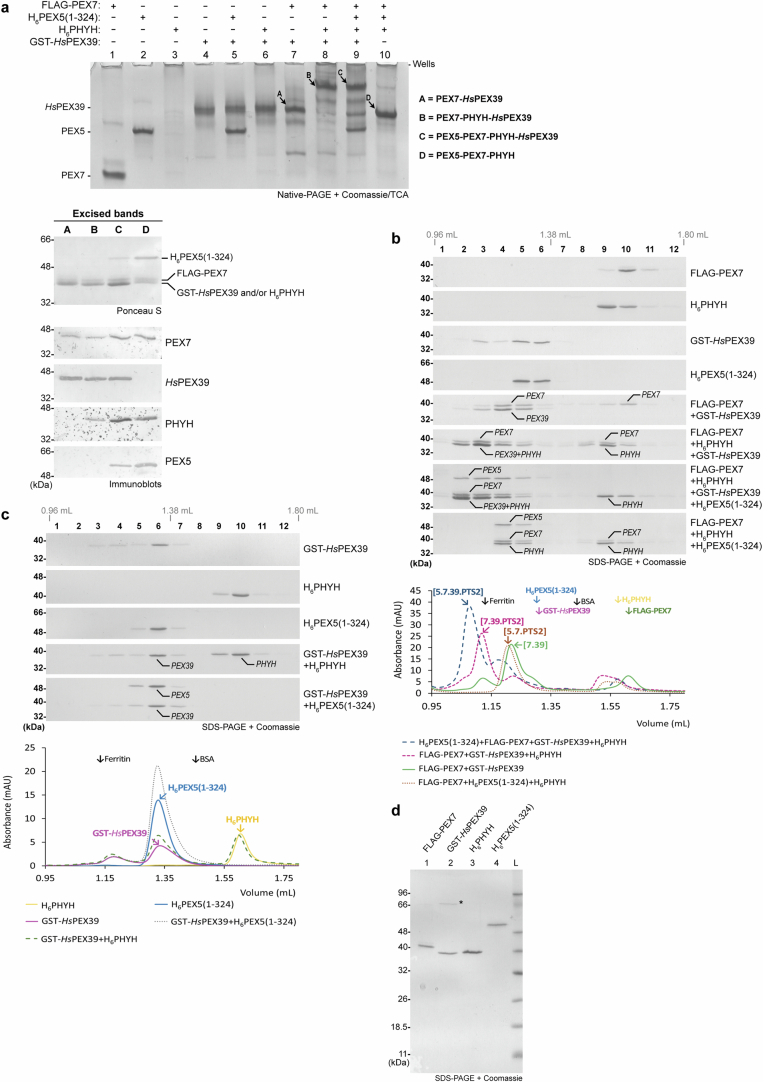
Investigation of protein complex formation involving *Hs*PEX39 *in vitro.* **a**, Determination of protein composition of complexes identified by native-PAGE assays. Top: The indicated recombinant proteins, alone or mixed together, were analyzed by native-PAGE and Coomassie/TCA staining. In-gel positions of PEX7 alone (PEX7), PEX5 alone (PEX5), *Hs*PEX39 alone (*Hs*PEX39), and protein complexes A-D are indicated. Bottom: Bands for complexes A-D in the native gel (top) were excised from a Ponceau S-stained membrane, eluted with Laemmli sample buffer, and analyzed by SDS-PAGE and immunoblotting to verify the protein composition of the complexes. **b**, Recombinant FLAG-PEX7, GST-*Hs*PEX39, H_6_PHYH, and H_6_PEX5(1-324) were analyzed alone or in the indicated mixtures using size-exclusion chromatography (SEC). Collected fractions were analyzed by SDS-PAGE and Coomassie staining (top). The corresponding area of the chromatogram is also shown (bottom). The elution volumes of the protein complexes, individual recombinant proteins, and molecular weight standards (Ferritin and BSA) are indicated. **c**, Recombinant GST-*Hs*PEX39, H_6_PHYH, and H_6_PEX5(1-324) were analyzed alone or in the indicated mixtures by SEC. Collected fractions were analyzed by SDS-PAGE and Coomassie staining (top). The corresponding area of the chromatogram is also shown (bottom). The elution volumes of the species of interest and molecular weight standards (Ferritin, BSA) are indicated. **d**, The purity of FLAG-PEX7, GST-*Hs*PEX39, H_6_PHYH, and H_6_PEX5(1-324) was assessed by SDS-PAGE and Coomassie staining. The asterisk indicates an *Escherichia coli* contaminant that co-purifies with recombinant *Hs*PEX39. L, protein ladder. Source data

To further explore the observed interactions, we utilized native polyacrylamide gel electrophoresis (native PAGE) to monitor complex formation between radiolabelled human [^35^S]H_6_PEX7 and different recombinant proteins, as reflected by changes in the migration of [^35^S]H_6_PEX7 (that is, gel shifts)^33^. Gel shifts revealed that PEX7–*Hs*PEX39 (hashtag in Fig. 1g), PEX7–PHYH–*Hs*PEX39 (ampersand in Fig. 1g) and PEX7–PEX5–PHYH–*Hs*PEX39 (dollar sign in Fig. 1g) complexes are formed. Utilizing recombinant human FLAG-tagged PEX7 instead of [^35^S]H_6_PEX7 in our native PAGE assays, we observed the same complexes, as well as the previously reported PEX5–PEX7–PHYH complex^33^ (Extended Data Fig. 2a, top). Immunoblotting confirmed the composition of all of these complexes (Extended Data Fig. 2a, bottom). We also used size exclusion chromatography (SEC) to demonstrate the formation of these complexes (Extended Data Fig. 2b). *Hs*PEX39 could not complex with either PEX5 or PHYH alone, per native PAGE (Extended Data Fig. 2a) and SEC (Extended Data Fig. 2c). Extended Data Fig. 2d provides the purities of the recombinant proteins.

### Loss of PEX39 impairs the import of PTS2-containing proteins

**Fig. 2:**
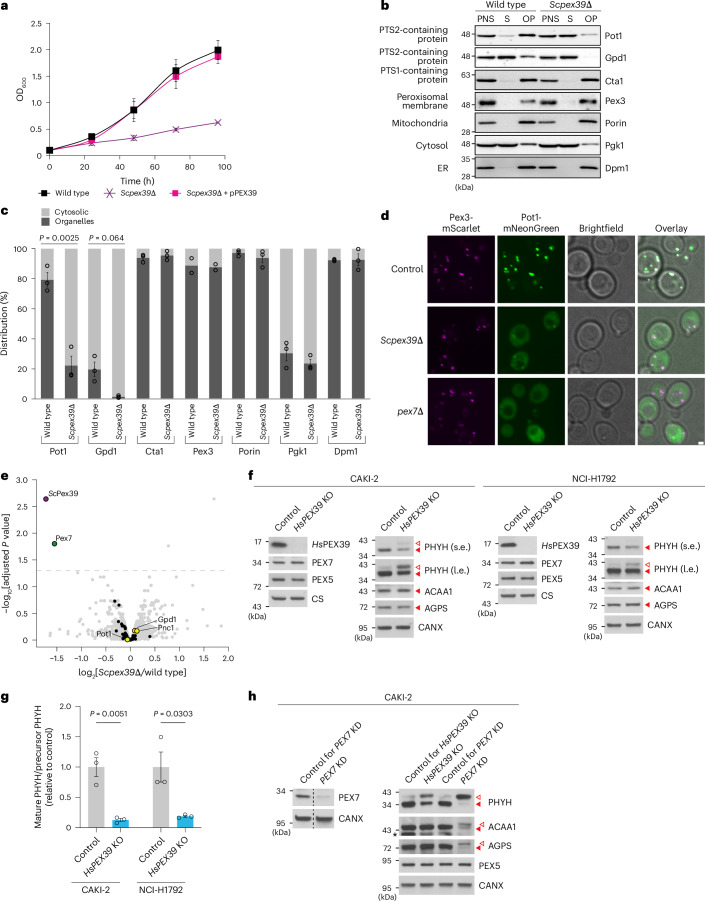
Loss of PEX39 impairs the import of PTS2-containing proteins. **a**, Growth of the indicated yeast strains in oleic acid medium. pPEX39 is a plasmid containing *Scpex39* under the control of its endogenous promoter. The data represent means ± s.d. (*n* = 4). **b**, Immunoblots of cellular fractions from wild-type and *Scpex39*Δ yeast. A post-nuclear supernatant (PNS) was prepared from cells grown in oleic acid medium and further separated into a cytosolic supernatant (S) and organellar pellet (OP). **c**, Quantification of the subcellular distribution of proteins using the band intensities of the immunoblots shown in **b** and Extended Data Fig. 3c. For each protein, the summed intensities of the cytosolic supernatant and organelles were set to 100%. The data represent means ± s.e.m. (*n* = 2 for Pex3 and *n* = 3 for the rest). Statistical significance was determined by unpaired, two-tailed *t*-test. **d**, Fluorescence microscopy of Pot1-mNeonGreen in control, *Scpex39*Δ and *pex7*Δ yeast grown in oleic acid medium. Peroxisomes were visualized with Pex3-mScarlet. Scale bar, 1 μm. **e**, Quantitative proteomic analysis of wild-type and *Scpex39*Δ yeast (*n* = 4). Proteins quantified in at least three biological replicates are shown, except for *Sc*Pex39 (quantified in one replicate). PTS2-containing proteins and additional peroxisomal proteins are indicated by yellow and black circles, respectively. Multiple-testing-adjusted *P* values were determined using the limma approach (moderated two-tailed *t*-test) and Benjamini–Hochberg method. The dashed line indicates an adjusted *P* value threshold of 0.05. **f**, Immunoblot analysis of control and *HsPEX39*-KO CAKI-2 and NCI-H1792 cells. Precursor ACAA1 and AGPS were undetectable. CANX and citrate synthase (CS) were used as loading controls. **g**, Quantification of mature and precursor PHYH in *HsPEX39*-KO cells using the band intensities of immunoblots prepared per **f**. Mature/precursor ratios were divided by the mean value of the corresponding control. The data represent means ± s.e.m. (*n* = 3). Statistical significance was determined by unpaired, two-tailed *t*-test. **h**, Immunoblot analysis of *PEX7*-KD (*PEX7*-knockdown) and *HsPEX39*-KO CAKI-2 cells. The dashed lines indicate where different lanes of the same membrane were brought together. An asterisk indicates a non-specific band. In **f** and **h**, the solid and open red arrowheads indicate mature and precursor forms, respectively. ER, endoplasmic reticulum; l.e., long exposure; s.e., short exposure. Source data

**Extended Data Fig. 3:**
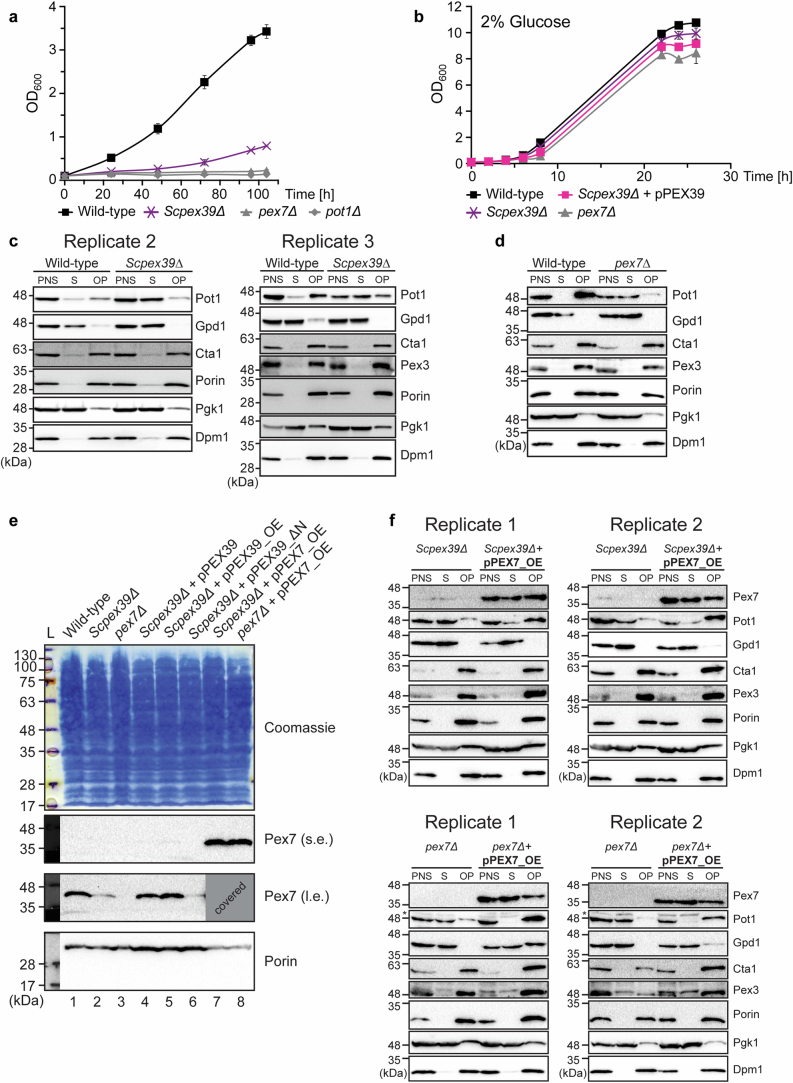
Characterization of PEX39 loss in yeast cells. **a**, Growth of the indicated yeast strains in oleic acid medium. Data are mean ± standard deviation (SD) (n = 4). **b**, Growth of the indicated yeast strains in glucose medium. Data are mean ± SD (n = 3). **c**, Immunoblots of the two additional biological replicates used for quantification of the subcellular distribution of proteins in Fig. 2c. Experiments performed as described in Fig. 2b using the indicated strains. **d**, Immunoblots of cellular fractions from wild-type and *pex7Δ* yeast. Experiment performed as described in Fig. 2b. **e**, Immunoblot analysis of Pex7 levels in the indicated yeast strains. Wild-type, *Scpex39Δ*, *pex7Δ* yeast and deletion strains transformed with plasmids containing *Scpex39* or *pex7* under the control of endogenous promotors or a strong TEF2 overexpression promotor (_OE) were cultured in oleic acid medium. pPEX39_ΔN, plasmid encoding for an N-terminally truncated variant of *Sc*Pex39 (amino acids 40-132) that retains the RPWE motif. Detection of endogenous Pex7 levels was accomplished by covering intense signals detected for overexpressed Pex7. Shorter and longer exposures denoted by s.e. and l.e., respectively. L, protein ladder. **f**, Immunoblots of cellular fractions from *Scpex39Δ* and *pex7Δ* yeast transformed with a plasmid containing *pex7* under the TEF2 promoter (pPEX7_OE). Experiments were performed as described in Fig. 2b. An asterisk indicates a non-specific band. Two biological replicates are shown. **c**,**d**,**f**, PNS, post-nuclear supernatant; S, cytosolic supernatant; OP, organellar pellet. Source data

For a protein to be considered a peroxin, it must participate in a process required for peroxisomal biogenesis, such as the import of peroxisomal proteins^32,34^. To explore the functional importance of PEX39 in peroxisomal biogenesis, we generated loss-of-function models in both yeast and human cells. We first assessed the growth of *Scpex39*-null (*Scpex39*Δ) and *pex7*Δ yeast in liquid media with glucose or oleic acid as the primary energy source. With oleic acid, peroxisomal biogenesis is stimulated and cellular proliferation requires β-oxidation of fatty acids, which occurs exclusively in peroxisomes in *S. cerevisiae*^35^. Because the PTS2-containing protein Pot1 is necessary for β-oxidation, defects in the import of PTS2-containing proteins impair the fitness of oleic acid-grown cells but not glucose-grown cells^36^. Consistent with this, *Scpex39*Δ and *pex7*Δ cells had reduced growth in oleic acid medium (Fig. 2a and Extended Data Fig. 3a) but similar fitness in glucose medium (Extended Data Fig. 3b). Importantly, the poor growth of *Scpex39*Δ cells could be rescued by introduction of the *Scpex39* gene under the control of its native promoter (Fig. 2a).

Cellular fractionation of *Scpex39*Δ yeast revealed a redistribution from the organellar pellet to the cytosol of Pot1 and, to a lesser extent, the PTS2-containing protein glycerol-3-phosphate dehydrogenase (Gpd1), which localizes to the cytosol, nucleus and peroxisomes^37^. In contrast, no changes were noted for the PTS1-containing protein Cta1 or the peroxisomal membrane protein Pex3 (Fig. 2b,c and Extended Data Fig. 3c). As expected, Pot1 was redistributed to the cytosol in *pex7*Δ yeast (Extended Data Fig. 3d). Fluorescence microscopy also revealed a redistribution of Pot1 fused to mNeonGreen from peroxisomes to the cytosol in *Scpex39*Δ yeast, albeit to a lesser degree than in *pex7*Δ yeast (Fig. 2d).

Interestingly, quantitative proteomics on *Scpex39*Δ yeast demonstrated that Pex7 was greatly decreased but PTS2-containing proteins and other peroxins, including Pex5 and Pex18, were not (Fig. 2e and Supplementary Table 3). This decrease in Pex7, which was confirmed by immunoblotting (Extended Data Fig. 3e, lanes 1, 2 and 4), probably contributes to the impaired import of PTS2-containing proteins seen with *Sc*Pex39 loss. Consistent with this, Pex7 overexpression in *Scpex39*Δ yeast facilitated the import of PTS2-containing proteins, but to a lesser extent than in *pex7*Δ cells, suggesting that *Sc*Pex39 facilitates import beyond just the modulation of Pex7 levels (Extended Data Fig. 3e (lanes 1, 7 and 8) and Extended Data Fig. 3f).

**Extended Data Fig. 4:**
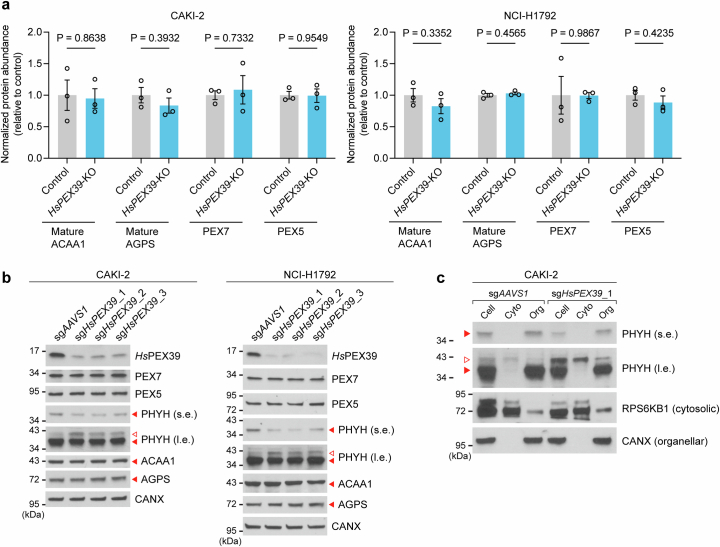
Characterization of PEX39 loss in human cells. **a**, Quantification of mature ACAA1, mature AGPS, PEX7, and PEX5 in *HsPEX39*-knockout cells using band intensities of immunoblots prepared per Fig. 2f. Loading-control intensities were used to normalize protein abundance across samples and the normalized abundances were then divided by the mean value of the corresponding control cells. The names of quantified proteins are shown on the bottom. Precursor forms of ACAA1 and AGPS were undetectable and thus not quantified. Data are mean ± standard error of the mean (SEM) (n = 3), and P values were calculated using unpaired, two-tailed t-tests. **b**, Immunoblot analysis of cells generated via lentiviral transduction of Cas9 and multiple independent sgRNAs against *HsPEX39* (sg*HsPEX39*_1-3) or a negative control sgRNA against the *AAVS1* gene (sg*AAVS1*) in the CAKI-2 and NCI-H1792 cell lines. Note that the residual *Hs*PEX39 signal in these cells is because not all cells in the population will have complete loss of the protein. Solid and open red arrowheads indicate mature and precursor forms, respectively. Precursor forms of ACAA1 and AGPS were undetectable. CANX is a loading control. Short and long exposures are denoted s.e. and l.e., respectively. **c**, Immunoblots of whole-cell (Cell), cytosolic (Cyto), and organellar (Org) fractions prepared from the CAKI-2 cells expressing sg*AAVS1* (negative control) or sg*HsPEX39*_1 described in **b**. Solid and open red arrowheads indicate mature and precursor forms, respectively. The cellular fractions that RPS6KB1 and CANX are markers for are indicated in parentheses. Source data

To explore the consequences of *Hs*PEX39 loss, we used CRISPR–Cas9 to generate *HsPEX39* knockouts (KOs) and matched controls in human CAKI-2 and NCI-H1792 cells (see Methods for rationale). Loss of *Hs*PEX39 did not affect PEX7 and PEX5 levels (Fig. 2f and Extended Data Fig. 4a). The lack of a decrease in PEX7 levels contrasts with what we observed in yeast. Importantly though, loss of *Hs*PEX39 increased precursor PHYH and reduced mature PHYH, consistent with a defect in the peroxisomal import of PHYH (Fig. 2f,g). However, no changes in ACAA1 or AGPS were seen (Fig. 2f and Extended Data Fig. 4a). Consistent with our observations in yeast (Fig. 2d), loss of *Hs*PEX39 impaired the import of PTS2-containing proteins to a lesser degree than loss of PEX7 (Fig. 2h). Partial depletion of *Hs*PEX39 via lentiviral CRISPR–Cas9 with additional independent single guide RNAs (sgRNAs) corroborated our findings (Extended Data Fig. 4b). Importantly, cellular fractionation revealed that *Hs*PEX39 loss led to an accumulation of precursor PHYH in the cytosolic fraction and a decrease of mature PHYH in the organellar fraction, thus demonstrating impaired import of PTS2-containing proteins (Extended Data Fig. 4c).

### Overexpression of PEX39 impairs the import of PTS2-containing proteins

**Fig. 3:**
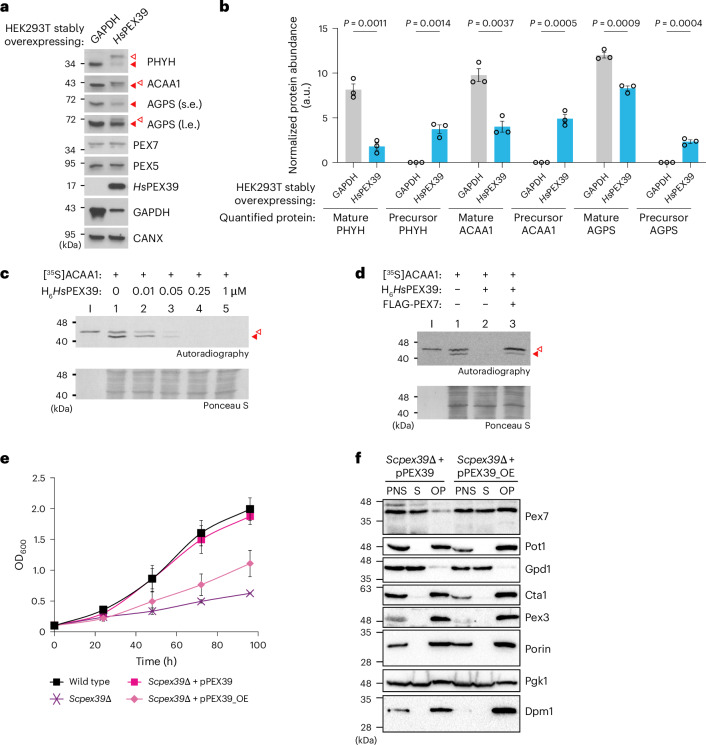
Overexpression of PEX39 impairs the import of PTS2-containing proteins. **a**, Immunoblot analysis of HEK293T cells overexpressing GAPDH (negative control) or *Hs*PEX39. The solid and open red arrowheads indicate mature and precursor forms, respectively. **b**, Quantification of changes in mature and precursor forms using the band intensities of immunoblots prepared per **a**. The precursor and mature form intensities were normalized by the intensities of the corresponding loading controls. The data represent means ± s.e.m. (*n* = 3). Statistical significance was determined by unpaired, two-tailed *t*-test. **c**,**d**, [^35^S]ACAA1 in vitro import assays in the presence of increasing concentrations of H_6_*Hs*PEX39 (**c**) or in the presence or absence of H_6_*Hs*PEX39 and FLAG-PEX7 (**d**). After incubation, the reactions were treated with trypsin and organelles were isolated by centrifugation and analysed by SDS-PAGE and autoradiography. Protection from trypsin and maturation of ACAA1 reflect import into peroxisomes. Precursor and mature ACAA1 are indicated by open and solid red arrowheads, respectively. I represents input (5% of the reticulocyte lysate containing the [^35^S]ACAA1 used in each reaction). **e**, Growth of the indicated yeast strains in oleic acid medium. pPEX39_OE represents a plasmid containing *Scpex39* under the control of a TEF2 promoter for overexpression. The data for the wild type, *Scpex39*Δ and *Scpex39*Δ + pPEX39 are the same as those shown in Fig. 2a. The data represent means ± s.d. (*n* = 4). **f**, Immunoblots of cellular fractions from yeast overexpressing *Sc*Pex39. The experiment was performed as described for Fig. 2b using *Scpex39*Δ cells transformed with a plasmid containing *Scpex39* under the control of the endogenous promoter (pPEX39) or an overexpressing TEF2 promoter (pPEX39_OE). Source data

**Extended Data Fig. 5:**
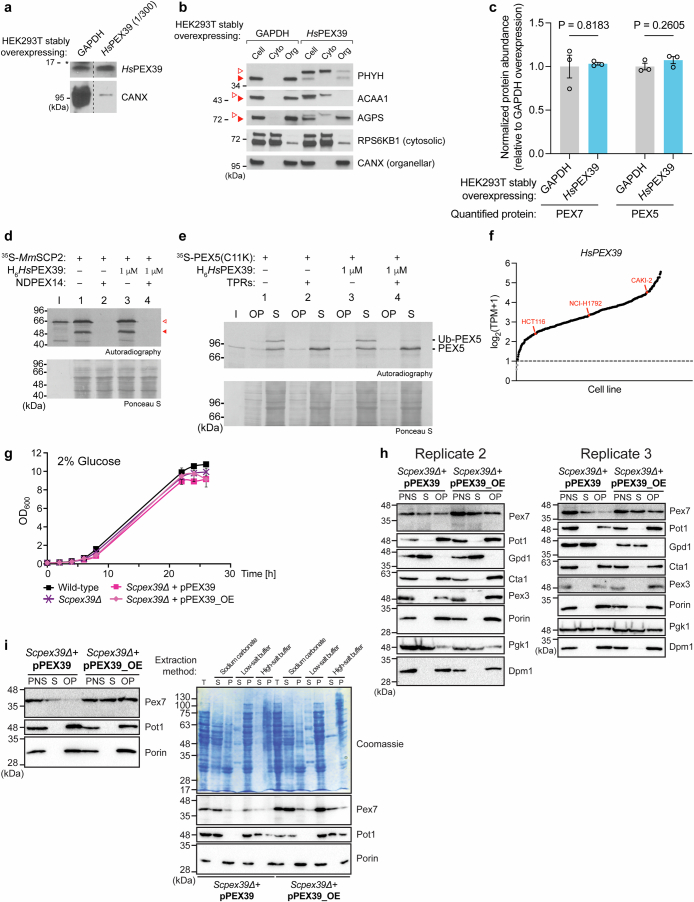
Investigation of the effects of excess PEX39. **a**, Immunoblot analysis of HEK293T cells overexpressing GAPDH or *Hs*PEX39 (1/300 dilution of cellular lysate). Asterisk indicates non-specific band. **b**, Immunoblots of whole-cell (Cell), cytosolic (Cyto), and organellar (Org) fractions from the indicated cells. Solid and open arrowheads indicate mature and precursor forms, respectively. **c**, PEX7 and PEX5 band intensities from immunoblots prepared per Fig. 3a were normalized to those of loading controls. Normalized protein abundances were then divided by the mean of GAPDH overexpressing cells. Data are mean ± SEM (n = 3). P values are calculated using unpaired, two-tailed t-tests. **d**, ^35^S-*Mm*SCP2 (*Mus musculus* SCP2) *in vitro* import assays in the presence of the indicated proteins. After incubation, reactions were treated with trypsin, and organelles were analyzed by SDS-PAGE and autoradiography. SCP2 is a ~ 60 kDa protein (open arrowhead) that is processed within the peroxisome to a smaller form^113^ (solid arrowhead). NDPEX14, *Hs*PEX14 residues 1-80 that inhibits protein import^38^. **e**, ^35^S-PEX5(C11K) *in vitro* import assays in the presence of the indicated proteins. After incubation, organellar pellet (OP) and cytosolic supernatant (S) fractions were analyzed by SDS-PAGE and autoradiography. Unmodified ^35^S-PEX5(C11K) (“PEX5”) and monoubiquitinated ^35^S-PEX5(C11K) (“Ub-PEX5”) are indicated. Monoubiquitinated ^35^S-PEX5(C11K) in the supernatant reflects successful PEX5 processing by peroxisomes and import of PTS1-containing proteins. TPR, PEX5 variant that inhibits PEX5(C11K) processing^114^. **d**,**e**, **i**, input (5% of the reticulocyte lysate containing the ^35^S-labeled protein used in each reaction). **f**, *HsPEX39* RNA-seq data (TPM) from the DepMap Portal (https://depmap.org/portal/) for 369 cell lines for which proteomics was performed and *Hs*PEX39 was undetectable^40^. Cell lines used in our study are red. The dashed line indicates the cutoff (TPM = 1) for the *HsPEX39* gene to be considered expressed; cell lines that do not meet that cutoff are grey. **g**, Growth of the indicated yeast strains in glucose medium. Data for wild-type, *Scpex39Δ*, and *Scpex39Δ* + pPEX39 are the same as shown in Extended Data Fig. 3b. Data are mean ± SD (n = 3). **h**, Biological replicates for the experiment in Fig. 3f. **i**, Characterization of organelle-associated Pex7 and Pot1 in yeast overexpressing *Sc*Pex39. Left: immunoblots of cellular fractions of yeast overexpressing *Sc*Pex39 as described for Fig. 3f. Right: immunoblot analysis of salt-extraction of proteins from organellar pellets isolated as described for Fig. 3f. T, total; S, supernatant fraction; P, pellet fraction. Source data

Having assessed the consequences of depleting PEX39, we next investigated the effects of increasing it. Surprisingly, overexpression of *Hs*PEX39 in wild-type HEK293T cells (Extended Data Fig. 5a) increased the precursor forms and decreased the mature forms of all three PTS2-containing proteins (Fig. 3a,b), with cellular fractionation revealing an accumulation of the precursor forms in the cytosolic fraction and a decrease of mature forms in the organellar fraction (Extended Data Fig. 5b), thus demonstrating impaired import for all three PTS2-containing proteins. Similar to loss of *Hs*PEX39, overexpression did not alter PEX5 or PEX7 levels (Extended Data Fig. 5c).

To investigate further, we used an established in vitro peroxisomal protein import system, in which a radiolabelled reporter protein (for example, a matrix protein containing a PTS1 or PTS2, or the co-receptor PEX5) is incubated with post-nuclear supernatant (PNS) containing cytosol and peroxisomes^38^. Specific import of radiolabelled matrix proteins is assessed by the acquisition of an organelle-associated and protease-resistant status. For PTS2-containing proteins, the appearance of radiolabelled mature forms also reflects successful import, as proteolytic processing occurs only in the peroxisomal matrix^39^.

Consistent with our observations in human cells, exogenous addition of recombinant *Hs*PEX39 strongly inhibited in vitro import of [^35^S]ACAA1 at low nanomolar concentrations (Fig. 3c). Interestingly, this inhibition could be ameliorated by the addition of excess PEX7 (Fig. 3d). In contrast with the import of PTS2-containing proteins, recombinant *Hs*PEX39 had no effect on the import of PTS1-containing proteins in vitro, as assessed by import of the PTS1-containing protein SCP2 (Extended Data Fig. 5d) and monoubiquitination of radiolabelled PEX5 at the peroxisome and its extraction back into the cytosol (Extended Data Fig. 5e). Given that excess *Hs*PEX39 can potently inhibit the import of PTS2-containing proteins, we wondered whether cells may maintain low levels of the protein. Indeed, in human cell lines originating from various tissues, this appears to be the case as *Hs*PEX39 was undetectable by whole-cell proteomics of cells from the Cancer Cell Line Encyclopedia^40^, even though *HsPEX39* messenger RNA was present (Extended Data Fig. 5f).

Consistent with our findings in human cells, yeast overexpressing *Sc*Pex39 had reduced growth in oleic acid medium (Fig. 3e), but not in glucose medium (Extended Data Fig. 5g). Notably, Pex7 levels were not altered in yeast overexpressing *Sc*Pex39 (Extended Data Fig. 3e, lane 1 versus 5). Interestingly, cellular fractionation of yeast overexpressing *Sc*Pex39 revealed increased Pex7 in the organellar fraction relative to wild-type yeast, but no such effect was observed for the PTS2-containing proteins Pot1 and Gpd1 or the PTS1-containing protein Cta1 (Fig. 3f and Extended Data Fig. 5h). Based on these findings, we reason that a fraction of Pex7 with its PTS2-containing protein cargo is stalled at the peroxisomal membrane (and not imported) in the presence of excess *Sc*Pex39. To further investigate this, we conducted sodium carbonate extraction and sequential treatment of organellar pellets with low- and high-salt buffer (Extended Data Fig. 5i). Although Pot1 and Pex7 were not carbonate resistant (that is, not integral membrane proteins), they remained in the organellar pellet after extraction with low-salt but not high-salt buffer. Thus, we conclude that the stalled fraction of Pot1 and Pex7 is anchored at the peroxisomal membrane via protein–protein interactions when *Sc*Pex39 levels are elevated.

### PEX39 stabilizes the interaction between PEX7 and PTS2-containing proteins

**Fig. 4:**
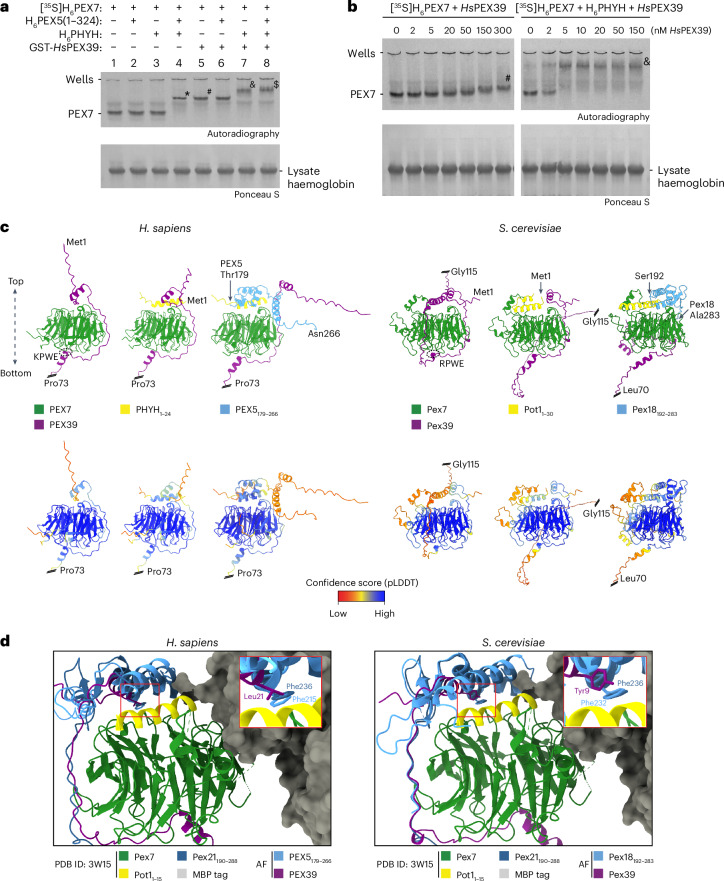
PEX39 stabilizes the interaction between PEX7 and PTS2-containing proteins. **a**, Native PAGE and autoradiography of [^35^S]H_6_PEX7 pre-incubated with the recombinant proteins H_6_PEX5(1–324), H_6_PHYH and GST-*Hs*PEX39, as indicated. In-gel positions of PEX7 alone and the complexes PEX7–PEX5–PHYH (asterisk), PEX7–*Hs*PEX39 (hashtag), PEX7–PHYH–*Hs*PEX39 (ampersand) and PEX7–PEX5–PHYH–*Hs*PEX39 (dollar sign) are indicated. **b**, Assessments of *K*_d,app_ for *Hs*PEX39–PEX7 interaction in different complexes in vitro. [^35^S]H_6_PEX7 was incubated with increasing amounts of recombinant *Hs*PEX39 in the absence or presence of H_6_PHYH and then analysed by native PAGE and autoradiography. The recombinant *Hs*PEX39 used here lacks a GST tag. In-gel positions of PEX7 alone and the complexes PEX7–*Hs*PEX39 (hashtag) and PEX7–PHYH–*Hs*PEX39 (ampersand) are indicated. **c**, AlphaFold predictions of human and yeast PEX39-containing complexes. The top and bottom faces of PEX7 are oriented as indicated. Top: in PEX7/PEX39 dimers, the (R/K)PWE motifs of PEX39 are marked with a dashed circle. Structure predictions were performed with full-length PEX39. However, for visualization, PEX39 has been C-terminally shortened, as indicated by a double line and the last amino acid. Bottom: AlphaFold confidence scores (that is, strengths of the structural predictions) for the corresponding models above, shown according to the predicted local distance difference test (pLDDT). **d**, Comparison of the AlphaFold models with the crystal structure of the yeast Pex7–Pex21–Pot1 complex. The crystal structure (Protein Data Bank (PDB) ID 3W15; ref. ^44^) of Pex7 in complex with Pex21_190–288_ and Pot1_1–15_ (PTS2) fused to a maltose-binding protein tag (MBP tag) was superimposed with the trimeric and tetrameric AlphaFold (AF) models shown in **c** using ChimeraX. Single entities of the used structural models are coloured as indicated. For simplification, PEX39 from tetrameric complexes and PTS2 helices and PEX7 orthologues of the AlphaFold models are not displayed. Insets: magnifications of the structurally conserved hydrophobic residues *Hs*PEX39 Leu21 with *Sc*Pex21 Phe236 and *Hs*PEX5 Phe215 (left) and *Sc*Pex39 Tyr9 with *Sc*Pex21 Phe236 and *Sc*Pex18 Phe232 (right). Source data

**Extended Data Fig. 6:**
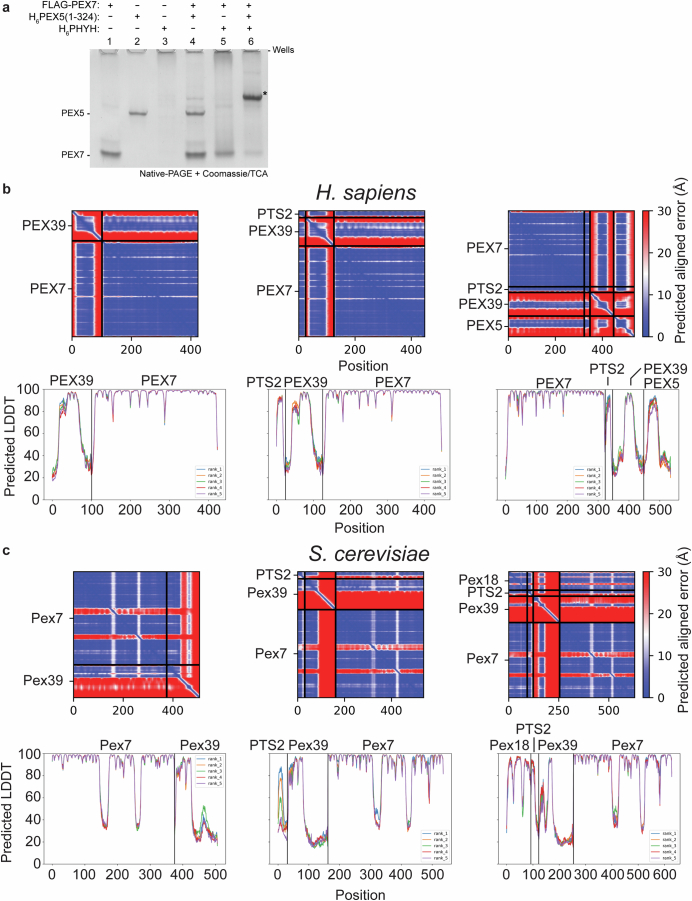
Assessment of PEX7 complex formation and confidence measures of predicted AlphaFold modelling. **a**, Native-PAGE and Coomassie/TCA staining of the indicated recombinant proteins. In-gel positions of PEX7 alone (PEX7), PEX5 alone (PEX5), and the PEX7-PEX5-PHYH complex (*) are indicated. H_6_PHYH is a basic protein that does not enter these gels alone^33^. **b**, Confidence measures for human protein complex predictions. Predicted aligned error plots and predicted LDDT (local distance difference test) plots of human AlphaFold models. To predict human protein complexes, PEX39 (full-length), PEX7 (full-length), the PTS2 sequence of PHYH (amino acids 1-24), and the PEX7 binding domain of the long isoform of PEX5 (amino acids 179-266) were used. **c**, Confidence measures for yeast protein complex predictions. Same plots as described in **b** for predictions of yeast protein complexes. Protein complexes from yeast were predicted using *Sc*Pex39 (full-length), Pex7 (full-length), the PTS2 sequence of Pot1 (amino acids 1-30), and the Pex7 binding domain of Pex18 (amino acids 192-283). Source data

To gain insight into the mechanisms underlying the biology of PEX39, we utilized our native PAGE assays. Using radiolabelled PEX7, we observed that the interactions between PEX7 and PEX5 or PEX7 and PHYH are too weak to be detected by native PAGE (Fig. 4a, lanes 2 and 3, respectively), whereas the PEX7–PEX5–PHYH complex is stable and readily detected (asterisk in lane 4). Similar observations were made using recombinant FLAG-PEX7 (Extended Data Fig. 6a). Of note, the fact that the addition of recombinant *Hs*PEX39 allowed for a PEX7–PHYH–*Hs*PEX39 complex to form (Fig. 4a, ampersand in lane 7) demonstrates that *Hs*PEX39 stabilizes the weak PEX7–PHYH interaction.

Using this native PAGE assay, we next determined the apparent dissociation constants (*K*_d,app_ values) for the *Hs*PEX39–PEX7 interaction in the presence or absence of PHYH. Although these *K*_d,app_ values are not direct measurements of PEX7 and *Hs*PEX39 binding, they can qualitatively offer valuable information. Both the PEX7–*Hs*PEX39 and PEX7–PHYH–*Hs*PEX39 complexes exhibited *K*_d,app_ values for *Hs*PEX39 in the nanomolar range, reflecting high-affinity interactions (Fig. 4b). Interestingly, the PEX7–PHYH–*Hs*PEX39 complex formed at lower *Hs*PEX39 concentrations (Fig. 4b, right) than those seen for formation of the PEX7–*Hs*PEX39 complex (Fig. 4b, left), supporting our observation that *Hs*PEX39 stabilizes the interaction between PEX7 and PTS2-containing proteins.

To gain further insights, we used AlphaFold^41,42^ to predict the structure of *Hs*PEX39 in complex with human PEX7, PHYH(1–24) (the PTS2-containing portion) or PEX5(179–266) (PEX7-binding residues^43^) (Fig. 4c and Extended Data Fig. 6b). These structural predictions corroborate our experimental finding that *Hs*PEX39 stabilizes the PEX7–PHYH interaction by predicting that *Hs*PEX39 binds the base of PEX7 via the conserved KPWE motif and the N-terminal half wraps around PEX7 to interact with the PTS2, thereby potentially acting as a clamp to strengthen the PEX7–PHYH interaction. Notably, beginning with the model of the PEX7–*Hs*PEX39 complex, the addition of PHYH(1–24) is predicted to introduce a new binding surface for the N-terminal region of *Hs*PEX39, which agrees with the observed increase in binding affinity between PEX7 and *Hs*PEX39 (that is, decreased *K*_d,app_) when PHYH is present (Fig. 4b). PEX5(179–266) is also predicted to interact with the PTS2 and displace the N-terminal portion of *Hs*PEX39 but still allow for *Hs*PEX39 to bind PEX7 via the KPWE motif, consistent with our observations of a PEX7–PHYH–*Hs*PEX39–PEX5 complex. AlphaFold modelling of *Sc*Pex39 in complex with yeast Pex7, Pot1(1–30) (the PTS2-containing portion) or Pex18(192–283) (Pex7-binding residues^43^) revealed similar predictions (Fig. 4c and Extended Data Fig. 6c).

Importantly, the AlphaFold predictions are consistent with the crystal structure of a yeast complex between Pex7, Pex21 and a PTS2-containing protein^44^ (Fig. 4d). As in PEX5 (human) and Pex18/Pex21 (yeast), the N terminus of human and yeast PEX39 covers the PTS2 bound to Pex7. Of note, we identified the conserved N-terminal hydrophobic residues L21 (*Hs*PEX39) and Y9 (*Sc*Pex39) as being positioned over the PTS2 similarly to residues of yeast and human co-receptors, namely F236 (Pex21), F232 (Pex18) and F215 (PEX5) (Fig. 4d, insets). Pex21 F236 is critical for stabilizing the interaction between Pex7 and a PTS2-containing protein^44^, suggesting a similar role for the aforementioned residues in PEX39. Collectively, our data indicate that PEX39 stabilizes the interaction between PEX7 and PTS2-containing proteins, thereby providing a mechanism by which PEX39 facilitates the import of PTS2-containing proteins.

### Identification of residues necessary for PEX39 function

**Fig. 5:**
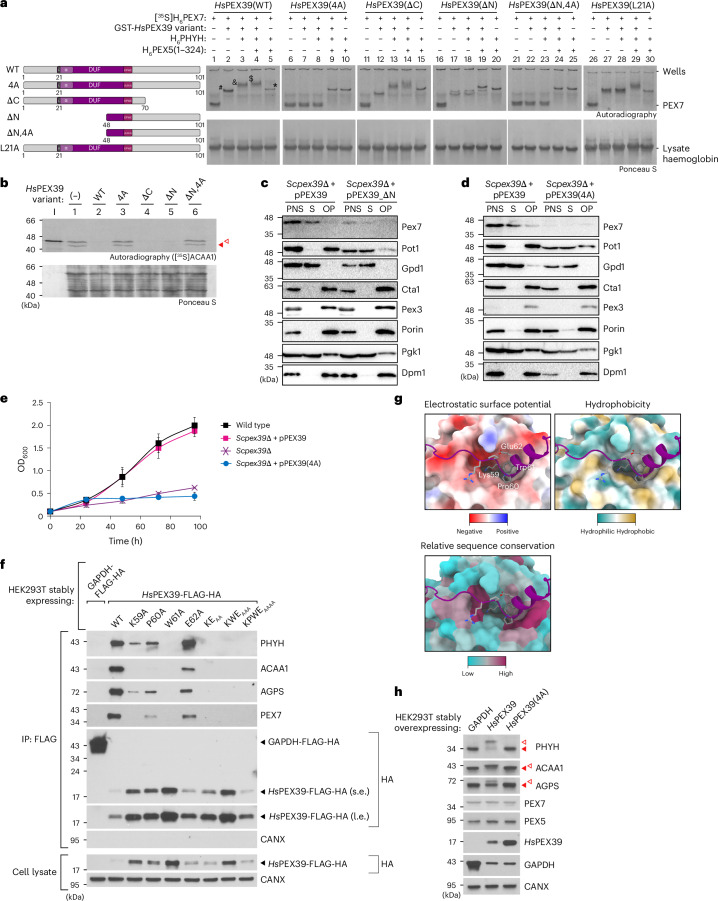
The N-terminal region and (R/K)PWE motif are essential for PEX39 function. **a**, Native PAGE and autoradiography of [^35^S]H_6_PEX7 pre-incubated with the indicated recombinant *Hs*PEX39 variants (depicted on the left). In-gel positions of PEX7 alone and the complexes PEX7–*Hs*PEX39 (hashtag), PEX7–*Hs*PEX39–PHYH (ampersand), PEX7–PEX5–PHYH–*Hs*PEX39 (dollar sign) and PEX7–PEX5–PHYH (asterisk) are indicated for full-length wild-type (WT) *Hs*PEX39. Double bands of *Hs*PEX39(ΔN) complexes are due to co-migration with haemoglobin from the reticulocyte lysate. **b**, [^35^S]ACAA1 in vitro import assays in the absence (−) or presence of the indicated recombinant *Hs*PEX39 variants (depicted in **a**). The other details of the experiment were as described for Fig 3c. **c**, Immunoblots of cellular fractions from yeast expressing N-terminally truncated *Sc*Pex39. The experiment was performed as described for Fig. 2b using *Scpex39*Δ cells transformed with plasmids encoding wild-type *Sc*Pex39 (pPEX39) or a variant comprising residues 40–132 (pPEX39_ΔN). **d**, Immunoblots of cellular fractions from yeast expressing a variant of *Sc*Pex39. The experiment was performed as described for Fig. 2b using *Scpex39*Δ cells transformed with plasmids encoding wild-type *Sc*Pex39 (pPEX39) or an *Sc*Pex39 RPWE-to-AAAA variant (pPEX39(4A)). In **c** and **d**, all of the plasmids utilized the endogenous *Scpex39* promoter. **e**, Growth of the indicated yeast strains in oleic acid medium. The data for the wild type, *Scpex39*Δ and *Scpex39*Δ + pPEX39 are the same as those shown in Fig. 2a. The data represent means ± s.d. (*n* = 4). **f**, Immunoblots of anti-FLAG immunoprecipitates prepared from HEK293T cells expressing GAPDH-FLAG-HA (negative control) or the indicated *Hs*PEX39 variants (single or multiple alanine replacements of the indicated residues). **g**, Close-up views of the interactions between the *Hs*PEX39 KPWE motif (labelled) and PEX7. Shown are the different surface properties and relative sequence conservation of the *Hs*PEX39 binding region of human PEX7. The images are based on the same structural model shown in Fig. 4c (*H. sapiens*). **h**, Immunoblot analysis of HEK293T cells overexpressing GAPDH, *Hs*PEX39 or *Hs*PEX39(4A). The solid and open red arrowheads indicate mature and precursor forms, respectively. DUF, DUF5572. Source data

The evolutionary conservation of the (R/K)PWE motif in PEX39 orthologues (Extended Data Fig. 1a) and the AlphaFold prediction of this motif being a PEX7-binding site (Fig. 4c) suggested that it contributes to PEX39 function. To investigate this, we utilized an *Hs*PEX39 variant with the KPWE motif changed to AAAA (*Hs*PEX39(4A)), as well as different variants of *Hs*PEX39 with designs influenced by insights gained from the AlphaFold predictions. Using our native PAGE assays with [^35^S]H_6_PEX7, we examined the ability of these *Hs*PEX39 variants to complex with PEX7, PHYH and PEX5 (Fig. 5a). *Hs*PEX39(4A) could not interact with PEX7 and failed to stabilize the interaction between PEX7 and the PTS2-containing protein PHYH, as the only detectable species were PEX7 alone or the PEX7–PHYH–PEX5 complex (lanes 6–10). A C-terminally truncated version of *Hs*PEX39 containing residues 1–70 (*Hs*PEX39(ΔC)) could still complex normally (lanes 11–15), consistent with the deleted residues not being involved in the formation of the corresponding complexes per AlphaFold (Fig. 4c). Using an N-terminally truncated variant containing residues 48–101 ((*Hs*PEX39(ΔN)), we observed the formation of a PEX7–*Hs*PEX39(ΔN) complex (Fig. 5a, lane 17) but not a PEX7–*Hs*PEX39(ΔN)–PHYH complex (lane 18 versus 17), indicating that the missing N-terminal region stabilizes the weak PEX7–PHYH interaction, consistent with the AlphaFold predictions (Fig. 4c). Within this N-terminal region, we identified L21 to be potentially needed for stabilizing the interaction between PEX7 and PTS2-containing proteins (Fig. 4d). Indeed, alanine replacement of L21 (*Hs*PEX39(L21A)) was sufficient to abolish the interaction with PHYH (Fig. 5a, lanes 2 and 3 versus 27 and 28). Both *Hs*PEX39(ΔN) and *Hs*PEX39(L21A) interacted with the PEX7–PHYH–PEX5 complex (lanes 18–20 and 28–30), suggesting that the KPWE motif is sufficient for this interaction. Indeed, *Hs*PEX39(ΔN) with the KPWE motif changed to AAAA (*Hs*PEX39(ΔN,4A)) failed to interact with PEX7, PHYH and PEX5 (lanes 21–25). Further underscoring the importance of the KPWE motif, all exogenously added *Hs*PEX39 variants with an intact KPWE motif and the ability to bind PEX7 in vitro (Fig. 5a) almost completely inhibited in vitro import of [^35^S]ACAA1 (Fig. 5b, lanes 2, 4 and 5), whereas variants with the KPWE motif changed to AAAA and an inability to bind PEX7 in vitro did not affect import (lanes 3 and 6).

**Extended Data Fig. 7:**
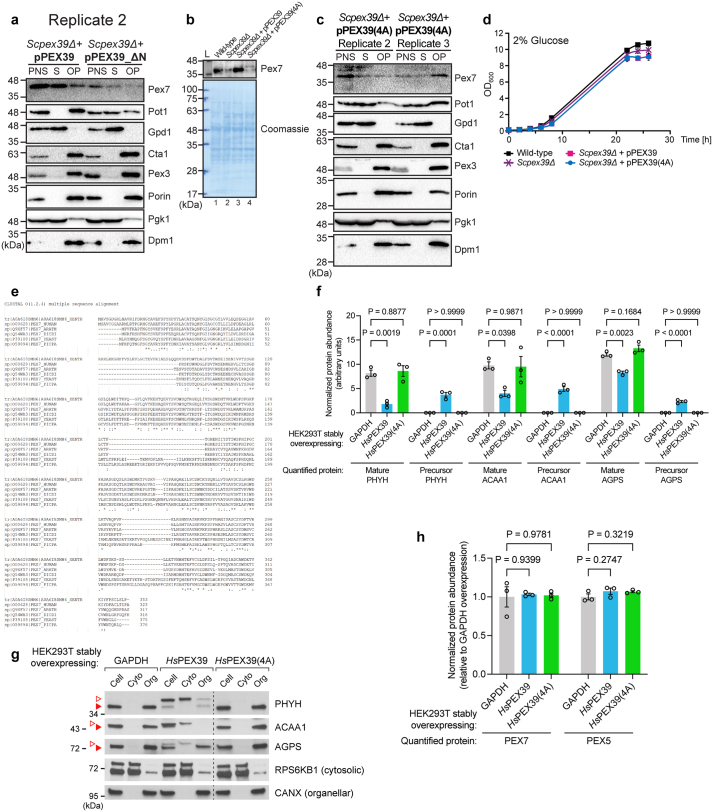
Characterization of N-terminal region and [R/K]PWE motif of PEX39. **a**, Biological replicate for experiment in Fig. 5c. **b**, Immunoblots of post-nuclear supernatants from the indicated yeast strains. *Scpex39Δ* cells were transformed with plasmids for expression of wild-type *Sc*Pex39 (pPEX39) or the *Sc*Pex39 RPWE-to-AAAA mutant [pPEX39(4A)], each under control of the endogenous promoter. **c**, Biological replicates for cellular fractionation of *Scpex39Δ* cells expressing the *Sc*Pex39 RPWE-to-AAAA mutant. Experiments were performed on the indicated strains as described in Fig. 5d. **d**, Growth of the indicated yeast strains in glucose medium. Data for wild-type, *Scpex39Δ*, and *Scpex39Δ* + pPEX39 are the same as shown in Extended Data Fig. 3b. Data are mean ± SD (n = 3). **e**, Multiple sequence alignment of PEX7 orthologs from the indicated species via ClustalΩ (v.1.2.4), which was used for coloring of PEX7 sequence conservation in Fig. 5g. **f**, Quantification of changes in mature and precursor forms using band intensities of immunoblots prepared per Fig. 5h. Precursor and mature form band intensities were normalized to those of loading controls. The data for cells overexpressing GAPDH and *Hs*PEX39 are the same as shown previously in Fig. 3b. All overexpression lines were processed together in the same experiments to generate these data. **g**, Immunoblots of whole-cell (Cell), cytosolic (Cyto), and organellar (Org) fractions from the indicated cells. Solid and open red arrowheads indicate mature and precursor forms, respectively. Dashed lines indicate where different lanes of the same membrane were brought together. The data for cells overexpressing GAPDH and *Hs*PEX39 are the same as shown previously in Extended Data Fig. 5b. All overexpression lines were processed together in the same experiment to generate these data. **h**, Quantification of PEX7 and PEX5 band intensities from immunoblots prepared per Fig. 5h. PEX7 and PEX5 band intensities were normalized to those of loading controls. Normalized protein abundances were then divided by the mean of the cells overexpressing GAPDH. The data for cells overexpressing GAPDH and *Hs*PEX39 are the same as shown previously in Extended Data Fig. 5c. All overexpression lines were processed together in the same experiments to generate these data. **f**,**h**, Data are mean ± SEM (n = 3). Multiplicity-adjusted P values were calculated using ordinary, one-way ANOVA with Dunnett’s multiple comparisons test. Source data

Next, we extended our investigations into yeast and human cells. Expression via the endogenous promoter of an N-terminally truncated variant of *Sc*Pex39 that retains the RPWE motif and comprises residues 40–132 (*Sc*Pex39_ΔN) failed to restore both Pex7 levels (Extended Data Fig. 3e, lane 4 versus 6) and the import of PTS2-containing proteins in *Scpex39*Δ yeast (Fig. 5c and Extended Data Fig. 7a). Similar experiments using *Sc*Pex39 with the RPWE motif mutated to AAAA (*Sc*Pex39(4A)) also failed to restore Pex7 levels (Fig. 5d and Extended Data Fig. 7b) and the import of PTS2-containing proteins (Fig. 5d and Extended Data Fig. 7c). *Sc*Pex39(4A) failed to rescue the fitness defect of *Scpex39*Δ cells grown in oleic acid medium (Fig. 5e), whereas growth in glucose medium remained unaffected (Extended Data Fig. 7d). Using HEK293T cells, we observed that alanine replacement of any single residue within the KPWE motif generally had a deleterious effect on the ability of *Hs*PEX39 to interact with its binding partners, with the W61A variant most impaired and the E62A variant least impaired (Fig. 5f). The AlphaFold prediction of the PEX7-binding site and KPWE motif corroborates the importance of the motif residues by illustrating how conserved PEX7 residues with matching biochemical properties are positioned to interact (Fig. 5g and Extended Data Fig. 7e). Furthermore, whereas overexpression of wild-type *Hs*PEX39 increased precursor forms and decreased mature forms of PHYH, ACAA1 and AGPS, overexpression of *Hs*PEX39(4A) did not (Fig. 5h and Extended Data Fig. 7f). Cellular fractionation confirmed that overexpression of *Hs*PEX39(4A) did not inhibit the import of PTS2-containing proteins (Extended Data Fig. 7g). Lastly, overexpression of the wild type or *Hs*PEX39(4A) did not alter the levels of PEX7 or PEX5 (Fig. 5h and Extended Data Fig. 7h).

### The PEX13 KPWE motif is necessary for proper PEX13 function

**Fig. 6:**
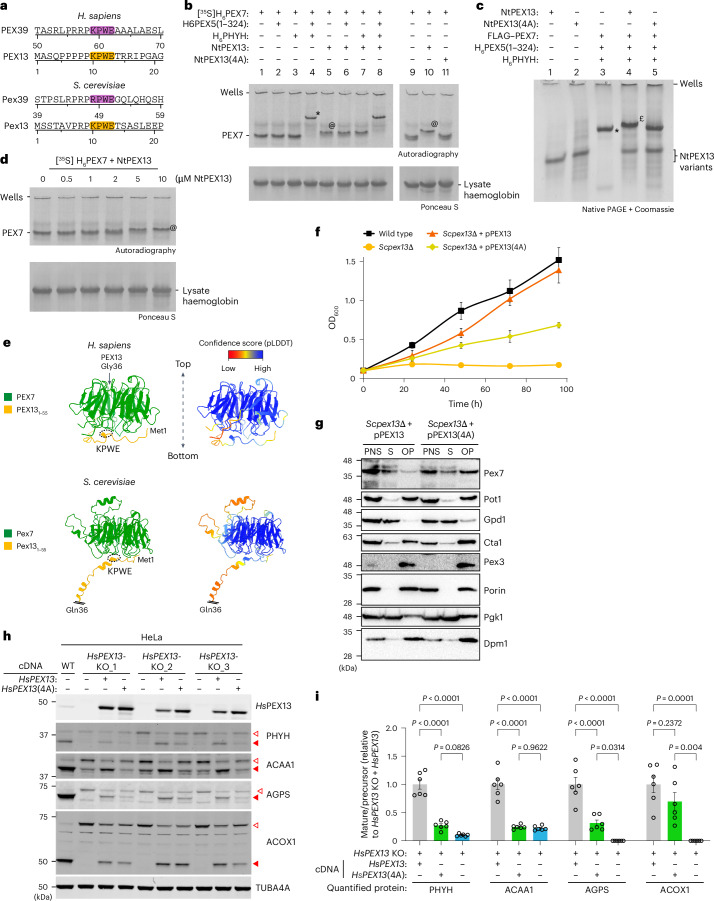
The N-terminal KPWE motif of PEX13 is necessary for proper PEX13 function. **a**, Schematic of the (R/K)PWE motifs in PEX39 and the N terminus of PEX13. **b**, Native PAGE and autoradiography of [^35^S]H_6_PEX7 pre-incubated with the indicated recombinant proteins. NtPEX13 represents *Hs*PEX13 residues 1–36 fused to Sumo1 with a hexa-histidine tag. NtPEX13(4A) represents NtPEX13 with a KPWE-to-AAAA substitution. The in-gel positions of PEX7 alone and the complexes PEX7–PEX5–PHYH (asterisk) and PEX7–*Hs*PEX13 (at symbol) are indicated. **c**, Native PAGE and Coomassie staining of different recombinant proteins alone or mixed together, as indicated. The in-gel positions of the complexes PEX7–PEX5–PHYH (asterisk) and PEX7–PEX5–PHYH–PEX13 (pound sign) are indicated. **d**, Assessment of *K*_d,app_ for the interaction between the N terminus of *Hs*PEX13 and PEX7. [^35^S]H_6_PEX7 was incubated with increasing amounts of NtPEX13 and analysed by native PAGE and autoradiography. The in-gel positions of PEX7 alone and the complex PEX7–*Hs*PEX13 (at symbol) are indicated. **e**, AlphaFold prediction of interactions between the PEX13 N terminus and PEX7. The top and bottom faces of PEX7 are oriented as indicated. Structural modelling was performed using PEX13 residues 1–55, but for visualization PEX13 was C-terminally shortened at residue 36. **f**, Growth of the indicated yeast strains in oleic acid medium. *Scpex13*Δ cells were transformed with plasmids encoding wild-type *Sc*Pex13 (pPEX13) or an *Sc*Pex13 variant with a KPWE-to-AAAA substitution (pPEX13(4A)), each under the control of the endogenous promoter. The data represent means ± s.d. (*n* = 4, except for the wild type at 96 h (*n* = 3)). **g**, Immunoblots of cellular fractions from the indicated yeast strains (as described in **f**). The experiment was performed as described for Fig. 2b. **h**, Immunoblot analysis of three independent *HsPEX13*-KO HeLa clones transfected with plasmid cDNA for wild-type *HsPEX13* or an *HsPEX13* mutant encoding the KPWE-to-AAAA substitution (*HsPEX13*(4A)). Solid and open red arrowheads indicate mature and precursor forms, respectively. TUBA4A (α-tubulin) was used as a loading control. **i**, Quantification of mature and precursor forms using the immunoblot band intensities from experiments as described in **h**. Mature/precursor ratios were calculated for the indicated proteins and then divided by the mean value of the *HsPEX13*-KO + *HsPEX13* replicates. The data represent means ± s.e.m. (*n* = 6). Multiplicity-adjusted *P* values were calculated using ordinary, one-way analysis of variance with Tukey’s multiple comparisons test. Source data

**Extended Data Fig. 8:**
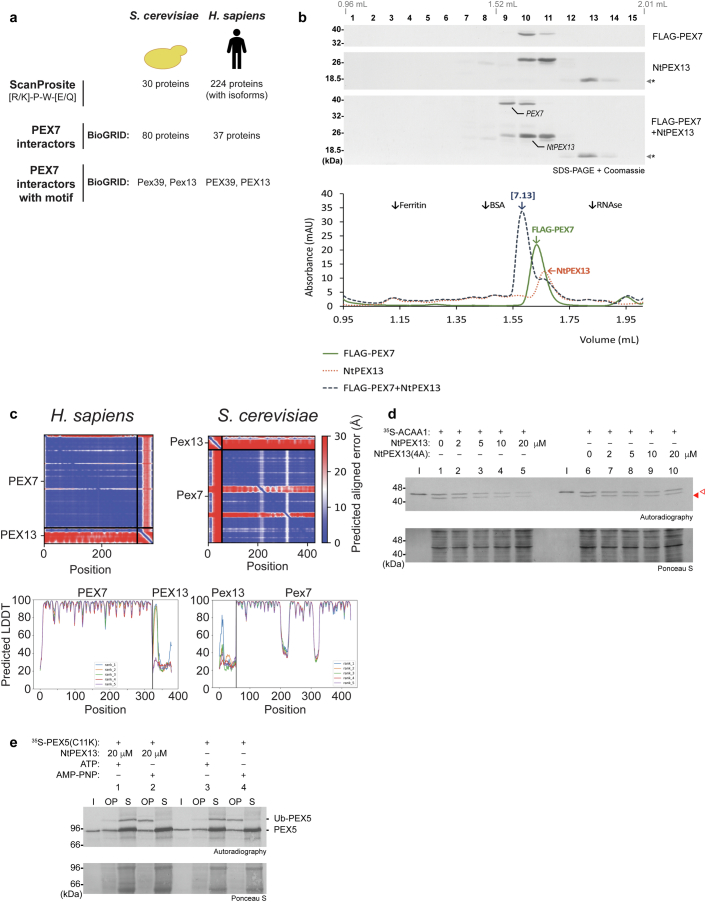
Characterization of the KPWE motif in the N-terminus of PEX13. **a**, ScanProsite analysis of generic [R/K]-P-W-[E/Q] motifs in yeast and humans. The UniProtKB sequence databases of yeast or human were searched for proteins harboring the short linear motif [R/K]-P-W-[E/Q] using the webtool ScanProsite (https://prosite.expasy.org/scanprosite/). Hits were then compared with proteins listed as yeast and human PEX7 interactors in the BioGRID database. **b**, Recombinant FLAG-PEX7 and NtPEX13, alone or mixed together, were analyzed by SEC. Collected fractions were analyzed by SDS-PAGE and Coomassie staining (top). The corresponding area of the chromatogram is also shown (bottom). The elution volumes of the PEX7-NtPEX13 complex, the individual recombinant proteins, and molecular weight standards (Ferritin, BSA, RNAse A) are indicated. The asterisk indicates a proteolytic product of NtPEX13. **c**, Confidence measures for modeling the PEX7-PEX13 interaction for human and yeast orthologs. Predicted aligned error plots (top) and predicted LDDT (local distance difference test) plots (bottom) of human (left) and yeast (right) AlphaFold models. To predict human and yeast protein complexes, full-length PEX7 and the N-terminus of PEX13 (residues 1-55) were used. **d**, ^35^S-ACAA1 *in vitro* import assays in the presence of increasing concentrations of NtPEX13 or NtPEX13(4A). After incubation, reactions were treated with trypsin and organelles were isolated by centrifugation and analyzed by SDS-PAGE and autoradiography. Precursor and mature ACAA1 indicated by open and solid red arrowheads, respectively. I, input (5% of the reticulocyte lysate containing the ^35^S-ACAA1 used in each reaction). **e**, ^35^S-PEX5(C11K) *in vitro* import assays in the presence or absence of NtPEX13. After incubation, organelle pellet (OP) and cytosolic supernatant (S) fractions were isolated by centrifugation and analyzed by SDS-PAGE and autoradiography. Unmodified ^35^S-PEX5(C11K) (“PEX5”) and monoubiquitinated ^35^S-PEX5(C11K) (“Ub-PEX5”) are indicated. AMP-PNP inhibits the ATPases PEX1/PEX6 and thereby inhibits the transfer of monoubiquitinated ^35^S-PEX5(C11K) from peroxisomes into the cytosolic supernatant^115^. I, input [5% of the reticulocyte lysate containing the ^35^S-PEX5(C11K) used in each reaction]. Source data

A conserved KPWE motif is also present in the N terminus of PEX13 (Extended Data Fig. 1a and Fig. 6a), but has not been experimentally characterized. Interestingly, the presence or absence of PEX39 is mirrored by the presence or absence of the PEX13 KPWE motif across eukaryotes (Extended Data Fig. 1a), strongly suggesting a functional connection between PEX39 biology and the PEX13 KPWE motif throughout evolution. In addition, among PEX7 interactors in yeast and humans, the (R/K)PWE motif is exclusively found in PEX39 and PEX13 (Extended Data Fig. 8a). These insights are notable for several reasons: PEX13 is essential for peroxisomal protein import^45^ and is thought to form a conduit for proteins to translocate across the peroxisomal membrane^23,24^; residues 1–55 of Pex13, which contain the KPWE motif, can bind Pex7 and are essential for the import of PTS2-containing proteins in yeast^46^; and recently, computational predictions suggested that the PEX13 KPWE motif could bind PEX7 (ref. ^47^). We thus hypothesized that the PEX13 KPWE motif is necessary for binding PEX7 and plays an important role in PEX13 function.

To test this hypothesis, we assayed a recombinant protein comprising residues 1–36 of the *Hs*PEX13 N terminus fused to the small ubiquitin-related modifier 1 (Sumo1) with a hexa-histidine tag (NtPEX13) and a variant with the KPWE motif substituted with AAAA (NtPEX13(4A)) in our native PAGE assay and observed that [^35^S]H_6_PEX7 can indeed interact with NtPEX13 (Fig. 6b, lanes 5 versus 1 and 10 versus 9). The formation of a PEX7–NtPEX13 complex could also be detected by SEC (Extended Data Fig. 8b). Importantly, NtPEX13(4A) failed to interact with PEX7 (Fig. 6b, lane 10 versus 11), demonstrating the necessity of the KPWE motif for PEX7 binding. The PEX7–NtPEX13 complex was unchanged by the presence of PEX5(1–324) or PHYH (Fig. 6b, lanes 5–7) and only if both were present did we observe the appearance of a slower migrating complex, although we could not discern whether this was PEX7–PHYH–PEX5 alone or with NtPEX13 bound as well (Fig. 6b, lane 4 versus 8). However, replacing [^35^S]H_6_PEX7 with recombinant FLAG-PEX7 allowed us to observe the binding of NtPEX13 to the PEX7–PHYH–PEX5 complex, which again depended on the presence of the PEX13 KPWE motif (Fig. 6c).

We also determined *K*_d,app_ for the PEX7–NtPEX13 interaction to be in the low micromolar range (Fig. 6d), which was larger than that observed for the PEX7–*Hs*PEX39 interaction (Fig. 4b, left). However, interpretation of this value should be with the understanding that we did not use full-length *Hs*PEX13 and did not reconstitute the complete peroxisomal docking/translocation module, in which PEX13 should be present in multiple copies^16,48^. Consistent with our in vitro data, AlphaFold modelling predicted that the PEX13 KPWE motif binds to the bottom of PEX7 (Fig. 6e and Extended Data Fig. 8c). Interestingly, as for *Hs*PEX39, NtPEX13 inhibited the import of [^35^S]ACAA1 in vitro in a KPWE-dependent fashion (Extended Data Fig. 8d), but not the import pathway of PTS1-containing proteins (Extended Data Fig. 8e).

**Extended Data Fig. 9:**
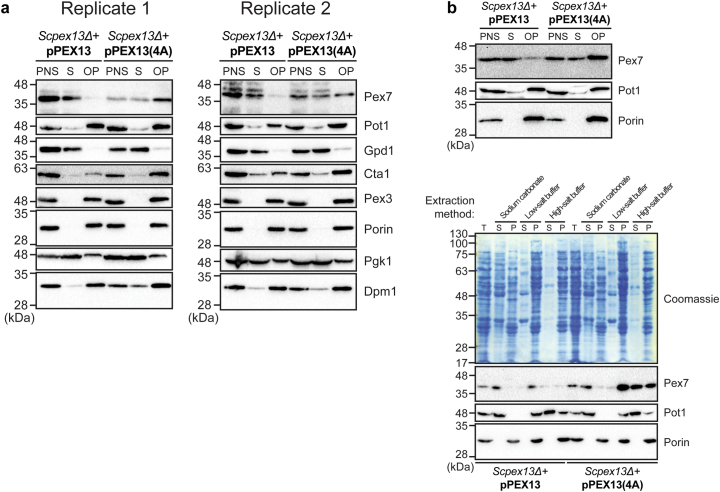
Characterization of *Sc*Pex13 with a mutated KPWE motif. **a**, Biological replicates of the cellular fractionation of yeast expressing the *Sc*Pex13 KPWE-to-AAAA mutant. Experiments were performed on the indicated strains as described in Fig. 6g. **b**, Characterization of organelle-associated Pex7 and Pot1 in yeast expressing the *Sc*Pex13 KPWE-to-AAAA mutant. The strains used are described in Fig. 6g. Top: immunoblots of cellular fractionation of yeast expressing the *Sc*Pex13 KPWE-to-AAAA mutant as described for Fig. 6g. Bottom: immunoblot analysis of salt-extraction of proteins from organellar pellets isolated as described for Fig. 6g. T, total; S, supernatant fraction; P, pellet fraction. Source data

Next, we investigated the PEX13 KPWE motif in yeast and human cells. Expression of *Sc*Pex13 with the KPWE motif substituted with AAAA could not fully rescue the fitness defect of *Scpex13*Δ yeast grown in oleic acid medium (Fig. 6f). Interestingly, cellular fractionation of these yeast revealed an increased amount of Pex7 in the organellar fraction relative to control yeast (Fig. 6g and Extended Data Fig. 9a). Protein extraction experiments revealed that organellar Pex7 and Pot1 were not carbonate resistant and remained in the organellar pellet after extraction with low-salt but not high-salt buffer (Extended Data Fig. 9b). These results resemble those for yeast overexpressing *Sc*Pex39 and we similarly conclude that a fraction of Pex7 with its PTS2-containing protein cargo is stalled at the peroxisomal membrane and anchored there via protein–protein interactions (but not imported) upon loss of the *Sc*Pex13 KPWE motif. In *HsPEX13*-KO human cells, whereas the expression of wild-type *Hs*PEX13 or a variant with the KPWE motif substituted with AAAA (*Hs*PEX13(4A)) increased the ratio of mature to precursor forms of the PTS1-containing protein ACOX1 to a similar degree, *Hs*PEX13(4A) was notably worse at increasing the ratios of mature to precursor forms for all PTS2-containing proteins (Fig. 6h,i), thus indicating that substitution of the KPWE motif selectively impairs the import of PTS2-containing proteins, but not that of PTS1-containing proteins.

### A PEX7 glutamate is necessary for binding PEX39 and PEX13

**Fig. 7:**
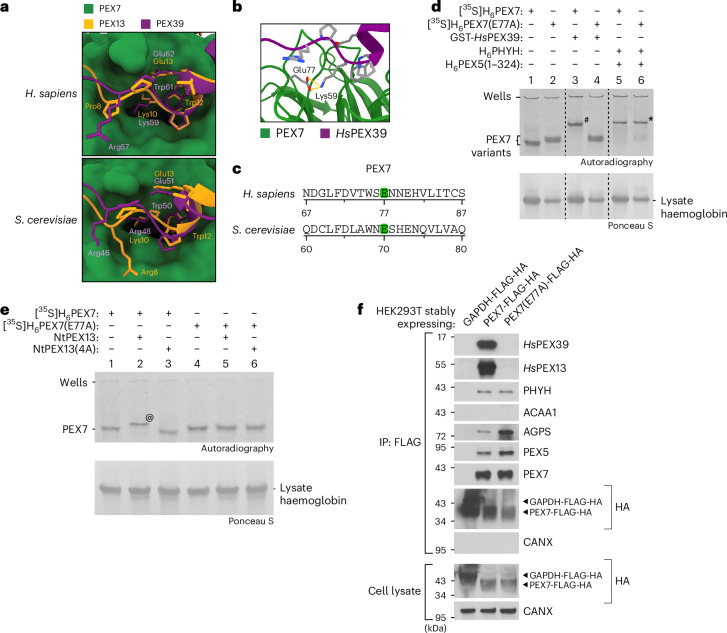
A conserved glutamate residue in PEX7 is necessary for interaction with PEX39 and PEX13. **a**, Structural modelling predicts that (R/K)PWE motifs of PEX39 and the N terminus of PEX13 bind to the same site of PEX7. Shown are superimpositions of the predicted models shown in Figs. 4c and 6e. The residues of the PEX39 and PEX13 (R/K)PWE motifs are shown. **b**, AlphaFold modelling of the predicted salt bridge between human PEX7 Glu77 and *Hs*PEX39 Lys59. The distance (yellow dotted line) between the terminal oxygens of PEX7 Glu77 and the zeta nitrogen of *Hs*PEX39 Lys59 measures 3.9 Å (top) and 4.1 Å (bottom). **c**, Schematic demonstrating that Glu77 of human PEX7 is conserved in yeast Pex7. Sequence alignment of PEX7 orthologues was performed using Clustal Omega (version 1.2.4) (see Extended Data Fig. 7e for complete alignment). Relevant glutamate residues are highlighted in green. **d**, Native PAGE and autoradiography of wild-type [^35^S]H_6_PEX7 or [^35^S]H_6_PEX7(E77A) pre-incubated with the indicated recombinant proteins. The in-gel positions of the PEX7 variants alone and the complexes PEX7–*Hs*PEX39 (hashtag) and PEX7–PEX5–PHYH (asterisk) are indicated. The dashed lines indicate where different lanes of the same membrane were brought together. **e**, Native PAGE and autoradiography of wild-type [^35^S]H_6_PEX7 or [^35^S]H_6_PEX7(E77A) pre-incubated with NtPEX13 or NtPEX13(4A). The in-gel positions of the PEX7 variants alone and the PEX7–*Hs*PEX13 complex (at symbol) are indicated. **f**, Immunoblots of anti-FLAG immunoprecipitates prepared from HEK293T cells expressing the indicated proteins. Source data

AlphaFold predicts that the (R/K)PWE motifs of PEX39 and PEX13 bind the same site on PEX7 in both human and yeast (Fig. 7a) and that E77 in human PEX7 can form a salt bridge with the K59 residue of the KPWE motif (Fig. 7b), thus suggesting a critical role for E77, which is also conserved in yeast and other organisms (Fig. 7c and Extended Data Fig. 7e). Consistent with this, PEX7 harbouring an E77A substitution (PEX7(E77A)) did not interact in our native PAGE assays with *Hs*PEX39 (Fig. 7d, lanes 1–4) or NtPEX13 (Fig. 7e). Importantly, PEX7(E77A) still interacted with PEX5 and PHYH (Fig. 7d, lane 5 versus 6), indicating that it was correctly folded. Using HEK293T cells, we also observed that PEX7(E77A)-FLAG-HA was unable to co-immunoprecipitate *Hs*PEX39 and *Hs*PEX13, but still interacted with PEX5, PHYH and AGPS (Fig. 7f).

### Dissociation of PEX39 allows PEX7 to bind the PEX13 N terminus

**Fig. 8:**
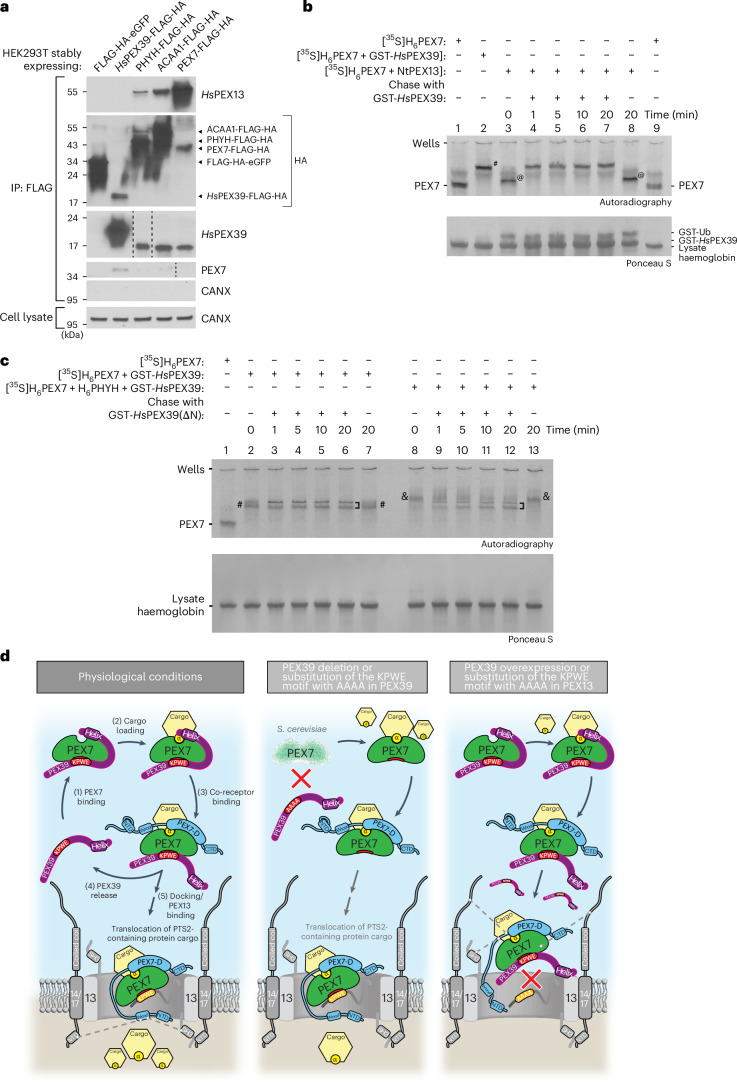
Dissociation of PEX39 from PEX7 allows the N terminus of PEX13 to bind. **a**, Immunoblots of anti-FLAG immunoprecipitates prepared from HEK293T cells expressing the indicated proteins. The dashed lines indicate where different lanes of the same membrane were brought together. **b**, Native PAGE and autoradiography of a mixture of [^35^S]H_6_PEX7 and NtPEX13 that was subsequently incubated (chased) with a fivefold molar excess of GST-*Hs*PEX39 or GST-Ub (negative control). Aliquots before (*t*_0_) and during incubations were collected at the indicated time points. [^35^S]H_6_PEX7 in a mixture with GST-*Hs*PEX39 was also analysed. The in-gel positions of PEX7 alone, the complexes PEX7–*Hs*PEX39 (hashtag) and PEX7–NtPEX13 (at symbol) and other proteins are indicated. **c**, Native PAGE and autoradiography of mixtures of [^35^S]H_6_PEX7 and GST-*Hs*PEX39 or mixtures of [^35^S]H_6_PEX7, H_6_PHYH and GST-*Hs*PEX39 that were subsequently incubated (chased) with a 100-fold molar excess of either GST-*Hs*PEX39(ΔN) or GST-Ub (negative control). Aliquots before (*t*_0_) and during incubations were collected at the indicated time points. The in-gel positions of PEX7 alone and the complexes PEX7–*Hs*PEX39 (hashtag), PEX7–*Hs*PEX39(ΔN) (closed square bracket) and PEX7–PHYH–*Hs*PEX39 (ampersand) are indicated. Double bands of PEX7–*Hs*PEX39(ΔN) complexes were caused by co-migration with haemoglobin from the reticulocyte lysate. **d**, Model depicting how PEX39 facilitates the import of PTS2-containing proteins and the consequences of perturbations explored in this study. Proteins and their respective motifs or domains are indicated. Co-receptors (for example, PEX5, Pex18 and Pex21) are shown in blue. Protein cargo containing a PTS2 (denoted by an α) are also shown. The dashed lines highlight known protein–protein interactions^46,58–62^. 13, PEX13; 14/17, PEX14/PEX17; CTD, C-terminal domain; NTD, N-terminal domain; PEX7-BD, PEX7-binding domain; WxxxF, di-aromatic motif. Source data

The prediction that the (R/K)PWE motifs of PEX39 and the PEX13 N terminus bind the same PEX7 site (Fig. 7a) suggests that the interaction between these motifs with PEX7 is mutually exclusive. Consistent with this, *Hs*PEX39-FLAG-HA did not co-immunoprecipitate *Hs*PEX13 in HEK293T cells, whereas FLAG-HA-tagged PEX7, ACAA1 and PHYH did (Fig. 8a). Using our native PAGE assay to assess the time-dependent displacement of NtPEX13 from a PEX7–NtPEX13 complex by *Hs*PEX39 tagged with glutathione *S*-transferase (GST-*Hs*PEX39), we observed that NtPEX13 was readily replaced by *Hs*PEX39 even at the first time point (Fig. 8b, lane 3 versus 4), whereas in the absence of *Hs*PEX39, the PEX7–NtPEX13 complex was unchanged during the 20-min incubation (lane 3 versus 8), indicating mutually exclusive binding. These results suggest that PEX39 must dissociate from PEX7 to allow PEX7 to bind the N terminus of PEX13 at the peroxisome, a handover mechanism that would be facilitated if the PEX39–PEX7 interaction were labile. To address this, we assessed the time-dependent replacement of wild-type *Hs*PEX39 in PEX7–*Hs*PEX39 or PEX7–PHYH–*Hs*PEX39 complexes with an electrophoretically distinguishable variant, the aforementioned N-terminally truncated *Hs*PEX39(ΔN) (Fig. 8c). For the PEX7–*Hs*PEX39 complex, *Hs*PEX39 was completely replaced by *Hs*PEX39(ΔN) at the first time point (lane 2 versus 3), thus indicating that the interaction of *Hs*PEX39 with PEX7 is labile. For the PEX7–PHYH–*Hs*PEX39 complex, the switching of *Hs*PEX39 species was slower (lanes 8–12), consistent with the lower *K*_d,app_ for this complex.

## Discussion

**Extended Data Fig. 10:**
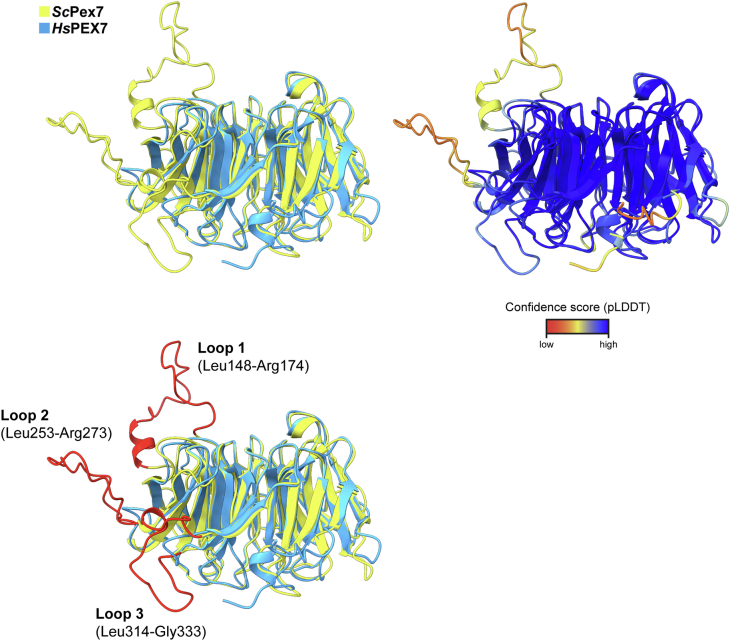
Comparison of structural predictions of yeast and human PEX7. Superimposition of AlphaFold structural models of human and yeast PEX7. Predicted models were directly received from the AlphaFold database (https://alphafold.ebi.ac.uk/) using accession codes AF-O00628-F1-v4 (*Hs*PEX7) and AF-P39108-F1-v4 (*Sc*Pex7) and superimposed using ChimeraX. AlphaFold confidence scores (that is, strength of structural predictions) are shown according to the predicted local distance difference test (pLDDT) (right). Extended loops of *Sc*Pex7 are highlighted in red and respective amino acids are labelled (bottom).

In this work (described previously in our preprint^49^), we identify and characterize human and yeast PEX39, an ancient and hitherto unknown component of the peroxisomal protein import machinery. In addition, we elucidate a new paradigm in peroxisomal import and biogenesis, namely the sequential engagement of PEX7 by two (R/K)PWE-containing peroxins, PEX39 and PEX13. Based on the results of this study, we propose a handover model for how PEX39 facilitates the import of PTS2-containing proteins, as well as the sequelae of PEX39 loss or overexpression (Fig. 8d). Under physiological conditions, PEX39 can directly bind PEX7 via the (R/K)PWE motif (step 1) and then stabilize the interaction between PEX7 and PTS2-containing protein cargo via an interacting N-terminal region (step 2). The fact that PEX39 can bind at two sites suggests that avidity would promote its retention within the complex between PEX39, PEX7 and PTS2-containing protein cargo. However, this avidity is lost upon co-receptor binding (for example, PEX5 or Pex18) as the co-receptor displaces the N-terminal region of PEX39 but independently stabilizes the interaction between PEX7 and PTS2-containing protein cargo (step 3). Because of the labile nature of the PEX39–PEX7 interaction via the (R/K)PWE motif, PEX39 can now be exchanged with the PEX13 N terminus, which also contains a KPWE motif (steps 4 and 5), thus handing PEX7 over from PEX39 to PEX13. PEX13 can now fully participate in the remaining steps required for PTS2-containing protein cargo translocation. A recent study investigating PTS2-containing protein cargo import in yeast supports our model^50^. In our work, in the setting of PEX39 loss or substitution of its KPWE motif, the import of PTS2-containing protein cargo is impaired due to destabilization of the interaction between PEX7 and PTS2-containing protein cargo, with an additional contribution in *S. cerevisiae* being the decreased abundance of Pex7, which suggests that *Sc*Pex39 stabilizes Pex7, potentially as a consequence of stabilizing the interaction between Pex7 and PTS2-containing protein cargo and promoting pre-import steps. The reason for *Hs*PEX39 loss not decreasing PEX7 levels might stem from different structural properties of the yeast and human orthologues, such as the presence of three loops in yeast Pex7 but not human PEX7 (Extended Data Fig. 10). In the setting of PEX39 overexpression or substitution of the PEX13 KPWE motif, PTS2-containing protein cargo import is also impaired because even though cargo-loaded, co-receptor-bound PEX7 can be recruited to PEX13, the PEX13 N terminus cannot bind PEX7 and facilitate the translocation of PTS2-containing protein cargo. The observation that PEX39 impairs import when depleted or overexpressed is surprising and is, to the best of our knowledge, unique among cytosolic peroxins; by extension, our results suggest that PEX39 levels must be well controlled to maximize the import of PTS2-containing proteins.

We envision multiple important areas for future investigation. Structural determination of the PEX39, PEX7 and PEX13 complexes investigated here will provide valuable insights. PEX7 belongs to the large family of WD40-domain proteins, which have three potential interaction sites: the top and bottom face and the circumference^51^. Indeed, the crystal structure of yeast Pex7 reveals that the PTS2 binds to the top face^44^, but an interactor for the bottom face has yet to be identified. Our work strongly suggests that the function of the bottom face is to allow PEX7 to interact with the (R/K)PWE motifs of PEX39 and PEX13 and thereby cycle between these two peroxins.

Additional investigation of the physiological consequences of PEX39 loss will also be of value. *HsPEX39*-KO cells have impaired import of PHYH, whose deficiency causes adult Refsum disease^52^. That *Hs*PEX39 loss only affects PHYH and not ACAA1 or AGPS is surprising and may suggest that the interaction between PEX7 and the PTS2 of PHYH benefits the most from the stabilizing effect of *Hs*PEX39. Given the role of PHYH in human physiology, it will be important to examine the consequences of PEX39 loss in mice and search for pathogenic human alleles.

In conclusion, almost all known peroxins were identified using forward genetic approaches in yeast^53^ and mammalian cells^54^. Although these approaches were extremely successful, they were not designed to identify genes with more subtle loss-of-function phenotypes^32^. Perhaps for this reason, after the identification of PEX26 more than 20 years ago^55^, no new human peroxin had been discovered. The work described here breaks this long pause by using functional proteomics instead of functional genomics, and demonstrates both the power of alternative approaches for studying peroxins and the abundance of mysteries still surrounding the import of peroxisomal proteins.

## Methods

### Experiments with yeast not related to fluorescence microscopy

#### Yeast culture conditions and metabolic labelling

Yeast cells were cultured at 30 °C and 160 r.p.m. in synthetic complete medium (pH 6.0) containing 0.17% yeast nitrogen base (lacking amino acids), 0.5% ammonium sulfate, 0.3% glucose, 0.002% histidine, methionine, adenine and uracil, 0.003% tryptophan, isoleucine and tyrosine, 0.005% arginine, lysine and phenylalanine, 0.01% leucine, 0.015% valine and 0.02% threonine (percentage values represent wt/vol in all instances) unless stated otherwise. Depending on the selection markers, uracil, histidine or both were omitted. To induce peroxisome proliferation, cells were grown in synthetic complete medium until an optical density at 600 nm (OD_600_) of 1.0–1.5 was achieved, shifted to YNO medium (synthetic complete medium containing 0.1% oleic acid (27728-1L-R; Sigma–Aldrich) and 0.05% Tween 40; percentage values represent vol/vol) and cultivated for a further 12–16 h or as indicated. For metabolic labelling using stable isotope labelling by amino acids in cell culture (SILAC)^63^, the medium contained stable isotope-coded heavy arginine (^13^C_6_/^15^N_4_; Arg10; CNLM-539-H; Eurisotop) and lysine (^13^C_6_/^15^N_2_; Lys8; CNLM-291-H; Eurisotop) instead of the unlabelled light counterparts (^12^C_6_/^14^N_*x*_; Arg0/Lys0).

For growth assays, strains were pre-cultured in synthetic complete medium containing 0.3% glucose. Cells were cultivated for 16 h and then shifted to either glucose (2%) or oleic acid media, adjusting the OD_600_ to approximately 0.1. Aliquots of the cultures were taken at distinct time points, as indicated, and the OD_600_ was determined. Cells grown in YNO medium were washed twice with ultrapure water and resuspended in an appropriate volume of ultrapure water before the OD_600_ was measured.

Yeast cells used for transformation (that is, in complementation studies and sedimentation assays) were grown overnight in YPD medium (1% yeast extract, 2% peptone, 2% glucose, 0.002% uracil and 0.002% adenine; percentage values represent wt/vol in all instances) at 30 °C and 160 r.p.m., diluted with YPD medium to an OD_600_ of 0.1 in 20 ml and incubated for a further 4–5 h at 30 °C and 160 r.p.m. until an OD_600_ of 0.4–0.7 was reached.

#### Yeast strains and plasmids

The following plasmids were used:

1. pRS313 PEX39Pro-*Sc*Pex39-PEX39Term (P797; B.W. laboratory)
2. pRS313 TEF2Pro-*Sc*Pex39-ADH1Term (P798; B.W. laboratory)
3. pRS313 TEF2Pro-Pex7-ADH1Term (P799; B.W. laboratory)
4. pRS313 PEX39Pro-*Sc*Pex39(RPWE/AAAA mutant)-PEX39Term (P815; B.W. laboratory)
5. pRS313 PEX13Pro-*Sc*Pex13-PEX13Term (P801; B.W. laboratory)
6. pRS313 PEX13Pro-*Sc*Pex13(KPWE/AAAA mutant)-PEX13Term (P807; B.W. laboratory)
7. pRS313 PEX39Pro-*Sc*Pex39(aa 40–132)-PEX39Term (P820; B.W. laboratory)

The oligonucleotides used to generate yeast strains and plasmids are listed in Supplementary Table 1. The yeast strains are listed in Supplementary Table 4. All modified plasmids and yeast strains were confirmed by sequencing of the respective region of interest. Genomic manipulation of yeast cells was performed by homologous recombination following transformation of the cells with the respective PCR product. For genomic C-terminal tagging of Pex18 with the TEV protease cleavage site and protein A (that is, the TPA tag), the DNA sequence coding for the TPA tag followed by the selection marker kanMX4 was inserted at the 3′ end of the *pex18* gene^64^. To generate the *Scpex39* and *pex7* deletion strain, the respective gene was replaced with the URA3 marker cassette^65^.

To generate plasmid P797, the *Scpex39* open reading frame (ORF) plus promoter and terminator regions were amplified from yeast genomic DNA using the primer pair SF1/SF2 and cloned into pRS313 (ref. ^66^) using the SalI restriction enzyme. P798 was generated by inserting the ORF of *Scpex39* (amplified using the primers SF3 and SF4) into a modified pRS313 backbone comprising the TEF2 promoter and ADH1 terminator. To generate P799, the *pex7* ORF was amplified from genomic DNA using the primers SF10 and SF11 and inserted via the restriction enzymes XbaI and AscI into P798. To generate P815, the amino acids of the RPWE motif of *Sc*Pex39 in P797 were substituted with AAAA by site-directed, ligase-independent PCR-mediated mutagenesis^67^, using two primer pairs (O2145/O2143 and O2144/O2142) to amplify the P797 plasmid DNA by PCR. P801 and P807 were generated as described for P797 and P815 using the following primer pairs: SF5/SF6 (introducing a XhoI restriction site), as well as O2181/O2179 and O2180/O2178. P820 was generated by a deletion PCR using the primers SF12 and SF13 on P797.

#### Transformation of yeast cells

Yeast cells were grown in YPD medium as described above in the section ‘Yeast culture conditions and metabolic labelling’, harvested by centrifugation (5 min; 500*g*; room temperature) and washed first with 20 ml ultrapure water and then with 10 ml SORB (100 mM lithium acetate, 10 mM Tris-HCl (pH 8.0) and 1 M sorbitol). The cell pellet was resuspended in 360 μl SORB, mixed with 40 μl denatured salmon sperm and directly used for transformation, applying the heat-shock method^64^.

#### Preparation of yeast whole-cell lysates using trichloroacetic acid precipitation

To generate whole-cell lysates from oleate-induced yeast cells, 30 mg (wet weight) freshly harvested cells were precipitated using 12% (vol/vol) trichloroacetic acid (TCA) in 36 mM potassium phosphate buffer (pH 7.4) for 16 h at −80 °C. Precipitated proteins were thawed on ice, pelleted by centrifugation (10 min; 15,871*g*; 4 °C) and washed twice with 80% (vol/vol) ice-cold acetone. Residual acetone was removed by evaporation and dried proteins were resuspended in 80 µl 0.1 M NaOH/1% (wt/vol) sodium dodecyl sulfate (SDS). For sodium dodecyl sulfate polyacrylamide gel electrophoresis (SDS-PAGE), 5× SDS buffer was added and samples were boiled for 8 min at 95 °C.

#### Affinity purification of Pex18 complexes from yeast

Native Pex18 complexes were affinity purified using Pex18-TPA-expressing yeast cells grown under peroxisome-proliferating conditions, as described before but with minor modifications^16,68^. Isogenic cells expressing the native, non-tagged version of Pex18 grown under the same conditions served as a control. Cells were lysed using glass beads (G9268; Sigma–Aldrich) and an MM 400 mixer mill (Retsch) at 20 Hz and 4 °C for 8 min. All subsequent steps were performed as described previously^68^ for soluble complexes and without immobilization of the TEV protease.

#### Preparation of yeast cell lysates used for SILAC mass spectrometry experiments

Oleate-induced *Scpex39*Δ and isogenic wild-type cells, labelled with light or heavy arginine and lysine, were harvested by centrifugation (2 min; 1,600*g*; 4 °C) and washed twice with ultrapure water. Cells were resuspended in 500 μl lysis buffer (8 M urea, 75 mM NaCl, 50 mM Tris-HCl and 1 mM EDTA (pH 8.0)), and equal amounts of differentially labelled *Scpex39*Δ and wild-type cells were mixed based on the cell wet weight. Cells were lysed using glass beads (300 mg) and a Minilys homogenizer (Bertin Technologies), applying two cycles of 4 min at 5,000 r.p.m. with at least 2 min of cooling on ice between cycles. Glass beads and cell debris were removed by centrifugation (5 min; 15,000*g*; 4 °C). The protein concentration of the cell lysates was adjusted to 1 μg μl^−1^ using urea buffer (8 M urea in 50 mM ammonium bicarbonate). The experiment was performed in four independent replicates with a light/heavy label switch.

#### Tryptic in-gel digestion

Affinity-purified Pex18 protein complexes and proteins of the control purifications were acetone precipitated and resuspended in 0.1 M NaOH/1% (wt/vol) SDS. Proteins were separated by SDS-PAGE, lanes were cut into 11 slices and proteins were processed for liquid chromatography–mass spectrometry (LC–MS) analysis, as described previously^69^ with slight modifications. Tryptic peptide mixtures of each sample were combined, dried in vacuo, desalted using StageTips (2215; 3M Empore)^70^ and dried again in vacuo.

#### Proteolytic in-solution digestion

Proteins (300 μg) of yeast whole-cell lysates prepared from SILAC-labelled *Scpex39*Δ and wild-type cells were digested in solution using Lys-C (125-05061; FUJIFILM Wako) and trypsin (V5111; Promega) as described before^69^. Peptides were subsequently desalted using C18-SD 7 mm/3 ml extraction disc cartridges (4215SD; 3M Empore) as described previously^71^, dried in vacuo and further fractionated by high-pH reversed-phase liquid chromatography.

#### High-pH reversed-phase liquid chromatography

Peptide fractionation by high-pH reversed-phase liquid chromatography^72^ was performed essentially as described before^71^. Dried peptides were reconstituted in 200 μl 1% (vol/vol) acetonitrile (ACN)/10 mM NH_4_OH (pH 10) by sonication, followed by centrifugation (5 min; 12,000*g*; room temperature) to remove insoluble material, then further purification using a 0.2-μm polytetrafluoroethylene membrane syringe filter (AF0-3202-12; Phenomenex). Peptides were separated using an UltiMate 3000 HPLC system (Thermo Fisher Scientific) operated with an NX 3-μm Gemini C18 column (150 mm × 2 mm inner diameter; particle size of 3 μm; pore size of 110 Å; 00F-4453-B0; Phenomenex) at 40 °C and a flow rate of 200 μl min^−1^. Peptide elution was performed using a binary solvent system comprising 10 mM NH_4_OH (solvent A1) and 90% (vol/vol) ACN/10 mM NH_4_OH (solvent B1). Peptides were loaded onto the column at 1% solvent B1 for 5 min and separated by increasing B1 from 1–40% in 37 min and from 40–78% in 3 min, followed by 5 min at 78% B1, before the column was re-equilibrated with 1% solvent B1. Starting at 1.5 min until 65.5 min, 45-s fractions were collected in a concatenated manner, resulting in a total of eight fractions per sample. Peptides were dried in vacuo, desalted using StageTips^70^ and dried again.

#### LC–MS analysis

Before LC–MS analysis, dried peptides were resuspended in 0.1% (vol/vol) TFA and insoluble material was removed by centrifugation (12,000*g*; 5 min; room temperature). Peptides were analysed by nano-HPLC-ESI-MS/MS using a Q Exactive Plus mass spectrometer (Thermo Fisher Scientific) connected to an UltiMate 3000 RSLCnano HPLC system (Thermo Fisher Scientific). The RSLC system was operated at 40 °C with C18 trapping columns (μPAC; 10 mm × 2 mm inner diameter; PharmaFluidics) at a flow rate of 10 μl min^−1^ and a C18 endcapped analytical column (μPAC; 500 mm × 0.3 mm; PharmaFluidics) at a flow rate of 300 nl min^−1^. A binary solvent system comprising 0.1% (vol/vol) formic acid (solvent A2) and 86% (vol/vol) ACN/0.1% (vol/vol) formic acid (solvent B2) was employed for peptide separation. For the analysis of peptides from the Pex18-TPA affinity purification experiments, peptides were loaded onto the pre-column, washed and pre-concentrated for 3 min at 1% solvent B2 and eluted by applying the following gradient: 1–4% B2 in 2 min, 4–25% B2 in 20 min, 25–44% B2 in 11 min, 44–90% B2 in 2 min and 4 min at 90% B2. The same solvent system was used for the analysis of samples from the *Scpex39*Δ-versus-wild-type SILAC experiments. Peptides equivalent to 1 μg protein were loaded, washed and pre-concentrated for 5 min using 1% solvent B2. For peptide elution, a gradient ranging from 1–5% B2 in 3 min, 5–22% B2 in 103 min, 22–42% B2 in 50 min and 42–80% B2 in 5 min was applied.

Mass spectrometric data were acquired in data-dependent acquisition mode. Mass spectrometry spectra were recorded in a mass-to-charge (*m/z*) range of 375–1,700 with a resolution of 70,000 (at *m/z* 200). The automatic gain control was set to 3 × 10^6^ and the maximum injection time was set to 60 ms. The 12 most intense precursor ions (*z* ≥ +2) were selected for fragmentation by higher-energy collisional dissociation, applying a normalized collision energy of 28%, a resolution of 35,000, an automatic gain control of 10^5^, a maximum injection time of 120 ms and a dynamic exclusion time of 45 s.

#### Mass spectrometry data processing and analysis

Proteins present in Pex18-TPA pulldowns were identified using the Andromeda search engine^73^ implemented in MaxQuant (version 2.0.1.0)^74^ through comparison with the UniProt reference proteome of *S. cerevisiae* (including isoforms; released February 2024; 6,079 entries), extended by the sequences of the TEV protease and immunoglobulins used in this study. Trypsin/P (no cleavage before proline) was specified as digestion enzyme, oxidation of methionine and N-terminal acetylation as variable modifications, and carbamidomethylation of cysteine as a fixed modification. Identifications were transferred between samples using match between runs with standard settings. Protein mass spectrometry intensities were normalized to intensity-based absolute quantification (iBAQ) values. The minimum number of unique peptides per protein group was set to 1.

The autoprot Python module (version 0.2)^75^ was used for data analysis and processing. Decoy and contaminant entries, as well as protein groups without quantitative information, were removed. Moreover, protein groups with a sequence coverage of <10% were removed. For protein groups from each gel slice of Pex18-TPA-expressing or control cells, the median log_10_[iBAQ value] was calculated. The mean of medians between Pex18-TPA and control cells was calculated for each corresponding gel slice pair and subtracted from all iBAQ values of both slices. The median-corrected iBAQ values were exponential transformed to gain non-log values, and iBAQ values for protein groups of all slices of a replicate were summed. Protein groups with fewer than two valid values in the Pex18-TPA replicates were removed. Missing intensity values were imputed by drawing random values from a distribution matching the intensity distribution of the existing values but shifted by 1.8 standard deviations and scaled to 30% width. The statistical significance of differences in protein group intensity was computed using the rank-sum test implemented in the R package RankProd^76^ and the results were visualized in autoprot. The data analysis was documented using Jupyter Notebook and is available from https://github.com/ag-warscheid/Pex39_Manuscript and ref. ^77^.

For protein identification and SILAC-based relative quantification in the *Scpex39*Δ-versus-wild-type experiments, MaxQuant (version 2.4.4.0) was employed. Tandem mass spectrometry data were searched against the *S. cerevisiae* reference proteome provided by the *Saccharomyces* Genome Database (http://sgd-archive.yeastgenome.org/sequence/S288C_reference/orf_protein; downloaded August 2023) using default parameters with the following exceptions: Arg10/Lys8 was selected as the heavy label; the multiplicity was set to 2; trypsin and Lys-C were set as proteolytic enzymes, allowing a maximum of three missed cleavages; and the options match between runs and requantify were enabled. A false discovery rate of 1% was applied at the peptide and protein levels. Protein quantification was based on unique peptides and at least one ratio count. Autoprot was used for further data analysis and visualization, considering only proteins quantified in at least three out of four replicates, except for *Sc*Pex39, which was quantified in only one replicate. To include *Sc*Pex39, which is only present in wild-type cells, in our data analysis and visualization, missing *Sc*Pex39 ratios were imputed by randomly drawing values deviating ±0.05 from the single log_2_-transformed value reported by MaxQuant. The normalized protein abundance ratios calculated by MaxQuant were log_2_ transformed, followed by sequential imputation of missing values and cyclic loess normalization^78^. To identify proteins with differences in protein abundance between wild-type and *Scpex39*Δ cells, the linear models for microarray data (limma) approach was employed. This method is a moderated two-sided *t*-test that adjusts a protein’s variance in ratios between replicates towards the average ratio variance of the entire dataset^79,80^. *P* values were corrected for multiple testing according to Benjamini and Hochberg^81^. The data analysis was documented using Jupyter Notebook and is available from https://github.com/ag-warscheid/Pex39_Manuscript and ref. ^77^.

#### STRING network analysis

STRING network analysis was performed using the STRING web application (version 12; https://string-db.org/)^82^. A multiple-protein search was performed using gene names as identifiers and setting ‘*Saccharomyces cerevisiae*’ as the organism. Under ‘active interaction sources’ in the basic setting, ‘Experiments’ and ‘Databases’ were selected as sources and the ‘minimum required interaction score’ was set to 0.4 (medium confidence). Interactions without experimental or biochemical data or those based on putative homologues in other organisms were filtered out.

#### Gene Ontology term enrichment analysis

Gene Ontology term enrichment analysis of proteins exhibiting a minimum *Q* value of <0.05 for the Pex18-TPA complexes dataset (related to Fig. 1c) were performed using the g:Profiler application (https://biit.cs.ut.ee/gprofiler/gost)^83^. False discovery rate *P* values were corrected for multiple testing using the Benjamini–Hochberg method. Gene Ontology terms with corrected *P* values of <0.05 in the Pex18-TPA complexes dataset were considered enriched.

#### Cellular fractionation by sedimentation assay

Freshly harvested oleate-induced yeast cells (5 g per experiment) were resuspended in 25 ml dithiothreitol (DTT) buffer (10 mM DTT in 100 mM Tris) and incubated for 20 min at 37 °C and 60 r.p.m. in a 250-ml Erlenmeyer flask. Cells were harvested by centrifugation (10 min; 600*g*; room temperature), resuspended in fresh DTT buffer and incubated again as described above. Cells were harvested and washed three times with 20 ml 1.2 M sorbitol pre-heated to 37 °C. To prepare spheroplasts, cells were then resuspended in 35 ml 1.2 M sorbitol buffer (1.2 M sorbitol in 20 mM potassium phosphate (pH 7.4), pre-heated to 37 °C) containing 1,000 U lyticase (L4025; Merck/Sigma–Aldrich) per gram of cell wet weight and incubated for 30 min at 37 °C and 60 r.p.m. The digestion was stopped by incubation on ice for 10 min. Spheroplasts were washed three times with 15 ml pre-cooled 1.2 M sorbitol and collected by centrifugation (10 min; 600*g*; 4 °C) after each washing step. Spheroplasts were resuspended in 5 ml homogenization buffer (5 mM 2-(*N*-morpholino)ethanesulfonic acid; 0.5 mM ethylenediaminetetraacetic acid (EDTA), 1 mM KCl and 0.6 M sorbitol (pH 6.0)) containing protease and phosphatase inhibitors^16,68^ using a Dounce homogenizer operated at 2× 100 r.p.m. (2 min each), 3× 300 r.p.m. (1 min each), 3× 500 r.p.m. (1 min each) and 3× 800 r.p.m. (1 min each). Cell debris and nuclei were removed by centrifugation (2× 10 min; 600*g*; 4 °C). Protein concentrations of the resulting PNSs obtained from different strains within given experiments were adjusted using homogenization buffer. The PNS (1 ml) was loaded on top of a 200-μl sucrose cushion (0.5 M sucrose in homogenization buffer) and separated into an organellar pellet and a cytosolic fraction by centrifugation (20 min; 25,000*g*; 4 °C). The organellar pellet was resuspended in 1 ml homogenization buffer. Equal volumes of PNS, cytosolic fraction and organellar pellet were analysed by SDS-PAGE and semi-dry immunoblotting.

#### Carbonate and salt extraction using organellar fractions

Sodium carbonate and salt extraction experiments were performed as described previously^84^ with slight modifications. In brief, for carbonate extraction, organellar pellets were resuspended in 300 µl homogenization buffer (as described for cellular fractionation), whereas for salt extraction experiments organellar pellets were resuspended in low-salt buffer (5 mM Tris-HCl (pH 7.6), 1 mM EDTA and 1 mM DTT). Equal aliquots were taken and mixed with SDS buffer, which served as the total (T) sample. For carbonate extraction, 100 µl organelle suspension was mixed with 100 µl 0.2 M Na_2_CO_3_ (pH 11.5), incubated for 30 min on ice and subsequently pelleted by centrifugation (30 min; 100,000*g*; 4 °C). Supernatants were taken and proteins precipitated using TCA, whereas the membrane pellet was directly resuspended in 40 µl homogenization buffer and subsequently mixed with 10 µl 5× SDS buffer.

For salt extraction, 200 µl organelles in low-salt buffer were incubated for 30 min on ice and subsequently pelleted by centrifugation (30 min; 100,000*g*; 4 °C). Soluble proteins in the supernatant were precipitated as described for the sodium carbonate extraction and insoluble proteins in the pellet fraction were resuspended using 200 µl high-salt buffer (25 mM Tris-HCl (pH 7.6), 0.5 M KCl, 1 mM EDTA and 1 mM DTT). Next, 100 µl of this fraction was removed and the proteins were precipitated as described above and served as sample P of the low-salt treatment. The residual 100 µl of the fraction was incubated for 30 min on ice and subsequently pelleted by centrifugation (30 min; 100,000*g*; 4 °C). The supernatant and pellet fractions resulting from this centrifugation are referred to as S and P of the high-salt treatment. Before immunoblot analysis, proteins in the supernatant were precipitated using TCA and the membrane pellet was directly resuspended in 40 µl homogenization buffer and subsequently mixed with 10 µl 5× SDS buffer.

#### SDS-PAGE and immunoblotting

SDS-PAGE and semi-dry immunoblotting were performed following standard protocols unless otherwise specified. The antibodies used for immunoblotting were as follows: goat polyclonal anti-Cta1 (*S. cerevisiae*; 1/20,000 dilution)^85^; rabbit polyclonal anti-Pex7 (*S. cerevisiae*; 1/2,000 dilution)^46^; rabbit polyclonal anti-Pex3 (*S. cerevisiae*; 1/3,000 dilution; from the Erdmann group at Ruhr University Bochum); rabbit polyclonal anti-Pex18 (*S. cerevisiae*; 1/10,000 dilution)^86^; rabbit polyclonal anti-Pot1 (*S. cerevisiae*; 1/10,000 dilution)^87^; rabbit polyclonal anti-Gpd1 (*S. cerevisiae*; 1/10,000 dilution)^88^; rabbit polyclonal anti-protein A (*S. aureus*; P3775; Sigma–Aldrich; 1/10,000 dilution); goat HRP-conjugated polyclonal anti-rabbit (A0545; Sigma–Aldrich; 1/10,000 dilution); rabbit HRP-conjugated polyclonal anti-goat (A8919; Sigma–Aldrich; 1/10,000 dilution); rabbit HRP-conjugated polyclonal anti-mouse (A9044 Sigma–Aldrich; 1/10,000 dilution); mouse monoclonal anti-Por1 (*S. cerevisiae*; 459500; Invitrogen; 1/5,000 dilution); mouse monoclonal anti-Pgk1 (*S. cerevisiae*; 459250; Invitrogen; 1/1,000 dilution); and mouse monoclonal anti-Dpm1 (*S. cerevisiae*; A-6429 Invitrogen; 1/2,000 dilution).

#### Quantification of immunoblots

Immunoblot signals were quantified using the software ImageJ (version 1.54d)^89^. Signal intensities were corrected for background intensities.

### Fluorescence microscopy of yeast

#### Yeast growth media

Yeast were grown in synthetic media containing 6.7 g l^−1^ yeast nitrogen base with ammonium sulfate (1545; Conda Pronadisa) and 2% glucose (SD media) or 0.2% oleic acid (O1008; Sigma–Aldrich) + 0.1% Tween 80 (P4780; Sigma–Aldrich) with a complete amino acid mix (oMM composition)^90^. When Geneticin antibiotic was used, the media contained 0.17 g l^−1^ yeast nitrogen base without ammonium sulfate (1553; Conda Pronadisa) and 1 g l^−1^ monosodium glutamic acid (G1626; Sigma–Aldrich). The strains were selected using a dropout mix (the same composition as for the SD media described above, but without the specific amino acid for selection) or with antibiotics using the following concentrations: 500 mg l^−1^ Geneticin (G4185; Formedium) and 200 mg l^−1^ Nourseothricin (also known as ClonNAT; AB-102-25G; WERNER BioAgents).

#### Yeast strain construction

Genetic manipulations were performed using PCR-mediated homologous recombination with the lithium acetate method^91^. The correct tagging or deletion was verified in all strains by PCR. The primers in this study were either designed using the web tool Primers-4-Yeast^92^ or manually constructed in the case of the deletion of *Scpex39*. All of the relevant primers are listed in Supplementary Table 1. Supplementary Table 4 contains descriptions of the yeast strains.

The plasmids used included:

1. pFA6a-NAT-MX6 (a KO-mediated plasmid with a Nat cassette with a TEF2 promoter and terminator; pMS49; M.S. group)
2. mNG C-terminal tagging plasmid (plasmid for C-terminal tagging with mNeonGreen; pMS1190; M.S. group)

#### PCR validation of genomic transformations

Freshly grown yeast cells were picked from agar plates and suspended in PCR tubes containing 50 μl 20 mM NaOH with 0.1 mg ml^−1^ RNaseA. The suspension was then boiled at 100 °C for 20 min in a PCR machine and spun down in a microcentrifuge for 3 min. The supernatant was used as template DNA for a PCR reaction (2 μl), alongside 2× GoTaq Green Master Mix (5 μl; M7122; Promega), forward primer (0.2 μl from 10 μM concentration), reverse primer (0.2 μl from 10 μM concentration) and double-distilled water up to a final volume of 10 μl. The DNA was amplified using the following thermocycling steps: 98 °C for 3 min; 35 cycles of 98 °C for 60 s, 55 °C for 90 s, 72 °C for 30 s and 72 °C for 60 s. The resulting PCR product was then run on a 1% agarose gel and examined for the correct size of the amplicon.

#### Imaging of yeast strains

Yeast strains were grown overnight in an SD-based medium supplemented with amino acids in 96-well polystyrene plates and then transferred to oleic acid for 4 h (for the experiment shown in Extended Data Fig. 1e) or 8 h (for the experiment shown in Fig. 2d). The strains were then manually transferred into 384-well glass-bottom microscope plates (MGB101-1-2-LG-L; Matrical Bioscience) coated with concanavalin A (C2010; Sigma–Aldrich). After 20 min, the wells were washed twice with DDW to remove non-adherent cells and obtain a cell monolayer. Imaging was performed in DDW. Images were taken using the Olympus IXplore SpinSR system, comprising an Olympus IX83 inverted microscope scanning unit (SCU-W1) operated by ScanR (version 3.2.0). When high-resolution images were taken (that is, Fig. 2d), a high-resolution spinning disk module (a Yokogawa CSU-W1 SoRa confocal scanner with double microlenses and 50-μm pinholes) was used. Cells were imaged using an ×60 oil lens (numerical aperture = 1.42) and a Hamamatsu ORCA-Flash4.0 camera. Images were recorded in two channels: mNeonGreen (excitation wavelength = 488 nm) and mScarlet (excitation wavelength = 561 nm). For all micrographs, a single, representative focal plane was imaged and shown.

### Experiments with human cells, except those utilizing HeLa cells

#### Human cell lines

For routine culturing and experiments, all human cell lines were grown at 37 °C under 5% CO_2_ and in media supplemented with 10% foetal bovine serum (FBS; 100-106; GeminiBio). HEK293T (CRL-3216; ATCC) and HCT116 cells (CCL-247; ATCC) were grown in Dulbecco′s modified Eagle′s medium with high glucose (D5796; Sigma–Aldrich) supplemented with 1 mM sodium pyruvate (S8636; Sigma–Aldrich). CAKI-2 cells (a gift from G. Wyant and W. Kaelin Jr) were grown in McCoy’s 5A (Modified) Medium (16600-082; Gibco) and NCI-H1792 cells (CRL-5895; ATCC) were grown in RPMI-1640 medium (R8758; Sigma–Aldrich). All complete media was passed through a 0.22-μm filter before use. Cells were routinely tested for *Mycoplasma*.

#### Reagents and antibodies

Materials were obtained from the indicated sources: puromycin (ant-pr-1; InvivoGen); doxycycline hyclate (446060050; Thermo Scientific Chemicals); antibodies to ACAA1 (HPA006764; Sigma–Aldrich), AGPS (HPA030211; Sigma–Aldrich), C6ORF226 (HPA045350; Sigma–Aldrich), PEX5 (HPA039260; Sigma–Aldrich), *Hs*PEX13 (HPA032142; Sigma–Aldrich), ACTB (sc-69879; Santa Cruz Biotechnology), PEX7 (20614-1-AP; Proteintech), PHYH (12858-1-AP; Proteintech), SCP2 (23006-1-AP; Proteintech), CANX (2433; Cell Signaling Technology), citrate synthase (14309; Cell Signaling Technology), GAPDH (2118; Cell Signaling Technology), HA (3724; Cell Signaling Technology), Histone H3 (3638; Cell Signaling Technology) and RPS6KB1 (2708; Cell Signaling Technology); HRP-conjugated anti-rabbit secondary antibody (7074; Cell Signaling Technology); and HRP-conjugated anti-mouse secondary antibody for IP (ab131368; Abcam).

#### Genetic modification of human cells

pHAGE vectors encoding the following were obtained as follows. C-terminally FLAG-HA-tagged human ACAA1, PHYH, PEX7 and *Hs*PEX39 were obtained from the Human ORFeome collection (version 8). C-terminally FLAG-HA-tagged human GAPDH, the KPWE variants of *Hs*PEX39 and the E77A variant of human PEX7 were generated by cloning gblocks (Integrated DNA Technologies) containing the corresponding complementary DNA (cDNA) into a pHAGE vector containing a C-terminal FLAG-HA tag (linearized with BsrGI-HF (R3575; New England Biolabs (NEB)) via NEBuilder HiFi DNA assembly (E2621; NEB). N-terminally FLAG-HA-tagged eGFP was a gift from A. Ordureau. Human GAPDH, wild-type *Hs*PEX39 and *Hs*PEX39 with a substituted KPWE motif were generated by cloning gblocks containing the corresponding cDNA into a pHAGE vector (linearized with BsrGI-HF) via NEBuilder HiFi DNA assembly. Three independent sgRNAs against *HsPEX39* (sg*HsPEX39*_1–3) and sgRNAs against *PEX7* (sg*PEX7*) and *AAVS1* (sg*AAVS1*) were cloned into a lentiCRISPR v2 vector (TLCV2; doxycycline-inducible Cas9; 87360; Addgene; ref. ^93^) linearized with BsmBI-v2 (R0739; NEB) by ligation of annealed oligonucleotides (Integrated DNA Technologies) with Quick Ligase (M2200; NEB). The oligonucleotide sequences used for the sgRNAs are provided in Supplementary Table 1.

For the production of lentivirus, each of these constructs was transfected with the lentiviral packaging vectors psPAX2 and pMD2.G into HEK293T cells using PolyJet DNA transfection reagent (SL100688; SignaGen Laboratories). The media was changed 24 h after transfection. The virus-containing supernatant was collected 48 h after transfection and passed through a 0.45-μm filter to eliminate cells.

The desired cell lines were then infected with lentivirus added to complete culture media containing 8 μg ml^−1^ polybrene (TR-1003; Sigma–Aldrich). At 24 h after infection, the media was changed. At 48 h after infection, cells were selected with puromycin (1 μg ml^−1^ for all cell lines except CAKI-2, for which 2 μg ml^−1^ puromycin was used) until an uninfected control was completely dead, at which point infected cells were freed from selection.

CAKI-2 and NCI-H1792 cells transduced with lentivirus harbouring TLCV2 with sg*AAVS1* (negative control) or sg*PEX7* or the three independent sgRNAs sg*HsPEX39*_1–3 were used for our lentiviral, doxycycline-inducible CRISPR–Cas9 system. After puromycin selection, these cells were treated with 1 μg ml^−1^ doxycycline hyclate, which stimulates the production of Cas9 and eGFP. Cells were treated with doxycycline hyclate for 9 d to achieve substantial depletion of PEX7 and *Hs*PEX39 across the population before the drug was removed.

To generate *HsPEX39* KOs with matched controls, we took CAKI-2 and NCI-H1792 cells with sg*AAVS1* or sg*HsPEX39*_1 on the ninth day of treatment with doxycycline hyclate and sorted single-cell clones into wells with conditioned media using fluorescence-activated cell sorting (FACS) based on eGFP signal. The composition of this conditioned media was a 1:1 mixture of fresh and spent complete culture media with no doxycycline hyclate that was supplemented with 1% penicillin–streptomycin (P0781; Sigma–Aldrich) and additional FBS to achieve a final concentration of 20%. Single-cell clones were expanded for 2–3 weeks and visible colonies for cells with sg*HsPEX39*_1 were screened by immunoblotting for the absence of *Hs*PEX39. Equal amounts of cells originating from eight *HsPEX39*-KO clones were then pooled together to make the final KO line (that is, diversifying the population of cells). Equal amounts of cells originating from eight clones with sg*AAVS1* were also pooled together to make the final matched controls. CAKI-2 and NCI-H1792 cells were chosen for their excellent performance in our workflow for isolating and expanding single-cell clones, and for their greater expression of *HsPEX39* and/or *PHYH*, *ACAA1* and *AGPS* compared with HCT116 and HEK293T cells.

To generate *PEX7* knockdowns with matched controls, we took CAKI-2 cells with sg*PEX7* or sg*AAVS1* (control) on the ninth day of treatment with doxycycline hyclate and sorted Cas9-expressing cells into complete culture media supplemented with 1% penicillin–streptomycin using FACS based on eGFP signal.

#### Anti-FLAG immunoprecipitation using human cells

The immunoprecipitation workflow was done at 4 °C or on ice unless otherwise indicated and low-retention microcentrifuge tubes (3448; Thermo Fisher Scientific) were used. Fully confluent 10-cm dishes of HCT116 or HEK293T cells stably expressing FLAG-HA-tagged proteins were washed once with ice-cold Dulbecco’s phosphate-buffered saline without calcium and magnesium (pH 7.4) (DPBS; 21-031-CM; Corning) and immediately scraped into 500 μl lysis buffer (50 mM Tris-HCl (pH 7.5), 150 mM NaCl, 0.5% (vol/vol) NP-40 alternative (492016; Millipore), one tablet of cOmplete Protease Inhibitor Cocktail (04693116001; Roche) per 25 ml lysis buffer, one tablet of PhosSTOP (04906837001; Roche) per 10 ml lysis buffer and 1 mM DTT (43816; Sigma–Aldrich)). Lysates were incubated with rotation for 30 min at 4 °C before being cleared by centrifugation at 17,000*g* and 4 °C for 10 min. Protein quantification was performed on the clarified lysate using the DC protein assay (500-0116; Bio-Rad). Anti-FLAG M2 magnetic beads (M8823; Millipore) were washed three times with a 10× packed bead volume of bead wash buffer (50 mM Tris-HCl (pH 7.5), 150 mM NaCl and 0.5% (vol/vol) NP-40 alternative). A DynaMag-2 magnet (12321D; Thermo Fisher Scientific) was used for collecting beads. Clarified lysate containing ~3 mg protein was then added to a 10-μl packed bead volume of the anti-FLAG beads for each immunoprecipitation, with the total solution volume brought up to 500 μl by adding lysis buffer. Samples were incubated with rotation for 4 h at 4 °C. Following immunoprecipitation, beads were washed three times with a 20× packed bead volume of lysis buffer containing 400 mM NaCl. Proteins were eluted from beads using 1× Laemmli sample buffer (1610747; Bio-Rad) containing 2.5% (vol/vol) 2-mercaptoethanol (M3148; Sigma–Aldrich) and then heated at 72 °C for 10 min for SDS-PAGE. For the preparation of cell lysate samples for SDS-PAGE, clarified lysates were adjusted to have equal protein concentrations across samples and then mixed with 4× Laemmli sample buffer containing 10% (vol/vol) 2-mercaptoethanol to achieve a final 1× concentration and then heated at 72 °C for 10 min.

#### Fractionation of human cells

Cellular fractionation was performed using the Cell Fractionation Kit (9038; Cell Signaling Technology), generally according to the manufacturer’s instructions. Wide Bore P1000 tips were used to manipulate solutions containing cells and organelles to minimize damage. All steps were performed on ice or at 4 °C, except those involving the kit’s nuclear isolation buffer, which were performed at room temperature to avoid precipitation. One-quarter of a tablet of cOmplete Protease Inhibitor Cocktail per 5 ml of the kit’s cytosolic isolation buffer, organellar isolation buffer and nuclear isolation buffer was used instead of the kit’s protease inhibitor cocktail.

Cellular fractions from HCT116 cells were generated by first preparing a suspension of 5 million live HCT116 cells in 500 μl ice-cold DPBS. From this, 100 μl of the cell suspension (that is, whole cells) was mixed with 4× Laemmli sample buffer containing 10% (vol/vol) 2-mercaptoethanol to achieve a final 1× concentration. The sample was then sonicated and heated at 95 °C for 5 min and centrifuged at 15,000*g* for 3 min at room temperature; then the supernatant was taken for SDS-PAGE. The remaining 400 μl of the cell suspension in DPBS was centrifuged at 500*g* for 5 min at 4 °C and the supernatant was discarded. The pellet was resuspended in 400 μl of the cytosolic isolation buffer, vortexed for 5 s, left on ice for 5 min and then centrifuged at 500*g* for 5 min at 4 °C. 100 μl of the supernatant (that is, cytosolic fraction) was mixed with 4× Laemmli sample buffer containing 10% (vol/vol) 2-mercaptoethanol to achieve a final 1× concentration, then heated at 95 °C for 5 min and centrifuged at 15,000*g* for 3 min at room temperature before the supernatant was taken for SDS-PAGE. The remainder of the cytosolic fraction was aspirated off and the pellet was resuspended in 400 μl organellar isolation buffer, vortexed for 15 s, left on ice for 5 min and then centrifuged at 8,000*g* for 5 min at 4 °C. 100 μl of the supernatant (that is, organellar fraction) was mixed with 4× Laemmli sample buffer containing 10% (vol/vol) 2-mercaptoethanol to achieve a final 1× concentration, then heated at 95 °C for 5 min and centrifuged at 15,000*g* for 3 min at room temperature before the supernatant was taken for SDS-PAGE. After aspiration of the remaining organellar fraction, the pellet was resuspended in 400 μl nuclear isolation buffer (that is, nuclear fraction), sonicated and mixed with 4× Laemmli sample buffer containing 10% (vol/vol) 2-mercaptoethanol to achieve a final 1× concentration, then heated at 95 °C for 5 min and centrifuged at 15,000*g* for 3 min at room temperature before the supernatant was taken for SDS-PAGE. The same volumes—and thus the same whole-cell equivalents—of the whole-cell sample, cytosolic fraction, organellar fraction and nuclear fraction were loaded on the SDS-PAGE gel.

CAKI-2 cells did not perform well using the aforementioned protocol for HCT116 cells, so modifications were made. A suspension of 3 million live CAKI-2 cells in 500 μl ice-cold DPBS was prepared. From this, 200 μl of the cell suspension (that is, whole cells) was centrifuged at 500*g* for 5 min at 4 °C. The supernatant was aspirated and the pellet was resuspended with 200 μl lysis buffer (50 mM Tris-HCl (pH 7.5), 150 mM NaCl, 0.5% (vol/vol) NP-40 alternative, one-quarter of a tablet of cOmplete Protease Inhibitor Cocktail per 5 ml lysis buffer and half a tablet of PhosSTOP per 5 ml lysis buffer), incubated with rotation for 30 min at 4 °C and cleared by centrifugation at 17,000*g* for 10 min at 4 °C. 150 μl of the clarified lysate was mixed with 4× Laemmli sample buffer containing 10% (vol/vol) 2-mercaptoethanol to achieve a final 1× concentration, then heated at 95 °C for 5 min and centrifuged at 15,000*g* for 3 min at room temperature before the supernatant was taken for SDS-PAGE. The remaining 300 μl of the cell suspension in DPBS was centrifuged at 500*g* for 5 min at 4 °C and the supernatant was discarded. The pellet was resuspended in 300 μl of the kit’s cytosolic isolation buffer, vortexed for 5 s, left on ice for 5 min and then centrifuged at 500*g* for 5 min at 4 °C. 150 μl of the supernatant (that is, cytosolic fraction) was mixed with 4× Laemmli sample buffer containing 10% (vol/vol) 2-mercaptoethanol to achieve a final 1× concentration, then heated at 95 °C for 5 min and centrifuged at 15,000*g* for 3 min at room temperature before the supernatant was taken for SDS-PAGE. The remainder of the cytosolic fraction was aspirated off and the pellet was resuspended in 300 μl lysis buffer, incubated with rotation for 30 min at 4 °C and cleared by centrifugation at 17,000*g* for 10 min at 4 °C. 150 μl of the clarified lysate (that is, organellar fraction) was mixed with 4× Laemmli sample buffer containing 10% (vol/vol) 2-mercaptoethanol to achieve a final 1× concentration, then heated at 95 °C for 5 min and centrifuged at 15,000*g* for 3 min at room temperature before the supernatant was taken for SDS-PAGE. The same volumes—and thus the same whole-cell equivalents—of the whole-cell samples, cytosolic fractions and organellar fractions were loaded on the SDS-PAGE gel.

HEK293T cells did not perform well using the aforementioned protocol for HCT116 cells, so modifications were made. A suspension of 6 million live HEK293T cells in 600 μl ice-cold DPBS was prepared. From this, 200 μl of the cell suspension (that is, whole cells) was centrifuged at 500*g* for 5 min at 4 °C. The supernatant was aspirated and the pellet was resuspended with 200 μl lysis buffer (50 mM Tris-HCl (pH 7.5), 150 mM NaCl, 0.5% (vol/vol) NP-40 alternative, one-quarter of a tablet of cOmplete Protease Inhibitor Cocktail per 5 ml lysis buffer and half a tablet of PhosSTOP per 5 ml lysis buffer), incubated with rotation for 30 min at 4 °C and cleared by centrifugation at 17,000*g* for 10 min at 4 °C. 150 μl of the clarified lysate was mixed with 4× Laemmli sample buffer containing 10% (vol/vol) 2-mercaptoethanol to achieve a final 1× concentration and then heated at 72 °C for 10 min for SDS-PAGE. 300 μl of the cell suspension in DPBS was centrifuged at 500*g* for 5 min at 4 °C and the supernatant was discarded. The pellet was resuspended in 300 μl of the kit’s cytosolic isolation buffer, vortexed for 5 s, left on ice for 5 min and then centrifuged at 500*g* for 5 min at 4 °C. 150 μl of the supernatant (that is, cytosolic fraction) was mixed with 4× Laemmli sample buffer containing 10% (vol/vol) 2-mercaptoethanol to achieve a final 1× concentration and then heated at 72 °C for 10 min for SDS-PAGE. The remainder of the cytosolic fraction was aspirated off and the pellet was resuspended in 300 μl lysis buffer, incubated with rotation for 30 min at 4 °C and cleared by centrifugation at 17,000*g* for 10 min at 4 °C. 150 μl of the clarified lysate (that is, organellar fraction) was mixed with 4× Laemmli sample buffer containing 10% (vol/vol) 2-mercaptoethanol to achieve a final 1× concentration and then heated at 72 °C for 10 min for SDS-PAGE. The same volumes—and thus the same whole-cell equivalents—of the whole-cell samples, cytosolic fractions and organellar fractions were loaded on the SDS-PAGE gel.

#### Preparation of human cellular lysates

Preparation of cellular lysates from human cells was done at 4 °C or on ice unless otherwise indicated. Fully confluent 10-cm dishes were washed once with ice-cold DPBS and immediately scraped into 500 μl lysis buffer (50 mM Tris-HCl (pH 7.5), 150 mM NaCl, 0.5% (vol/vol) NP-40 alternative, one tablet of cOmplete Protease Inhibitor Cocktail per 25 ml lysis buffer and one tablet of PhosSTOP per 10 ml lysis buffer). Lysates were incubated with rotation for 30 min at 4 °C before being cleared by centrifugation at 17,000*g* for 10 min at 4 °C. Protein quantification was performed on the clarified lysate using the DC protein assay. For the preparation of samples for SDS-PAGE, clarified lysates were adjusted to have equal protein concentrations across samples, mixed with 4× Laemmli sample buffer containing 10% (vol/vol) 2-mercaptoethanol to achieve a final 1× concentration and then heated at 72 °C for 10 min.

#### Immunoblotting of samples from human cells

Samples were resolved by SDS-PAGE via 4–20% Mini-PROTEAN TGX gels (456-1096; Bio-Rad) and then transferred to 0.2-μm nitrocellulose membranes (1704158 and 1704159; Bio-Rad) using the Trans-Blot Turbo transfer system (1704150; Bio-Rad). Membranes were then blocked for 45 min at room temperature with 5% non-fat dry milk (1706404; Bio-Rad) prepared in 1× TBST (40120065-2; bioWORLD) and incubated with primary antibodies in 1× TBST overnight at 4 °C at the following dilutions: ACAA1 (1/250), AGPS (1/1,000), C6ORF226 (1/1,000), PEX5 (1/1,000), PEX13 (1/1,000), ACTB (1/2,500), PEX7 (1/1,000), PHYH (1/1,000), SCP2 (1/500), CANX (1/1,000), citrate synthase (1/1,000), GAPDH (1/5,000), HA (1/1,000), histone H3 (1/1,500) and RPS6KB1 (1/1,000). Afterwards, membranes were washed three times with 1× TBST and then incubated with HRP-conjugated secondary antibodies (1/5,000) in 1× TBST for 45 min at room temperature. Membranes were then washed three more times with 1× TBST and subsequently visualized using film or a ChemiDoc MP Imaging System (Bio-Rad) in conjunction with enhanced chemiluminescence via Pierce ECL Western Blotting Substrate (32106; Thermo Fisher Scientific) or, if increased sensitivity was needed, SuperSignal West Femto Maximum Sensitivity Substrate (34096; Thermo Fisher Scientific). If membranes needed to be stripped and re-probed after enhanced chemiluminescence, they were washed twice with 1× TBST, incubated with Restore PLUS Western Blot Stripping Buffer (46430; Thermo Fisher Scientific) for 15 min at room temperature, washed once more with 1× TBST and then blocked and immunoblotted as described already.

#### Quantification of immunoblots

Densitometric analyses of immunoblots were performed using the software ImageJ (version 1.54d)^89^ and background signals were corrected for. For the quantification of immunoblots, band intensities were used as indicated in the respective figures and figure captions.

### Experiments with HeLa cells

#### Cell culture conditions

HeLa cells (CCL-2; ATCC) were cultured in Dulbecco’s modified Eagle’s medium (41966-029; Gibco) plus 25 mM of HEPES buffer (J848; VWR) containing 10% FBS (FBS-12A; Capricorn Scientific), 100 U ml^−1^ penicillin and 100 μg ml^−1^ streptomycin (15140-122; Gibco), under a humidified atmosphere of 5% CO_2_ at 37 °C. Cells were routinely tested for *Mycoplasma*.

#### Generation of *HsPEX13*-KO cells

*HsPEX13*-KO cells were generated by CRISPR–Cas9, as described previously^94^. HeLa cells were transfected with the pSpCas9(BB)-2A-GFP vector (48138; Addgene) containing an sgRNA against *HsPEX13* using jetPRIME (114-15; Polyplus Transfection). The oligonucleotide sequences used for the sgRNA are provided in Supplementary Table 1. eGFP-expressing cells were sorted by FACS and cells were plated at one cell per well in 96-well plates and cultured at 37 °C as described above. After 6–8 weeks, *HsPEX13* KOs were confirmed by Sanger sequencing analysis of genomic DNA isolated from the expanded cell clones. After PCR amplification of exon 2 of *HsPEX13* using the gene-specific primers *HsPEX13*_F and *HsPEX13*_R, the PCR products were sequenced with −21M13 or M13rev primers using the Big Dye Terminator v3.1 Cycle Sequencing Kit (4337449; Thermo Fisher Scientific) and analysed on an ABI 3730 sequencer (Applied Biosystems). The primer sequences used are provided in Supplementary Table 1. Three clonal *HsPEX13*-KO cell lines were selected for follow-up studies with the following genotypes:

*HsPEX13*-KO_1 (c.142_143del (p.Leu48TyrfsX18)/c.142_143del (p.Leu48TyrfsX18))

*HsPEX13*-KO_2 (c.129_147del (p.Gly44GlnfsX19)/c.142_143del (p.Leu48TyrfsX18))

*HsPEX13*-KO_3 (c. 126_151del (p.Arg42SerfsX16)/c.142_143del (p.Leu48TyrfsX18))

#### Expression of wild-type and variant *Hs*PEX13

The three clonal *HsPEX13*-KO cell lines were transfected independently in duplicate with pcDNA3.1 (V79020; Thermo Fisher Scientific) containing wild-type *HsPEX13* or a variant with a K_10_PWE-to-AAAA substitution (*HsPEX13*(4A)) using jetPRIME (114-15; Polyplus Transfection) followed by 48 h of culturing at 37 °C, as described above. pcDNA3.1 containing *HsPEX13*(4A) was generated using GeneScript. 48 h after transfection, cells were taken for lysis and immunoblotting.

#### Lysis, immunoblotting and quantification of immunoblots

Cells were lysed in RIPA buffer (R0278; Sigma–Aldrich) plus cOmplete Mini protease inhibitors (11836153001; Roche). Proteins were separated on 10% NuPAGE gels (WG1203BX10; Invitrogen) and transferred onto nitrocellulose membranes. Immunoblot analysis was performed with antibodies against ACAA1 (HPA007244; Sigma–Aldrich; diluted 1/2,000), ACOX1 (ab184032; Abcam; diluted 1/2,000), AGPS (described previously^95^; diluted 1/3,000), *Hs*PEX13 (PAB22801; Abnova; diluted 1/2,000), PHYH (12858-1-AP; Proteintech; diluted 1/1,000) and TUBA4A (T6199; Sigma–Aldrich; diluted 1/2,000). After incubation with the secondary antibodies IRDye 800CW anti-rabbit (926-32211; LI-COR Biosciences) or IRDye 680RD anti-mouse (925-68070; LI-COR Biosciences) (both diluted 1/5,000), blots were imaged and band intensities quantified via an Odyssey Infrared Imaging System (LI-COR Biosciences).

### Experiments with recombinant and radiolabelled proteins

#### Human cell lines

For routine culturing and experiments, HEK293 cells (CRL-1573; ATCC) were grown at 37 °C under 5% CO_2_ and in Minimum Essential Medium Eagle (M2279; Sigma–Aldrich) supplemented with 2 mM l-alanyl-l-glutamine dipeptide (35050061; Gibco), 10% FBS (GA3160802; Gibco) and MycoZap Plus-CL (195261; Lonza). All complete media was passed through a 0.22-μm filter before use. Cells were routinely tested for *Mycoplasma*.

#### Plasmids

The plasmids pET28a-PEX7 (encoding an N-terminally histidine (His)-tagged version of human PEX7)^58^, pET23a-PEX5(C11K) (encoding a version of the large isoform of human PEX5 possessing a lysine at position 11, which improves the detection of ubiquitination under reducing conditions)^96^, pET28a-PEX5(1–324) (encoding a His-tagged protein comprising residues 1–324 of the large isoform of PEX5)^97^, pQE30-PEX5(315–639) (encoding a His-tagged protein comprising residues 315–639 of the large isoform of PEX5)^98^, pQE30-PEX14(1–80) (encoding an N-terminally His-tagged version of the first 80 residues of human PEX14 (NDPEX14))^98^, pGEM4-ACAA1 (encoding the precursor form of human ACAA1)^58^, pGEM4-PHYH (encoding the precursor form of human PHYH)^33^, pQE31-PHYH (encoding a His-tagged version of PHYH)^99^ and pGEM4-*Mm*SCPx (encoding mouse Sterol carrier protein 2)^100^ were all described before.

The following plasmids were produced:

1. pET28a-*Hs*PEX39 (encoding an N-terminally His-tagged version of *Hs*PEX39). The *HsPEX39* cDNA was amplified from total genomic DNA from HepG2 cells using the primers *Hs*.C6ORF226_EcoRI_Fw and *Hs*.C6ORF226_NotI_Rv. The amplified fragment was inserted into EcoRI/NotI-digested pET28a.
2. pGEX-*Hs*PEX39 (encoding an N-terminally GST-tagged version of *Hs*PEX39). The *HsPEX39* DNA fragment described above was inserted into the EcoRI/NotI sites of pGEX-6-P1 (GE HealthCare).
3. pGEX-*Hs*PEX39(1–70) (encoding an N-terminally GST-tagged *Hs*PEX39 containing residues 1–70). This was generated by introducing two stop codons by site-directed mutagenesis with the primers ORF70stop2x_Fw and _ ORF70stop2x_Rv.
4. pGEX-*Hs*PEX39(48–101) (encoding an N-terminally GST-tagged *Hs*PEX39 comprising residues 48–101). The corresponding DNA fragment was obtained by PCR with the primers *Hs*.C6ORF226_Glu48_EcoRI_Fw and *Hs*.C6ORF226_NotI_Rv and inserted into EcoRI/NotI sites of pGEX-6-P1 (GE HealthCare).
5. pGEX-*Hs*PEX39(KPWEmut) (encoding an N-terminally GST-tagged version of *Hs*PEX39 in which the KPWE motif is replaced by AAAA). This was obtained by overlap extension PCR. Two overlapping regions of *HsPEX39* cDNA, both encoding the KPWE-to-AAAA substitution, were obtained by PCR with the primer pairs *Hs*.C6ORF226_EcoRI_Fw/P2_KPWE-Ala_Rv and P3_KPWE-Ala_Fw/*Hs*.C6ORF226_NotI_Rv. The resulting PCR products were denatured, annealed and used in a third PCR with the primers *Hs*.C6ORF226_EcoRI_Fw and *Hs*.C6ORF226_NotI_Rv. The amplified fragment was digested and inserted into the EcoRI/NotI sites of pGEX-6-P1 (GE HealthCare).
6. pGEX-*Hs*PEX39(48–101,KPWEmut) (encoding an N-terminally GST-tagged *Hs*PEX39 protein comprising residues 48–101 in which the KPWE motif is substituted with AAAA). This was obtained by PCR amplification of pGEX-*Hs*PEX39(KPWEmut) with the primers *Hs*.C6ORF226_Glu48_EcoRI_Fw and *Hs*.C6ORF226_NotI_Rv. The resulting fragment was digested and inserted into EcoRI/NotI sites of pGEX-6-P1 (GE HealthCare).
7. pET23a-NtPEX13.Sumo.His. cDNA encoding residues 1–36 of *Hs*PEX13 appended to the TEV recognition sequence SENLYFQG, followed by residues 2–101 of human SUMO-1 protein with a C-terminal His-tag, was synthesized and cloned into the NdeI/BamHI restriction sites of pET23a (Novagen) by GenScript.
8. pET23a-NtPEX13(KPWEmut).Sumo.His. This was obtained by site-directed mutagenesis of the pET23a-NtPEX13.Sumo.His construct from GenScript.
9. pGEX-*Hs*PEX39(L21A) (encoding an N-terminally GST-tagged version of *Hs*PEX39 with the L21A substitution). This was obtained by site-directed mutagenesis of pGEX-*Hs*PEX39 using the primers *Hs*.C6ORF226_L21A_Fw and *Hs*.C6ORF226_L21A_Rv.
10. pET28a-PEX7(E77A) (encoding an N-terminally His-tagged version of human PEX7 with the E77A substitution). This was obtained by site-directed mutagenesis of pET28a-PEX7 with the primers PEX7_E77A_fw and PEX7_E77A_Rv.
11. pACEBac1-FLAGPEX7 (encoding an N-terminally FLAG-tagged version of human PEX7). The coding sequence for FLAG-PEX7, optimized for eukaryotic expression, was synthesized (GenScript Biotech) and cloned into the pACEBac1 plasmid.

#### Expression and purification of recombinant proteins

Recombinant His-PEX5(1–324) and His-PEX5(315–639) (both ref. ^98^), as well as NDPEX14 (ref. ^98^) and His-PHYH^99^, were expressed and purified exactly as described before.

The N-terminally His-tagged and GST-tagged versions of *Hs*PEX39 were expressed in *Escherichia coli* BL21(DE3) (AGLS230245; Agilent) for 3 h at 37 °C with 0.5 mM isopropyl β-d-1-thiogalactopyranoside and purified using either Ni Sepharose 6 Fast Flow (17-5318-01; Cytiva) or Glutathione Sepharose 4B (17-0756-01; Cytiva) affinity chromatography resins, according to the manufacturer’s instructions. All proteins were stored in 50 mM Tris-HCl (pH 8.0), 150 mM NaCl, 1 mM EDTA-NaOH (pH 8.0) and 1 mM DTT at −80 °C.

Where indicated, GST-*Hs*PEX39 variants were treated with His-tagged HRV-3C protease to remove the GST tag. Briefly, GST-tagged proteins were incubated with 3C protease (30:1 (wt/wt)) for 16 h at 4 °C in 50 mM Tris-HCl (pH 8.0), 150 mM NaCl, 1 mM EDTA-NaOH (pH 8.0) and 1 mM DTT. The His-tagged protease was removed from the preparation by affinity chromatography using Ni Sepharose 6 Fast Flow resin and collecting the flow through.

For FLAG-PEX7 expression in insect cells, bacmids were produced in DH10EMBacY *E. coli* cells and purified by lysing cells with the A1, A2 and A3 buffers of the Plasmid Miniprep Kit (740588.50; Macherey-Nagel), followed by isopropanol precipitation. Briefly, 700 μl isopropanol was applied to 800 μl cell lysate and centrifuged at 16,000*g* and 4 °C for 10 min. Pellets were washed with 200 μl 70% (vol/vol) ethanol, dried and solubilized in sterilized water.

To generate the virus stocks, 10 μg of each bacmid were added to 250 μl Sf-900 II serum-free medium (10902088; Thermo Fisher Scientific) and 4 μl FuGENE HD Transfection Reagent (E2311; Promega). The mixtures were transfected into 3 ml of 0.7 × 10^6^ cells per ml Sf9 cells and the supernatants—namely the V_0_ virus stocks—were collected after incubation at 27 °C for 72 h. To obtain the virus stocks at higher titration and in larger amounts, a V_1_ virus stock was generated by suspension culture of 1.0 × 10^6^ cells per ml Sf9 cells infected with 0.2% (vol/vol) V_0_ virus at 27 °C and 100 r.p.m. for 96 h and the supernatants were stored at 4 °C with 10% FBS (AU-FBS/PG; CellSera Australia).

For large-scale expression, 1.0 × 10^6^ cells per ml Hi5 cells were infected with 0.2% (vol/vol) V_1_ virus and cultured at 27 °C and 100 r.p.m. for 72 h. Hi5 cells were pelleted and resuspended with lysis buffer (50 mM HEPES (pH 8.0), 150 mM NaCl, 5 mM MgCl_2_, 10% glycerol, 1 mM phenylmethylsulfonyl fluoride, 1 mM benzamidine, 2 mM β-mercaptoethanol and 250 µl BioLock per 400 ml culture). Cells were disrupted using a Dounce homogenizer and then centrifuged at 58,540*g* and 6 °C for 60 min. The supernatant was collected and applied to a gravity-flow column filled with 2 ml anti-FLAG M2 Beads (A2220; Millipore) for affinity chromatography. Protein was eluted with 0.2 mg ml^−1^ FLAG Peptide in storage buffer (50 mM HEPES (pH 8.0), 150 mM NaCl, 5 mM MgCl_2_ and 10% glycerol). Elution fractions were analysed by SDS-PAGE and fractions containing FLAG-PEX7 were pooled, concentrated and stored at −80 °C.

#### In vitro synthesis of radiolabelled proteins

Radiolabelled proteins were synthesized in vitro using the TNT T7 Quick Coupled Transcription/Translation System (L1170; Promega) in the presence of EasyTag l-[^35^S]methionine (specific activity > 1,000 Ci mmol^−1^; NEG709A; PerkinElmer) for 90 min at 30 °C, according to the manufacturer’s instructions, with the exception of PEX7, which was synthesized for 4 h. Unlabelled (cold) PEX7 was synthesized in the same way, but using unlabelled methionine provided in the kit. Aliquots of the rabbit reticulocyte lysates containing the synthesized proteins were snap-frozen in liquid nitrogen and stored at −80 °C.

#### Cell-free in vitro assays

In vitro peroxisomal import reactions using HEK293-derived PNSs were performed as previously described^38^. Briefly, the rabbit reticulocyte lysates (RRLs) containing the radiolabelled reporter protein (0.2–1.0 μl RRL per import reaction) was diluted in 10 μl import buffer (0.25 M sucrose, 50 mM KCl, 20 mM MOPS-KOH (pH 7.2), 5 mM MgCl_2_, 2 μg ml^−1^ E-64 and 48 μg ml^−1^ methionine, final concentration). For improved import yields, the diluted [^35^S]ACAA1 RRL also contained 70 nM His-PEX5(1–324) and 1 μl RRL containing unlabelled PEX7 to form a pre-import complex between PEX5, PEX7 and the PTS2-containing protein. Radiolabelled proteins were added to import reactions containing 600 μg HEK293 PNS (previously primed by incubating at 37 °C for 5 min with 0.3 mM ATP (A2383; Sigma–Aldrich)) in import buffer supplemented with 5 μM bovine ubiquitin, 2 mM reduced glutathione and either 3 mM ATP or 3 mM AMP-PNP (NU-407; Jena Bioscience) (100 μl final volume). Where specified, in vitro reactions were supplemented with 2 μM ubiquitin aldehyde, His-PEX5(315–639) (5 μM), NDPEX14 (10 μM), NtPEX13.Sumo.His (up to 20 μM) and His.*Hs*PEX39 or untagged *Hs*PEX39 variants (up to 1 μM). Import reactions were incubated for 30 min at 37 °C. Import reactions with radiolabelled PTS2- or PTS1-containing proteins were treated with trypsin (400 μg ml^−1^ final concentration) to degrade non-imported reporter protein. After a 40-min incubation on ice, proteases were inhibited with phenylmethylsulfonyl fluoride (500 μg ml^−1^, final concentration). When monitoring radiolabelled PEX5, the protease treatment was omitted. Reactions were diluted with ice-cold SEMK (20 mM MOPS-KOH (pH 7.2), 0.25 M sucrose, 1 mM EDTA-NaOH (pH 8.0) and 80 mM KCl). Organelles, and soluble fractions (if required), were collected by centrifugation (20 min; 16,000*g*; 4 °C). Proteins were precipitated with 10% (wt/vol) TCA and washed with acetone and the resulting organelle and/or soluble fractions were analysed by SDS-PAGE, followed by immunoblotting, Ponceau S staining and autoradiography.

#### Native PAGE analyses

The reticulocyte lysate containing radiolabelled His-PEX7 (0.4 μl) was incubated in the absence or presence of recombinant *Hs*PEX39 variants (4 μg), His-PEX5(1–324) (1 μg), His-PHYH (100 ng) and NtPEX13.Sumo.His variants (4 μg), as specified in the figures, in 20 μl 20 mM Tris-HCl (pH 8.0), 5 mM DTT and 0.025% (wt/vol) bovine serum albumin. After adding 5 μl loading buffer (0.17% (wt/vol) bromophenol blue and 50% (wt/vol) sucrose), 6 μl of the samples were loaded onto Tris non-denaturing 8% polyacrylamide gels using a discontinuous buffer system^101^ and run at 250 V and 4 °C for ~1 h. For native PAGE assays using only recombinant proteins, approximately 5 µg FLAG-PEX7, GST-*Hs*PEX39, His-PEX5(1–324) and His-PHYH were analysed in the same way.

To determine the half-lives of protein complexes, [^35^S]His-PEX7 complexes were formed with 50 nM and 5 μM GST-*Hs*PEX39 and NtPEX13.Sumo.His and then incubated for 20 min with 5 μM GST-*Hs*PEX39(48–101) and 25 μM GST-*Hs*PEX39, respectively. Control samples (0 and 20 min, without an HsPEX39 competitor) were incubated with corresponding amounts of GST-Ub. Aliquots were withdrawn at the indicated time points and immediately flash-frozen in liquid nitrogen. Samples were individually thawed and loaded in the gel from the longest to shortest time points.

Gels were stained with Coomassie or blotted onto nitrocellulose membranes, stained with Ponceau S and subjected to autoradiography. For immunoblotting analysis of protein complexes detected by native PAGE, bands were excised from the wet stained nitrocellulose membranes, finely cut into smaller pieces and thoroughly destained with water. Proteins were eluted from the membrane slices with Laemmli sample buffer (30 min at 37 °C followed by 30 min at 65 °C) and analysed by SDS-PAGE and immunoblotting using antibodies directed against *Hs*PEX39 (HPA045350; Sigma–Aldrich; diluted 1/3,000) and PHYH (12858-1-AP; Proteintech; diluted 1/3,000) and antibodies raised in rabbit against human PEX7 (ref. ^58^; diluted 1/2,000) and PEX5 (ref. ^102^; diluted 1/6,000), as described elsewhere. Primary antibodies were detected using alkaline phosphatase-conjugated anti-rabbit antibody (A9919; Sigma–Aldrich; diluted 1/20,000).

#### SEC

SEC was performed on an ÄKTA pure system using a Superdex 200 Increase 3.2/300 equilibrated with 50 mM Tris-HCl (pH 7.5), 150 mM NaCl and 1 mM EDTA at a flow rate of 0.075 ml min^−1^. Proteins (10–15 µg), alone or mixed together, were incubated for 15 min at 23 °C before injection. Fractions of 70 µl were collected and analysed by SDS-PAGE. Ferritin (440 kDa), bovine serum albumin (66 kDa) and ribonuclease A (13.7 kDa) were used as molecular weight markers.

### Bioinformatics analyses and structural predictions

#### Sequence analysis of (R/K)PWE motif sequences

Sequences with a significant relationship to human C6ORF226 were initially established by BLAST^103^ searches in the UniProt database using *P* < 0.001 as a significant threshold. The sequence set was further expanded by three rounds of iterative refinement using the generalized profile method^104^ and *P* < 0.001 as a threshold for including protein hits in the next iteration. Protein alignments were calculated using the L-INS-i algorithm of the MAFFT package (version 7.490)^105^. In the second and third iteration cycles, significant hits in members of the PEX13 family were picked up, based on conservation of the KPWE motif. Subsequently, the PEX13 family was analysed by a separate set of generalized profiles, as described for the C6ORF226 family. The final hit lists after three rounds of iterative refinement were used to compile Extended Data Fig. 1a.

The conserved KPWE core region and five flanking residues on either side were extracted from the final C6ORF226 and PEX13 family alignments and used for sequence logo generation using WebLogo version 2.8.2 (https://weblogo.berkeley.edu)^106,107^. The final hit lists and alignment files for the C6ORF226 and PEX13 families are available from the corresponding authors upon request.

#### AlphaFold structural prediction of protein complexes

For the structural predictions of protein complexes, the amino acid sequences of the proteins were downloaded from UniProt (https://www.uniprot.org/) and indicated parts were used for web-based predictions (https://colab.research.google.com/github/sokrypton/ColabFold/blob/main/AlphaFold2.ipynb), as described previously^42,108^. The highest-ranking model of each prediction was visualized using ChimeraX (version 1.7.1.0)^109^. Parameters and output files of the predictions are available from the corresponding authors upon request.

#### Superimposition of yeast and human PEX7 structural models from the AlphaFold database

Structural models deposited in the AlphaFold database (https://alphafold.ebi.ac.uk/) were downloaded as .pdb files using the accession codes AF-O00628-F1-v4 (*Hs*PEX7) and AF-P39108-F1-v4 (*Sc*Pex7). The respective .pdb files were loaded into ChimeraX (version 1.7.1.0)^109^ and superimposed using the matchmaker command (Extended Data Fig.  10).

### Statistics and reproducibility

Statistics were calculated using GraphPad Prism 10 and Microsoft Excel, unless otherwise specified. No statistical methods were used to pre-determine sample sizes, but our sample sizes are similar to those reported in previous publications. Definitions of central and dispersion or precision measures, as well as the statistical tests used are included in the figure captions. A significance threshold of 0.05 was used for all of the statistical tests in Figs. 1c, 2c,e,g, 3b and 6i and Extended Data Figs. 4a, 5c and 7f,h. For the limma approach used in Fig. 2e, we formally assessed the equality of variances using Levene’s test and found them to be equal at a 1% significance level. Although formal tests for normality (Shapiro–Wilk and D’Agostino–Pearson tests) indicated deviations from perfect normality, the distributions were manually inspected and found to be sufficiently close to normality to justify the use of the limma approach. The data distribution in our study was otherwise assumed to be normal and was not formally tested. All of the experiments were performed independently at least twice with similar results (that is, two biological replicates), except for those shown in Extended Data Figs. 3d, 5i (right side; extractions with low- and high-salt buffers) and 9b (bottom panel), for which only one biological replicate was performed. For Fig. 6i, data from two independent experiments utilizing the three independent *HsPEX13*-KO clones transfected with the indicated plasmids are presented in aggregate accordingly (that is, six biological replicates). For all instances for which *n* replicates are reported (for example, *n* = 3), these are biological replicates. No data points were excluded from the analyses except for the OD_600_measurement of one of the replicates for wild-type yeast cells grown for 96 h in oleic acid medium (Fig. 6f), which was excluded because the sample had visible microbial contamination that would have rendered the OD_600_ measurement inaccurate. SILAC labelling of *Scpex39*Δ and wild-type yeast cells was switched between biological replicates to avoid bias introduced by the SILAC label, but randomization was otherwise not performed in this study because it was not relevant (for example, control and KO cells were already defined). Data collection and analysis were not performed blind to the conditions of the experiments, but were performed without bias.

### Reporting summary

Further information on research design is available in the Nature Portfolio Reporting Summary linked to this article.

## Online content

Any methods, additional references, Nature Portfolio reporting summaries, source data, extended data, supplementary information, acknowledgements, peer review information; details of author contributions and competing interests; and statements of data and code availability are available at 10.1038/s41556-025-01711-z.

## Supplementary information

Reporting Summary

Supplementary Tables Supplementary Tables 1–4.

## Source data

Source Data Figs. 1–8 and Extended Data Figs. 1–9 Full-length, unprocessed gels and blots.

Source Data Fig. 2 Statistical source data.

Source Data Fig. 3 Statistical source data.

Source Data Fig. 5 Statistical source data.

Source Data Fig. 6 Statistical source data.

Source Data Extended Data Fig. 3 Statistical source data.

Source Data Extended Data Fig. 4 Statistical source data.

Source Data Extended Data Fig. 5 Statistical source data.

Source Data Extended Data Fig. 7 Statistical source data.

## Data Availability

All unique and stable reagents generated in this study are available upon reasonable request, but may require a completed Materials Transfer Agreement. The mass spectrometry proteomics data have been deposited to the ProteomeXchange Consortium via the PRIDE^110^ partner repository with the dataset identifiers PXD051501 (Pex18-TPA affinity purification experiments) and PXD051550 (*Scpex39*Δ yeast SILAC experiments). UniProt data were acquired from https://www.uniprot.org/. Source data are provided with this paper. All other data supporting the findings of this study are available from the corresponding authors upon reasonable request.

## Code availability

Original code used for analysis of the mass spectrometric data is available at https://github.com/ag-warscheid/Pex39_Manuscript and 10.5281/zenodo.11933133 (ref. ^77^).
