# Supplementary material for: PEX39 facilitates the peroxisomal import of PTS2-containing proteins
Source: Nat Cell Biol. 2025 Jul 30;27(8):1256–71. doi: 10.1038/s41556-025-01711-z (PMC12339391; doi:10.1038/s41556-025-01711-z)
Supplement: Supplementary file 1 — Reporting Summary [file 41556_2025_1711_MOESM1_ESM.pdf]

nature portfolio

Corresponding author(s): Walter W. Chen, Ralph J. DeBerardinis, Jorge E. Azevedo, Bettina Warscheid

Last updated by author(s): May 7, 2025

# Reporting Summary

Nature Portfolio wishes to improve the reproducibility of the work that we publish. This form provides structure for consistency and transparency in reporting. For further information on Nature Portfolio policies, see our Editorial Policies and the Editorial Policy Checklist.

## Statistics

For all statistical analyses, confirm that the following items are present in the figure legend, table legend, main text, or Methods section.

| n/a | Confirmed | |
|---|---|---|
| ☐ | ☒ | The exact sample size (*n*) for each experimental group/condition, given as a discrete number and unit of measurement |
| ☐ | ☒ | A statement on whether measurements were taken from distinct samples or whether the same sample was measured repeatedly |
| ☐ | ☒ | The statistical test(s) used AND whether they are one- or two-sided<br>*Only common tests should be described solely by name; describe more complex techniques in the Methods section.* |
| ☐ | ☒ | A description of all covariates tested |
| ☐ | ☒ | A description of any assumptions or corrections, such as tests of normality and adjustment for multiple comparisons |
| ☐ | ☒ | A full description of the statistical parameters including central tendency (e.g. means) or other basic estimates (e.g. regression coefficient) AND variation (e.g. standard deviation) or associated estimates of uncertainty (e.g. confidence intervals) |
| ☐ | ☒ | For null hypothesis testing, the test statistic (e.g. *F*, *t*, *r*) with confidence intervals, effect sizes, degrees of freedom and *P* value noted<br>*Give P values as exact values whenever suitable.* |
| ☒ | ☐ | For Bayesian analysis, information on the choice of priors and Markov chain Monte Carlo settings |
| ☒ | ☐ | For hierarchical and complex designs, identification of the appropriate level for tests and full reporting of outcomes |
| ☒ | ☐ | Estimates of effect sizes (e.g. Cohen's *d*, Pearson's *r*), indicating how they were calculated |

*Our web collection on statistics for biologists contains articles on many of the points above.*

## Software and code

Policy information about availability of computer code

| | |
|---|---|
| Data collection | Densitometric quantification of immunoblots was performed using the software ImageJ (v1.54d). Band intensities of immunoblots were also quantified via an Odyssey Infrared Imaging System (LI-COR Biosciences). Yeast fluorescent microscopy images acquired using a microscope operated by ScanR (v3.2.0). |
| Data analysis | Statistics were calculated using GraphPad Prism 10 and Microsoft Excel unless specified otherwise. For sequence analysis of [R/K]PWE motifs across eukaryotes, BLAST and the L-Ins-I algorithm of the MAFFT package (v7.490) were used. Mass spectrometry data processing and analysis for Pex18-TPA experiment: Andromeda search engine implemented in MaxQuant (v2.0.1.0); the autoprot Python module (v0.2); R package RankProd. For protein identification and SILAC-based relative quantification in Scpex39Δ-versus-wild-type yeast experiments, MaxQuant/ Andromeda (v2.4.4.0) was employed. Original code for the analysis of mass spectrometric data is available at https://github.com/ag-warscheid/Pex39_Manuscript and https://zenodo.org/records/11933133. STRING network analysis was performed using the STRING web application (v12) (https://string-db.org/). GO term enrichment analyses were performed using the g:Profiler application (https://biit.cs.ut.ee/gprofiler/gost). Web-based protein structural predictions: https://colab.research.google.com/github/sokrypton/ColabFold/blob/main/AlphaFold2.ipynb; highest-ranking model of each prediction was visualized using ChimeraX (v1.7.1.0). WebLogo graphics generated via WebLogo (v2.8.2) (https://weblogo.berkeley.edu). |

For manuscripts utilizing custom algorithms or software that are central to the research but not yet described in published literature, software must be made available to editors and reviewers. We strongly encourage code deposition in a community repository (e.g. GitHub). See the Nature Portfolio guidelines for submitting code & software for further information.

# Data

Policy information about availability of data

All manuscripts must include a data availability statement. This statement should provide the following information, where applicable:

- Accession codes, unique identifiers, or web links for publicly available datasets
- A description of any restrictions on data availability
- For clinical datasets or third party data, please ensure that the statement adheres to our policy

All unique and stable reagents generated in this study are available upon reasonable request, but may require a completed Materials Transfer Agreement. The mass spectrometry proteomics data have been deposited to the ProteomeXchange Consortium via the PRIDE partner repository with the dataset identifiers PXD051501 (Pex18-TPA affinity purification experiments) and PXD051550 (Scpex39Δ yeast SILAC experiments). Uniprot data was acquired from https://www.uniprot.org/. Source data are provided with this paper. All other data supporting the findings of this study are available from the corresponding authors on reasonable request.

# Research involving human participants, their data, or biological material

Policy information about studies with human participants or human data. See also policy information about sex, gender (identity/presentation), and sexual orientation and race, ethnicity and racism.

| | |
|---|---|
| Reporting on sex and gender | Not relevant to this study. |
| Reporting on race, ethnicity, or other socially relevant groupings | Not relevant to this study. |
| Population characteristics | Not relevant to this study. |
| Recruitment | Not relevant to this study. |
| Ethics oversight | Not relevant to this study. |

Note that full information on the approval of the study protocol must also be provided in the manuscript.

# Field-specific reporting

Please select the one below that is the best fit for your research. If you are not sure, read the appropriate sections before making your selection.

☒ Life sciences ☐ Behavioural & social sciences ☐ Ecological, evolutionary & environmental sciences

For a reference copy of the document with all sections, see nature.com/documents/nr-reporting-summary-flat.pdf

# Life sciences study design

All studies must disclose on these points even when the disclosure is negative.

| | |
|---|---|
| Sample size | All experiments had at least two biological replicates, except for those shown in Extended Data Figs. 3d, 5i (right side, extractions with low- and high-salt buffers), 9b (bottom panel). Experimental data with accompanying statistical tests had at least three biological replicates to achieve a sufficient size for statistical analysis. No statistical methods were used to pre-determine sample sizes but our sample sizes are similar to those reported in previous publications. |
| Data exclusions | No data points were excluded from the analyses except for the OD600 measurement of one of the replicates for wild-type yeast cells grown for 96 hours in oleic acid medium (Fig. 6f), which was excluded because the sample had visible microbial contamination that would have rendered the OD600 measurement inaccurate. |
| Replication | All experiments had at least two biological replicates, except for those shown in Extended Data Figs. 3d, 5i (right side, extractions with low- and high-salt buffers), 9b (bottom panel). All experiments had similar outcomes across biological replicates. |
| Randomization | SILAC labeling of Scpex39Δ and wild-type yeast cells was switched between biological replicates to avoid bias introduced by the SILAC label, but randomization otherwise was not done in this study because it was not relevant (for example, control and knockout cells were already defined). |
| Blinding | Data collection and analysis were not performed blind to the conditions of the experiments but were done without bias. |

# Reporting for specific materials, systems and methods

We require information from authors about some types of materials, experimental systems and methods used in many studies. Here, indicate whether each material, system or method listed is relevant to your study. If you are not sure if a list item applies to your research, read the appropriate section before selecting a response.

## Materials & experimental systems

| n/a | Involved in the study |
|---|---|
| ☐ | ☒ Antibodies |
| ☐ | ☒ Eukaryotic cell lines |
| ☒ | ☐ Palaeontology and archaeology |
| ☒ | ☐ Animals and other organisms |
| ☒ | ☐ Clinical data |
| ☒ | ☐ Dual use research of concern |
| ☒ | ☐ Plants |

## Methods

| n/a | Involved in the study |
|---|---|
| ☒ | ☐ ChIP-seq |
| ☒ | ☐ Flow cytometry |
| ☒ | ☐ MRI-based neuroimaging |

# Antibodies

Antibodies used

Antibodies to ACAA1 (HPA006764 and HPA007244), AGPS (HPA030211), C6ORF226 (HPA045350), PEX5 (HPA039260), HsPEX13 (HPA032142), TUBA4A (T6199), and alkaline phosphatase-conjugated anti-rabbit antibody (A9919) were from Sigma-Aldrich; the antibody to ACTB (sc-69879) was from Santa Cruz Biotechnology; antibodies to PEX7 (20614-1-AP), PHYH (12858-1-AP), SCP2 (23006-1-AP) were from Proteintech; antibodies to CANX (2433), CS (14309), GAPDH (2118), HA (3724), Histone H3 (3638), and RPS6KB1 (2708), as well as HRP-conjugated anti-rabbit secondary antibody (7074) were from Cell Signaling Technology; the antibody to ACOX1 (ab184032) and the HRP-conjugated anti-mouse secondary antibody for IP (ab131368) were from Abcam. Additional antibodies include: antibodies to AGPS (DOI: 10.1111/j.1432-1033.1997.00511.x, gift from Edwin de Vet of Utrecht University), PEX7 (DOI: 10.1128/MCB.01727-13, made for prior study by core facility of IBMC, Universidade do Porto), and PEX5 (DOI: 10.1074/jbc.M004366200, made for prior study by core facility of IBMC, Universidade do Porto) that were described previously; an antibody to HsPEX13 from Abnova (PAB22801); the secondary antibodies IRDye 800CW anti-rabbit (926-32211) or IRDye 680RD anti-mouse (925-68070) from LI-COR Biosciences; goat polyclonal anti-Cta1 (S. cerevisiae) (DOI: 10.1083/jcb.120.3.665, gift from Andreas Hartig of Max Perutz Labs); rabbit polyclonal anti-Pex7 (S. cerevisiae) (DOI: 10.1128/MCB.22.17.6056-6069.2002), rabbit polyclonal anti-Pex3 (S. cerevisiae), rabbit polyclonal anti-Pex18 (S. cerevisiae) (DOI: 10.1111/j.1600-0854.2008.00876.x), rabbit polyclonal anti-Pot1 (S. cerevisiae) (DOI: 10.1002/yea.320100905), rabbit polyclonal anti-Gpd1 (S. cerevisiae) (DOI: 10.1074/jbc.M115.653451) were gifts from Ralf Erdmann of Ruhr University Bochum; rabbit polyclonal anti-Protein A (S. aureus) (P3775), goat HRP-conjugated polyclonal anti-rabbit (A0545), rabbit HRP-conjugated polyclonal anti-goat (A8919), rabbit HRP-conjugated polyclonal anti-mouse (A9044) from Sigma-Aldrich; mouse monoclonal anti-Por1 (S. cerevisiae) (459500, clone: 16G9E6BC4), mouse monoclonal anti-Pgk1 (S. cerevisiae) (459250, clone: 22C5D8), mouse monoclonal anti-Dpm1 (S. cerevisiae) (A-6429, clone: 5C5A7) were from Invitrogen.

Validation

All primary antibodies used in this study are commercially available or from the indicated sources and have been validated by the manufacturer and/or previously published studies. ACAA1 (HPA006764), PEX7 (20614-1-AP), and PHYH (12858-1-AP) antibodies were additionally validated in this study by detection of epitope-tagged versions of corresponding target proteins via immunoblotting. The antibody to C6ORF226 (HPA045350) was additionally validated by immunoblotting using wild-type, C6ORF226-knockout, and C6ORF226-overexpressing human cell lines.

# Eukaryotic cell lines

Policy information about cell lines and Sex and Gender in Research

Cell line source(s)

HEK293 (ATCC CRL-1573), HEK293T (ATCC CRL-3216), HCT116 (ATCC CCL-247), NCI-H1792 (ATCC CRL-5895), and HeLa (ATCC CCL-2) cells were purchased from ATCC. CAKI-2 cells were a gift from Gregory Wyant and William Kaelin Jr.

Authentication

HEK293, HEK293T, HCT116, NCI-H1792, CAKI-2, and HeLa cells were authenticated via Short Tandem Repeat profiling.

Mycoplasma contamination

All cells were negative for mycoplasma contamination and routinely tested.

Commonly misidentified lines
(See ICLAC register)

No commonly misidentified cell lines were used.

# Plants

Seed stocks

No plants used.

Novel plant genotypes

No plants used.

Authentication

No plants used.
