# Supplementary material for: PEX39 facilitates the peroxisomal import of PTS2-containing proteins
Source: Nat Cell Biol. 2025 Jul 30;27(8):1256–71. doi: 10.1038/s41556-025-01711-z (PMC12339391; doi:10.1038/s41556-025-01711-z)

Figure 1d

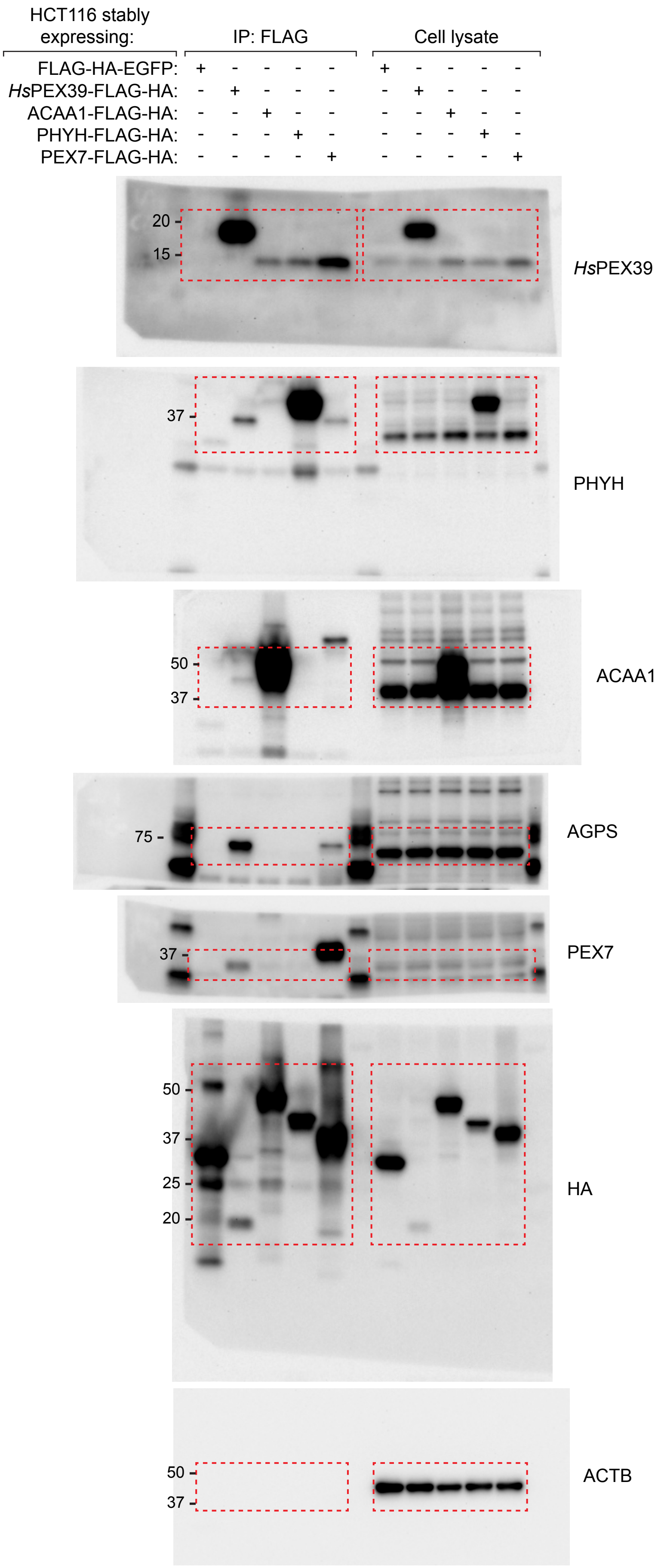
HCT116 stably expressing:
IP: FLAG
Cell lysate
FLAG-HA-EGFP: + - - - - + - - - -
HsPEX39-FLAG-HA: - + - - - - + - - -
ACAA1-FLAG-HA: - - + - - - - + - -
PHYH-FLAG-HA: - - - + - - - - + -
PEX7-FLAG-HA: - - - - + - - - - +
20
15
HsPEX39
37
PHYH
50
37
ACAA1
75
AGPS
37
PEX7
50
37
25
20
HA
50
37
ACTB

Figure 1e

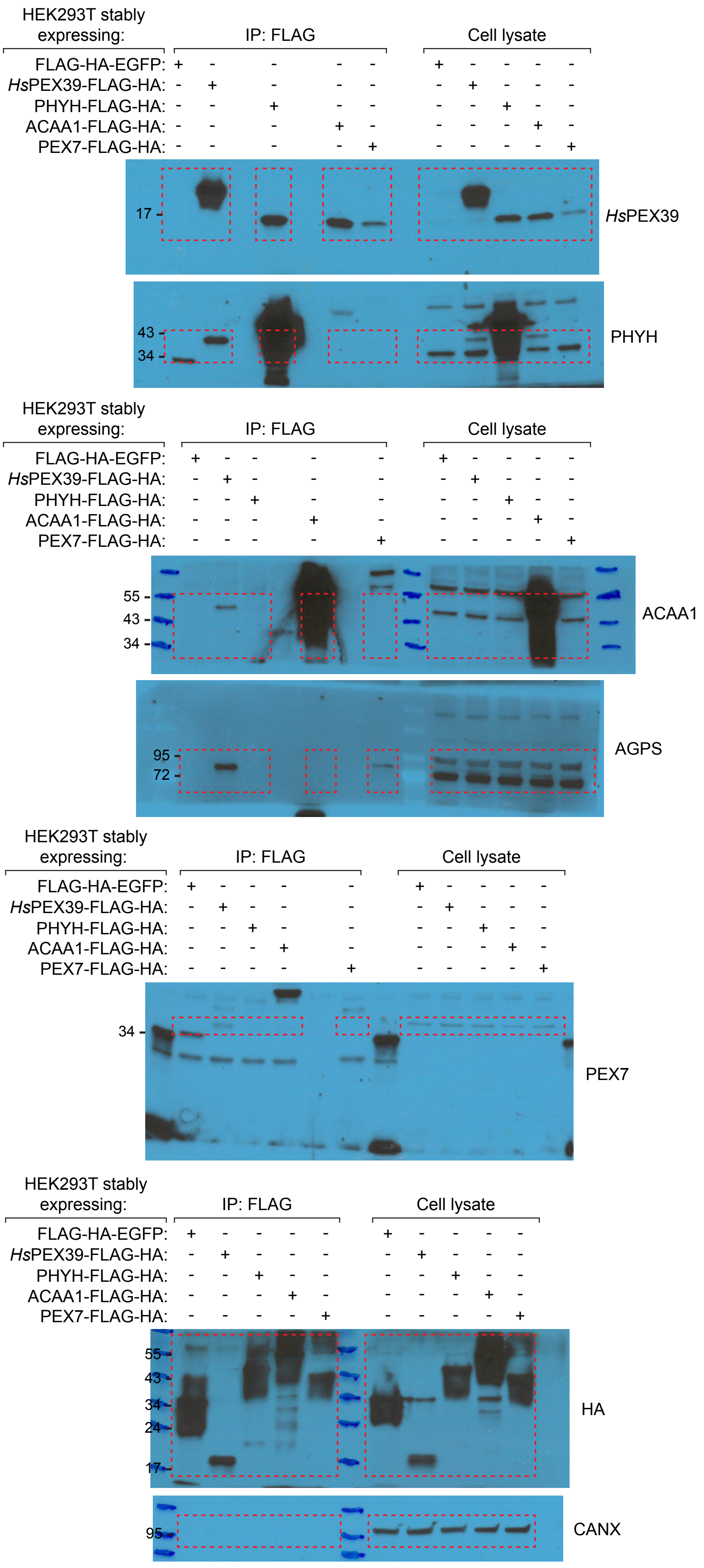

Figure 1f

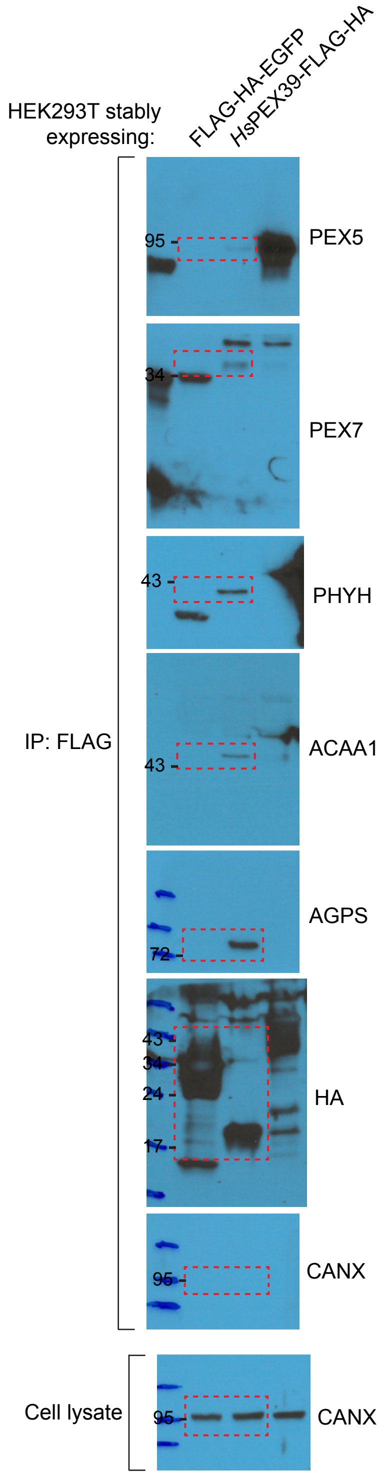

Figure 1g

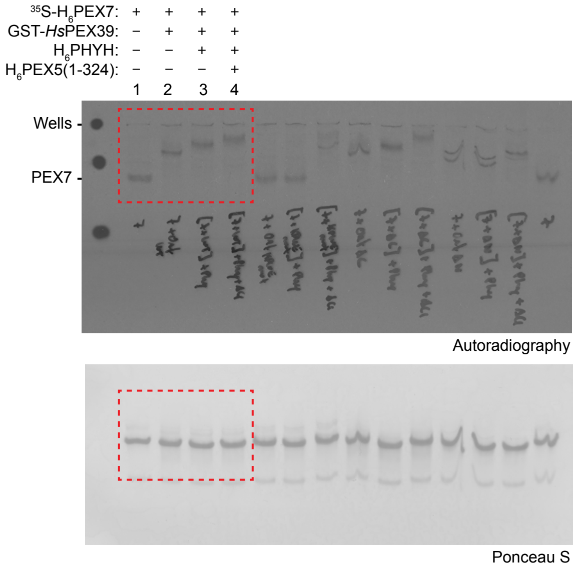

^{35}S-H$_6$PEX7: + + + +
GST-*Hs*PEX39: − + + +
H$_6$PHYH: − − + +
H$_6$PEX5(1-324): − − − +
1 2 3 4
Wells
PEX7
Autoradiography
Ponceau S

Figure 2b

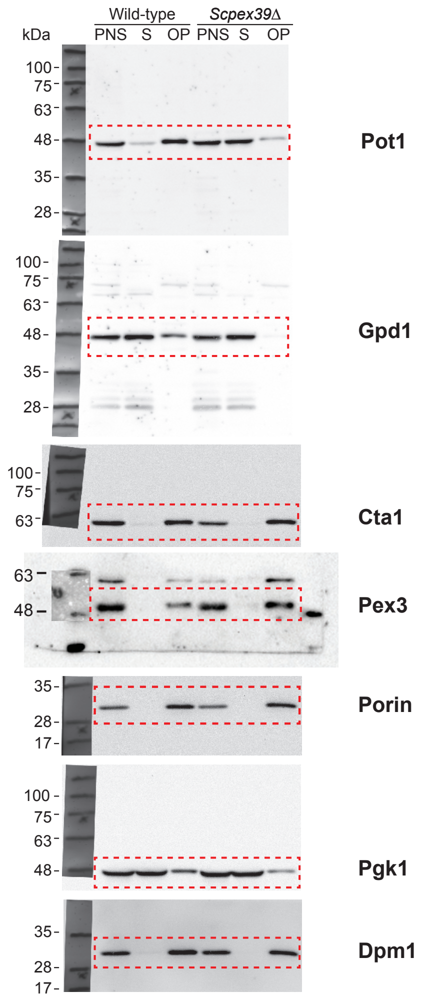
Wild-type
Scpex39Δ
kDa
PNS S OP PNS S OP
100
75
63
48
35
28
Pot1
Gpd1
Cta1
Pex3
Porin
17
Pgk1
Dpm1

Figure 2f

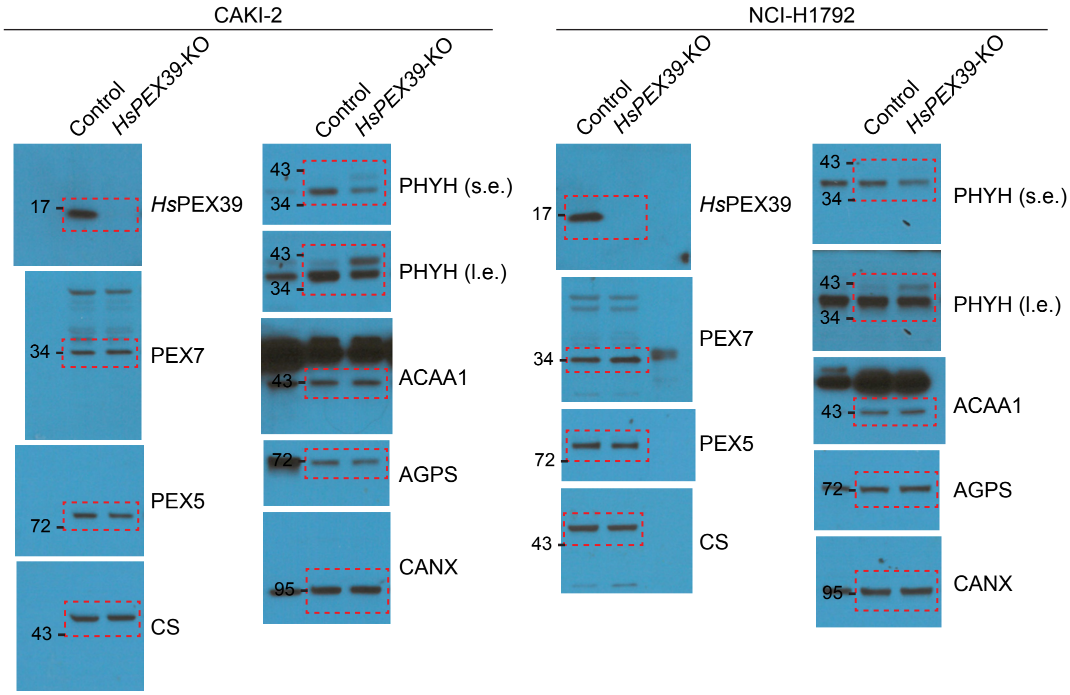

Figure 2h

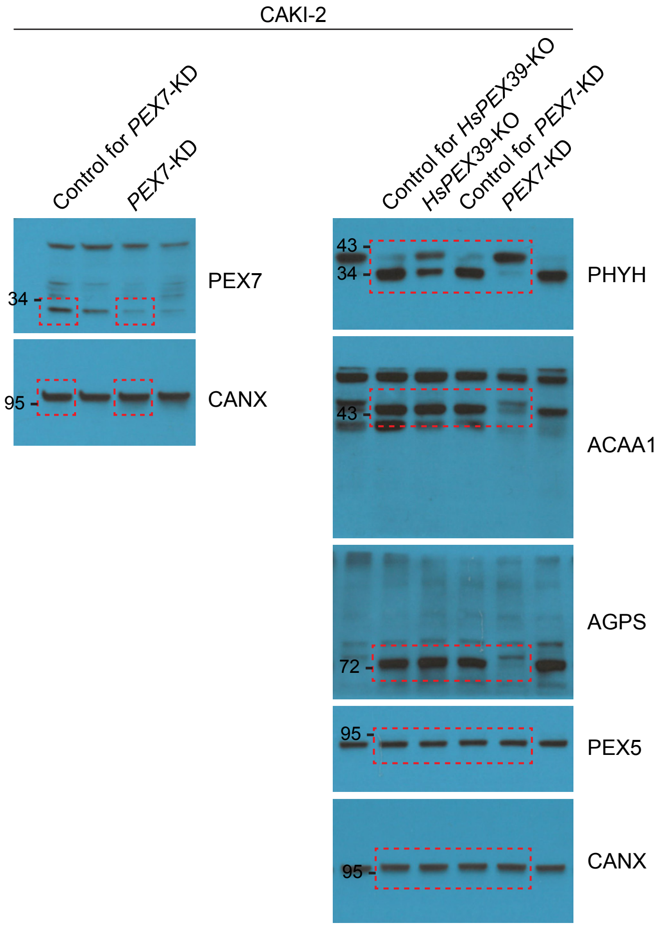

Figure 3a

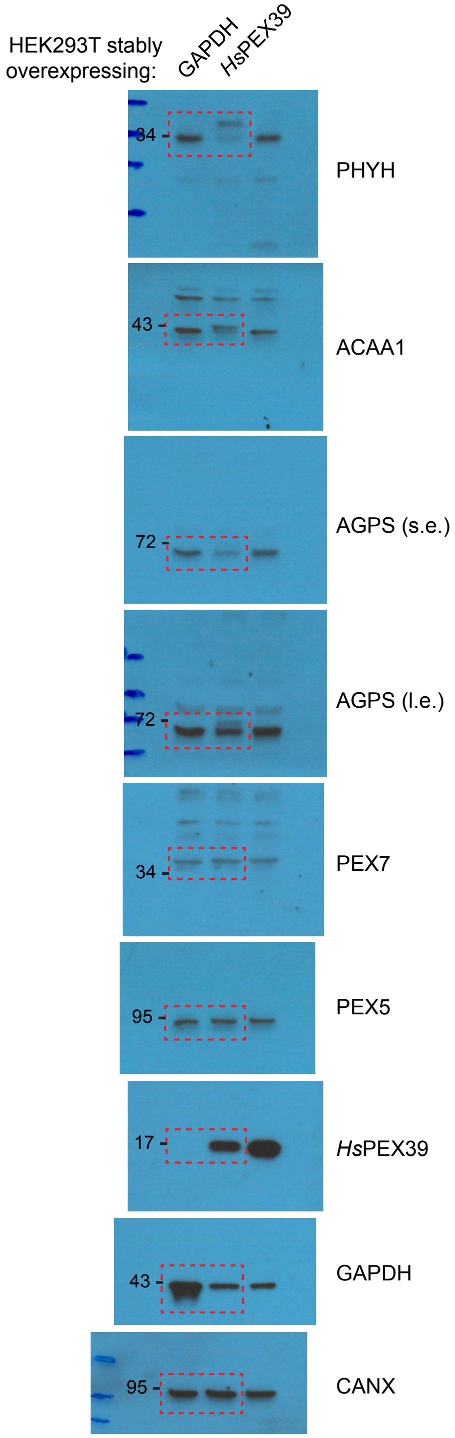

HEK293T stably overexpressing:
GAPDH
HsPEX39
34
PHYH
43
ACAA1
72
AGPS (s.e.)
72
AGPS (l.e.)
34
PEX7
95
PEX5
17
HsPEX39
43
GAPDH
95
CANX

Figure 3c

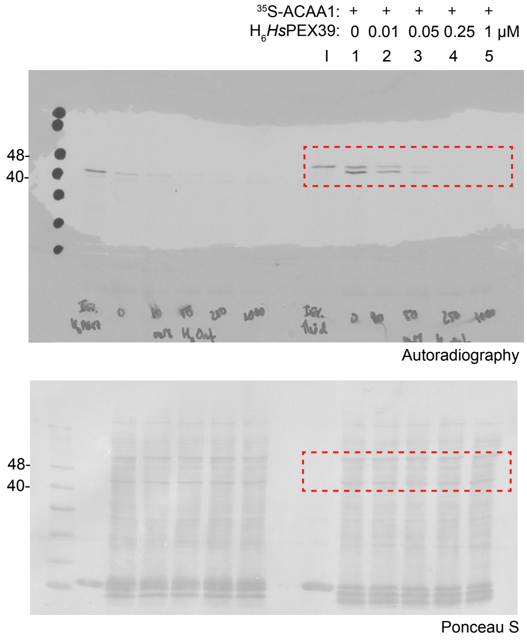

Figure 3d

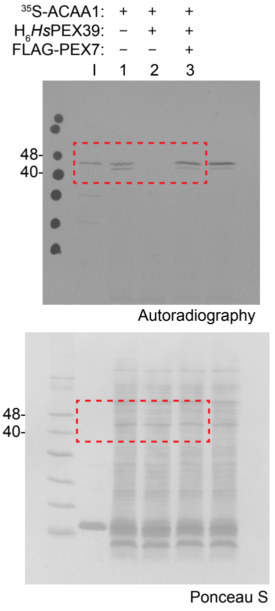

Figure 3f

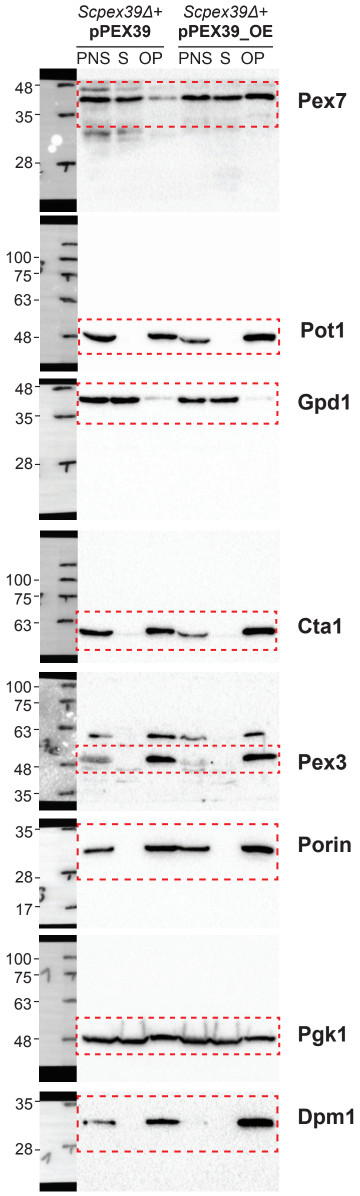
Scpex39Δ+ pPEX39
Scpex39Δ+ pPEX39_OE
PNS S OP PNS S OP
48
35
28
Pex7
100
75
63
48
Pot1
48
35
28
Gpd1
100
75
63
Cta1
100
75
63
48
35
Pex3
35
28
17
Porin
100
75
63
48
Pgk1
35
28
Dpm1

Figure 4a

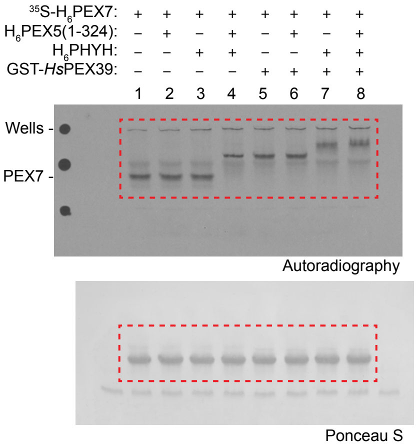

Figure 4b

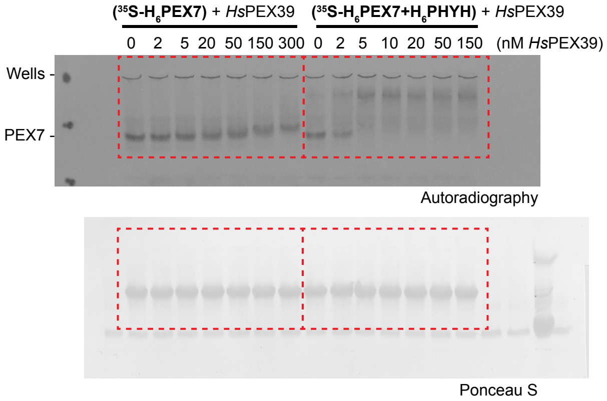

Figure 5a

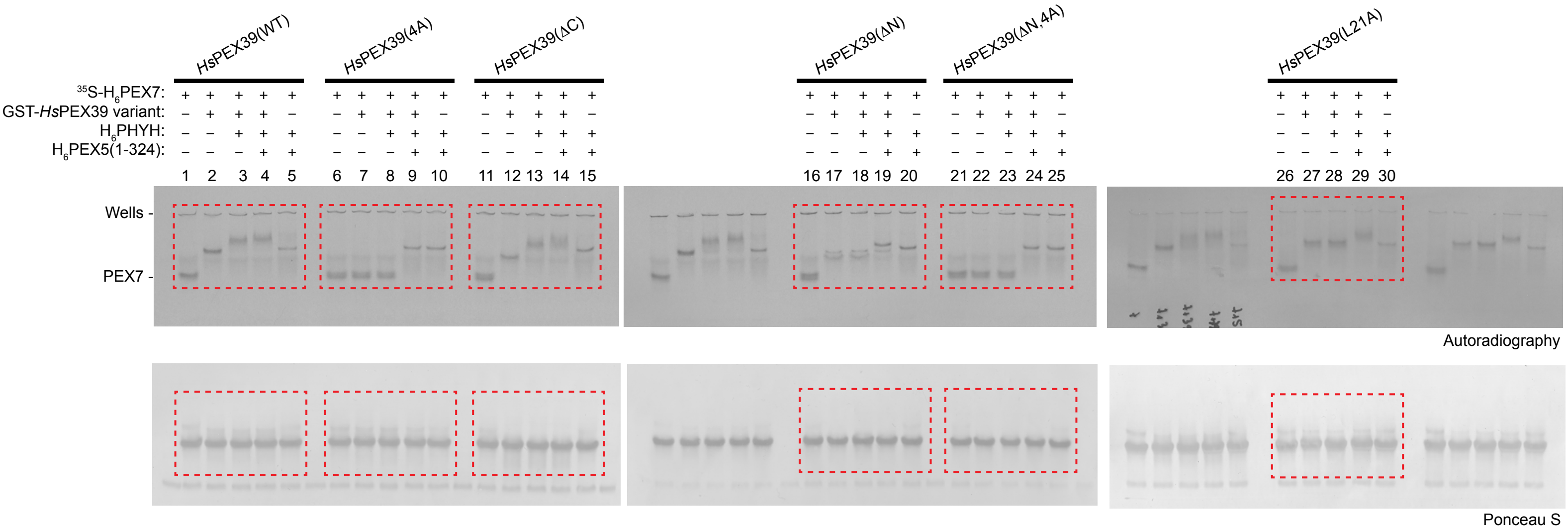

Figure 5b

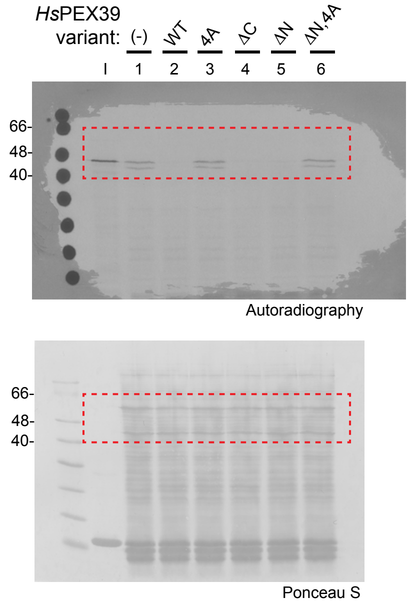

Figure 5c

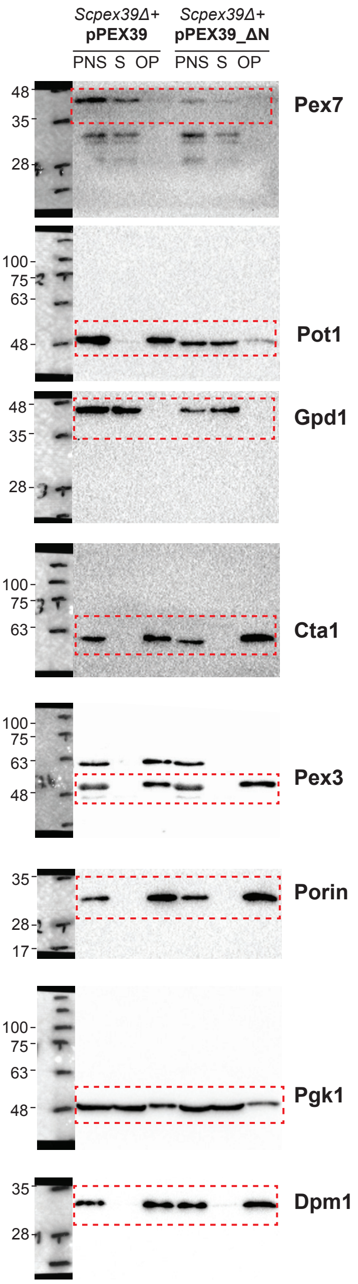

Figure 5d

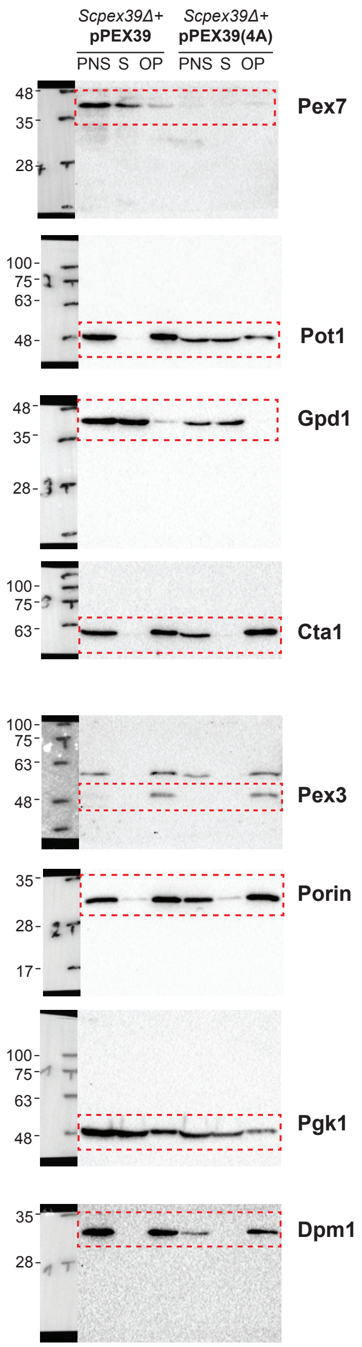

Figure 5f

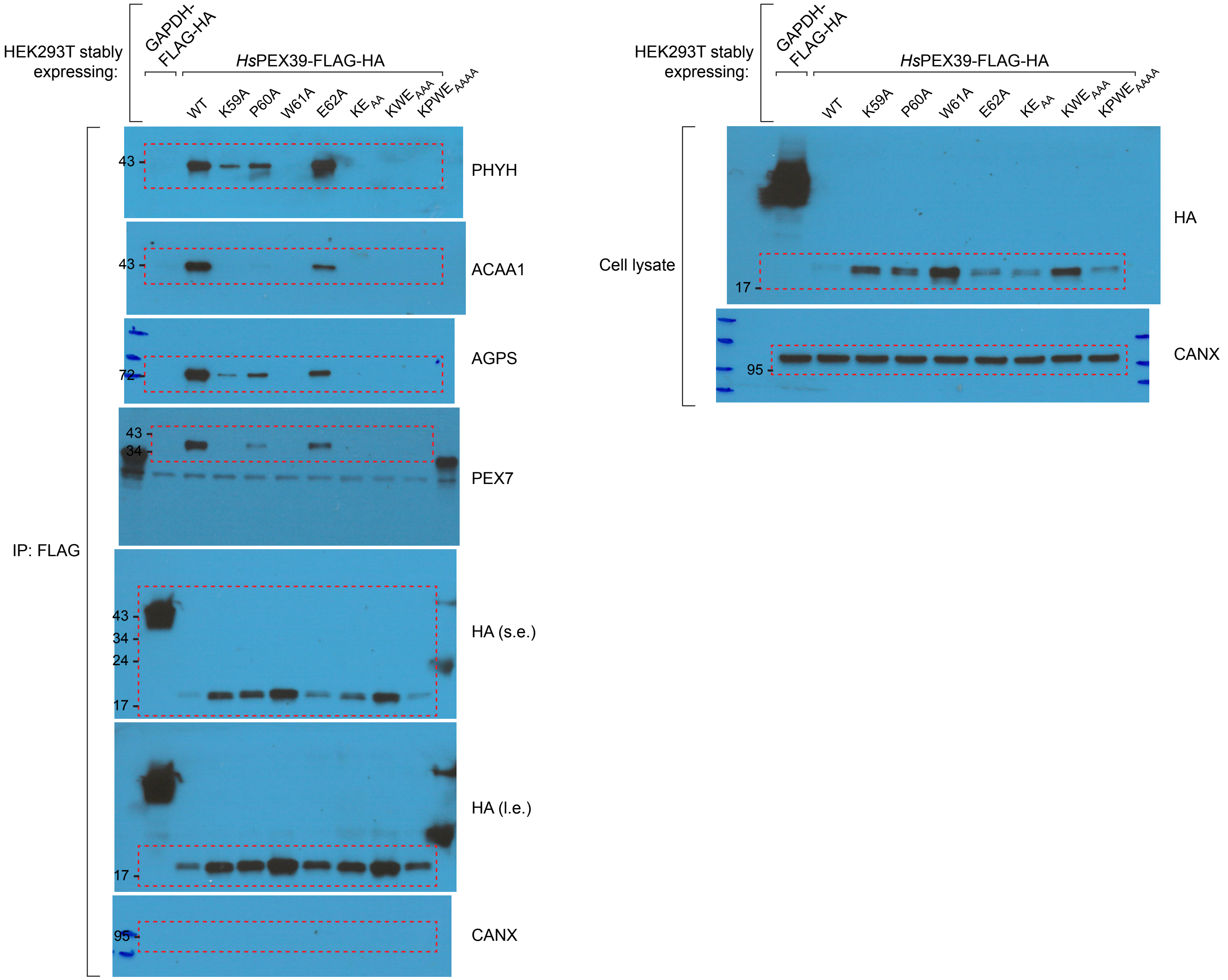

HEK293T stably expressing:
GAPDH-FLAG-HA
HsPEX39-FLAG-HA
WT
K59A
P60A
W61A
E62A
KEAA
KWEAAA
KPWEAAAA
IP: FLAG
PHYH
ACAA1
AGPS
PEX7
HA (s.e.)
HA (l.e.)
CANX
43
72
34
24
17
95
Cell lysate
HA
CANX

Figure 5h

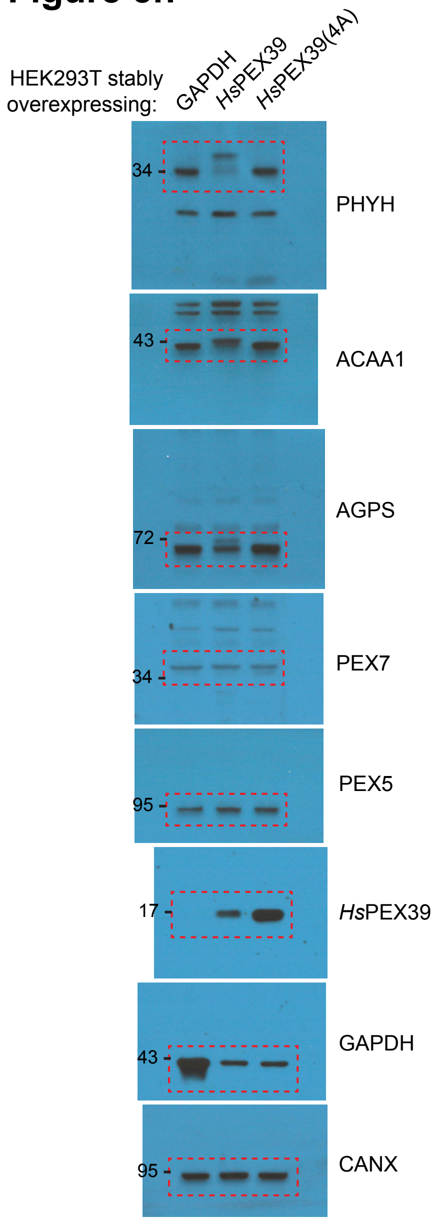

HEK293T stably overexpressing:
GAPDH
HsPEX39
HsPEX39(4A)
34
PHYH
43
ACAA1
AGPS
72
PEX7
34
PEX5
95
17
HsPEX39
GAPDH
43
CANX
95

Figure 6b

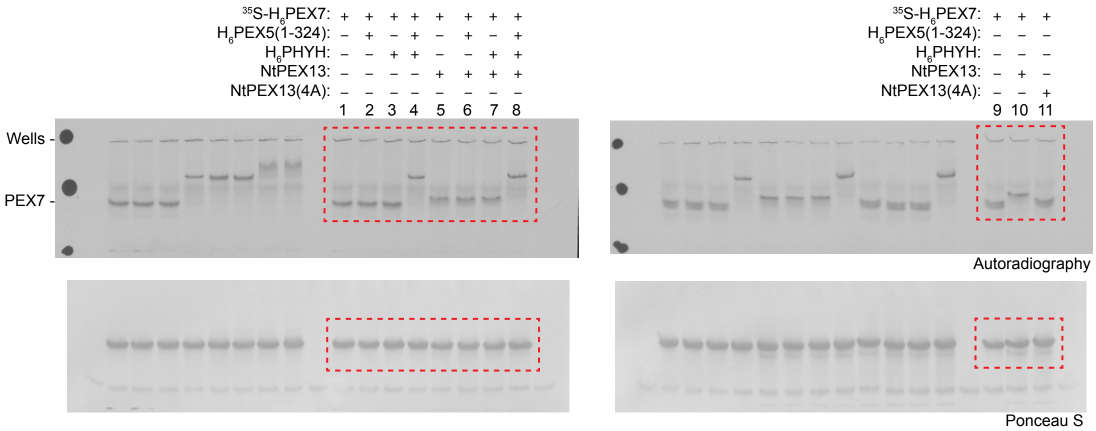

Figure 6c

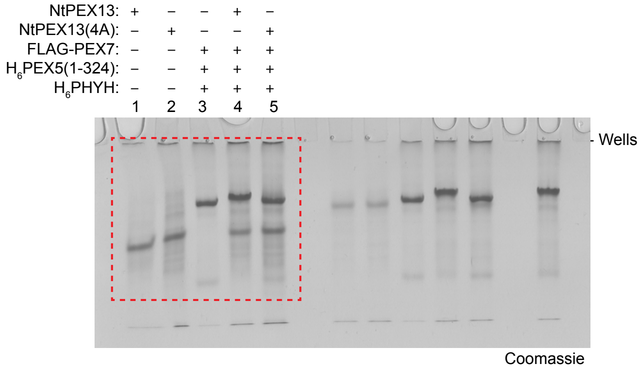

Figure 6d

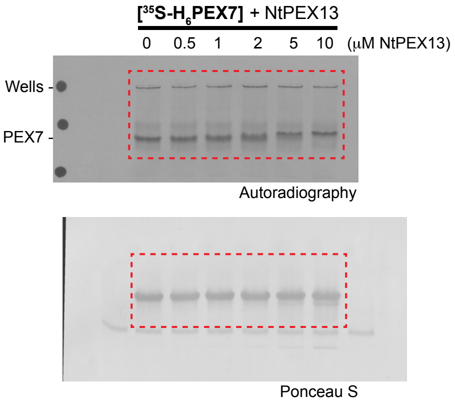

Figure 6g

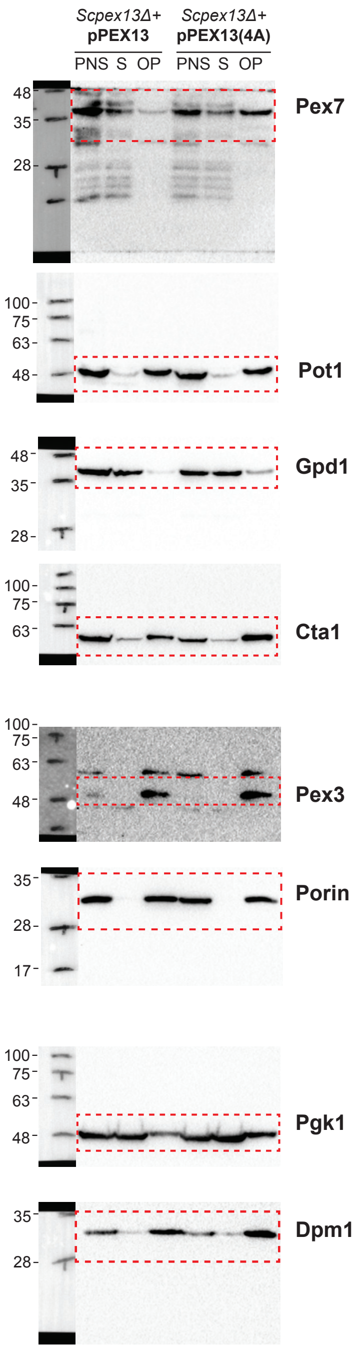

Scpex13Δ+ pPEX13
Scpex13Δ+ pPEX13(4A)
PNS S OP PNS S OP
48
35
28
Pex7
100
75
63
48
Pot1
48
35
28
Gpd1
100
75
63
Cta1
100
75
63
48
Pex3
35
28
17
Porin
100
75
63
48
Pgk1
35
28
Dpm1

Figure 6h

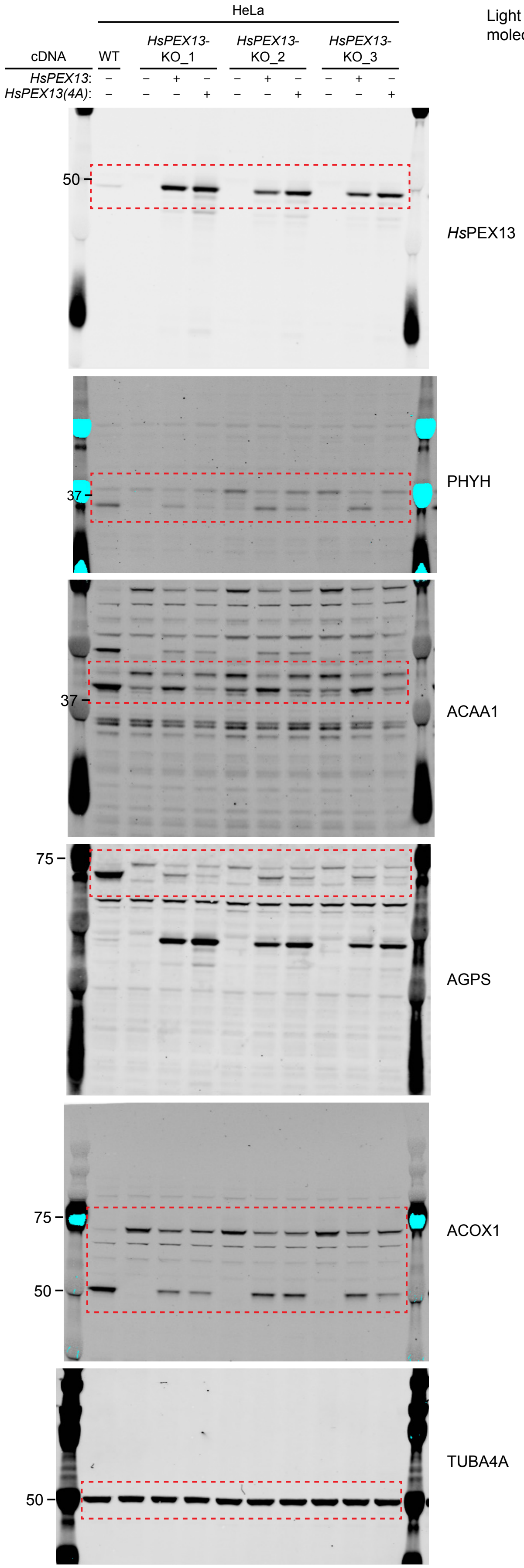

Light blue spots can be seen in some immunoblots because of detection of the prestained molecular weight markers by the Odyssey Infrared Imaging System depending on the exposure length.

Figure 7d

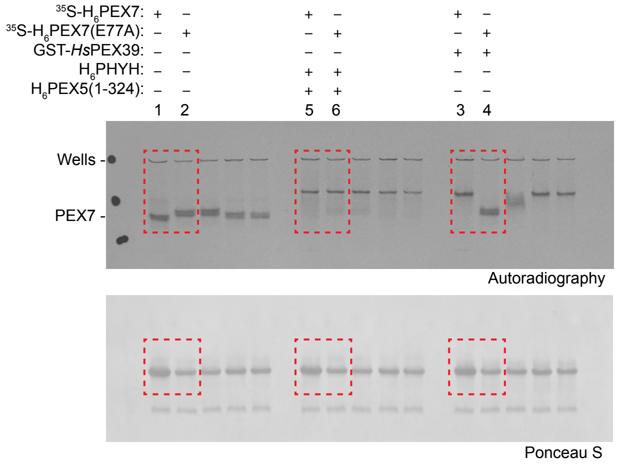

Figure 7e

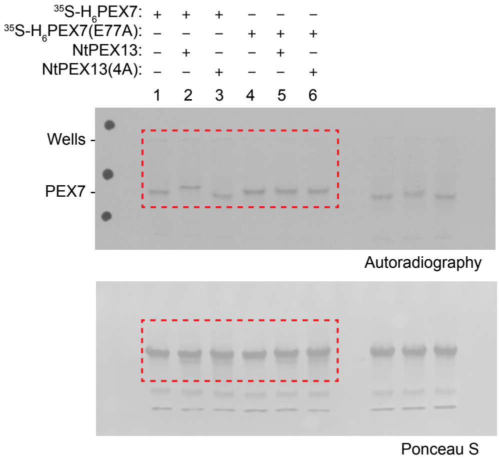

Figure 7f

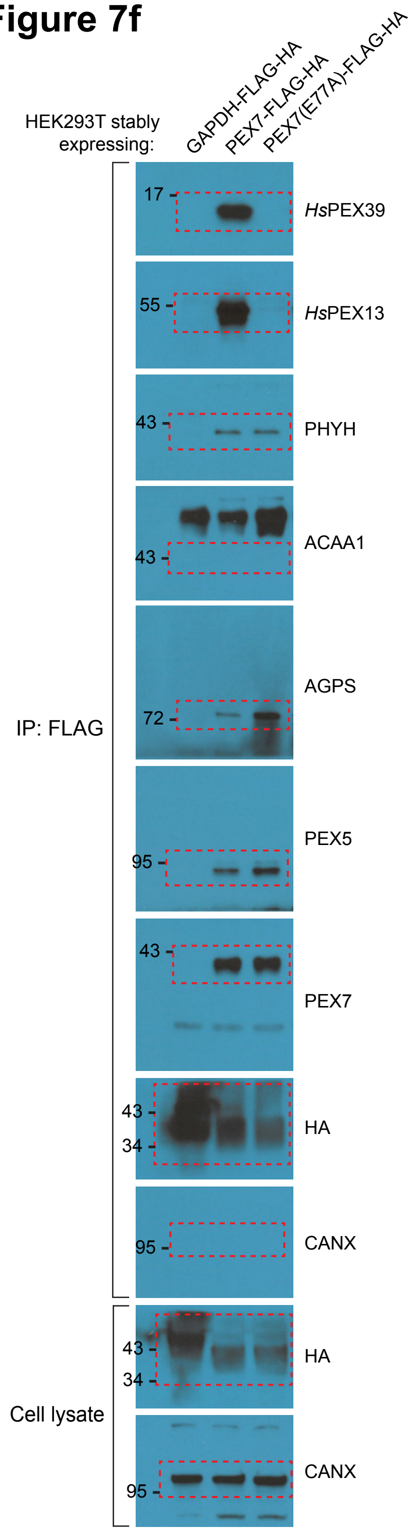
HEK293T stably expressing:
GAPDH-FLAG-HA
PEX7-FLAG-HA
PEX7(E77A)-FLAG-HA
IP: FLAG
17
HsPEX39
55
HsPEX13
43
PHYH
43
ACAA1
72
AGPS
95
PEX5
43
PEX7
43
34
HA
95
CANX
Cell lysate
43
34
HA
95
CANX

Figure 8a

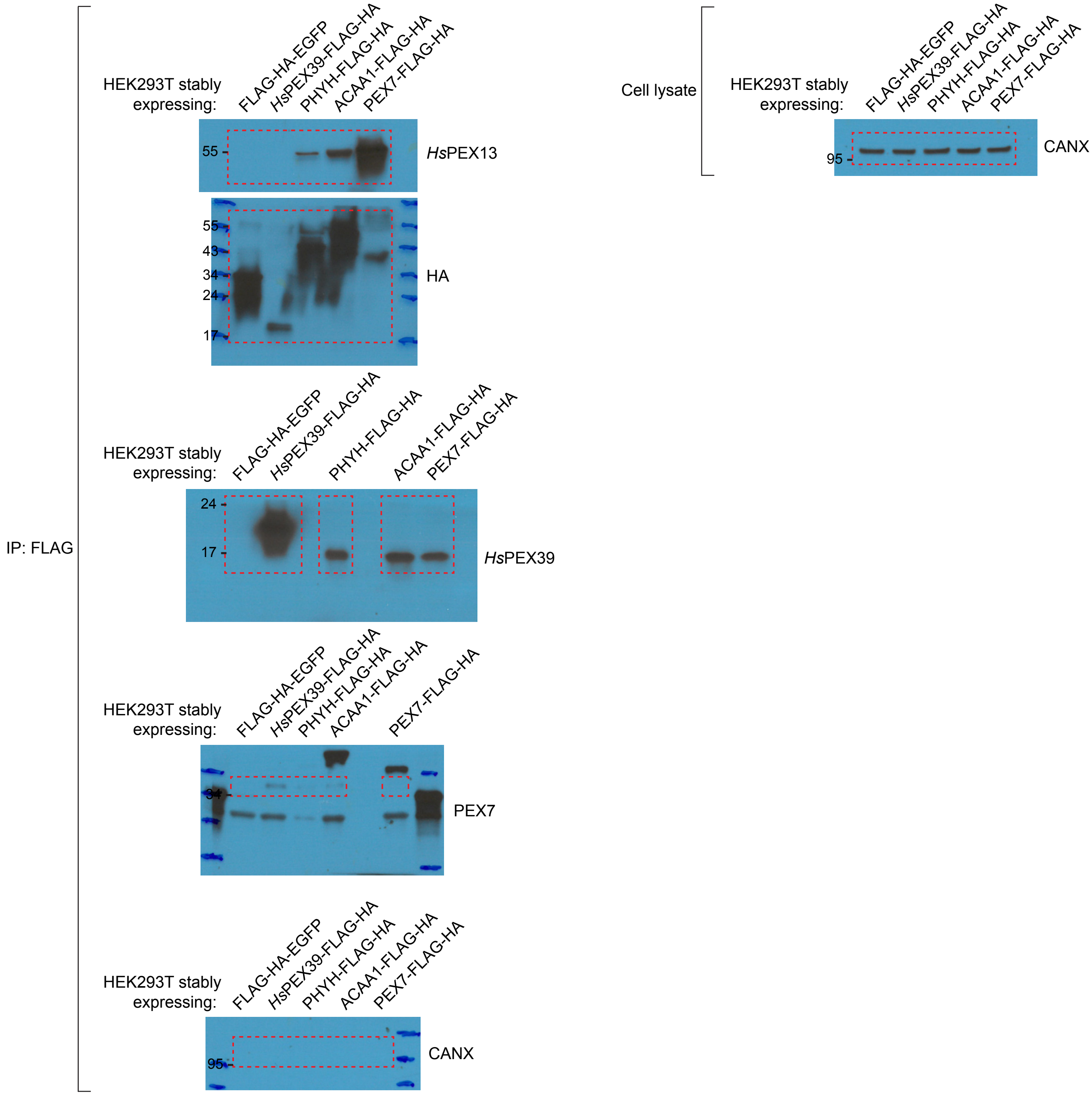

IP: FLAG
HEK293T stably expressing:
FLAG-HA-EGFP
HsPEX39-FLAG-HA
PHYH-FLAG-HA
ACAA1-FLAG-HA
PEX7-FLAG-HA
55
HsPEX13
55
43
34
24
17
HA
24
17
HsPEX39
34
PEX7
95
CANX
Cell lysate
95
CANX

Figure 8b

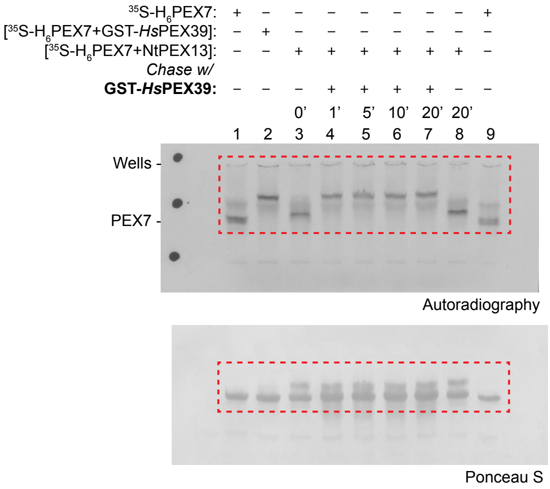

Figure 8c

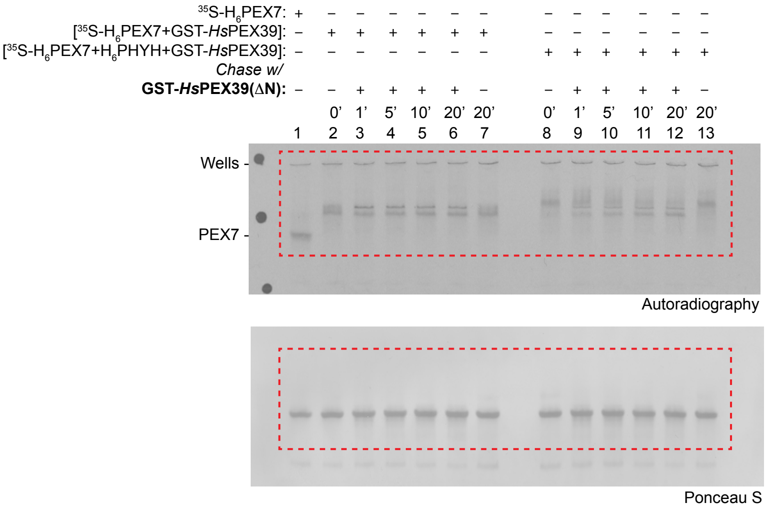

Ext. Data Figure 1b

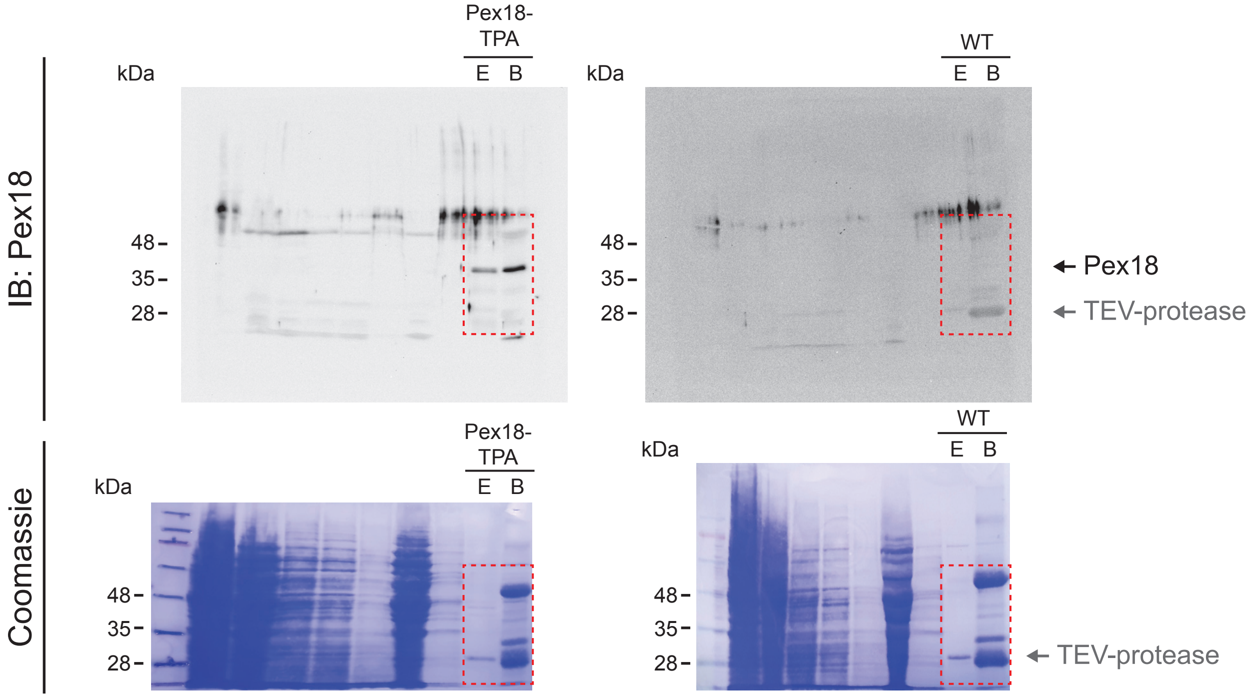

Ext. Data Figure 1f

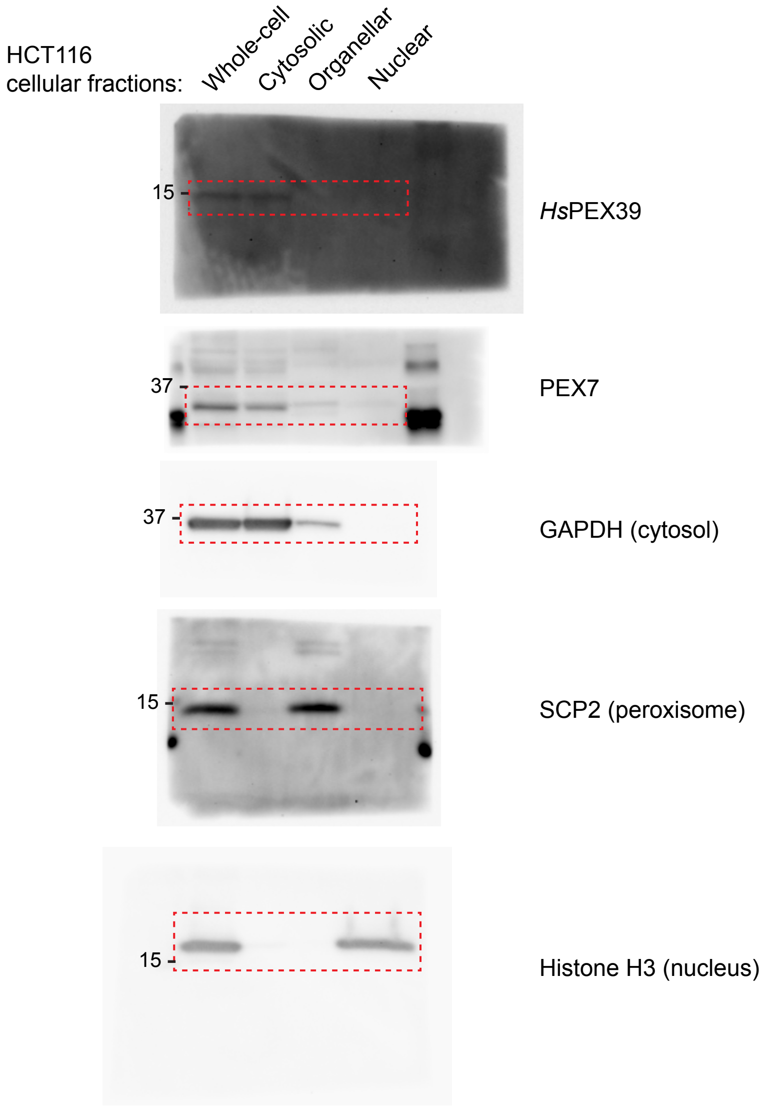

Ext. Data Figure 2a

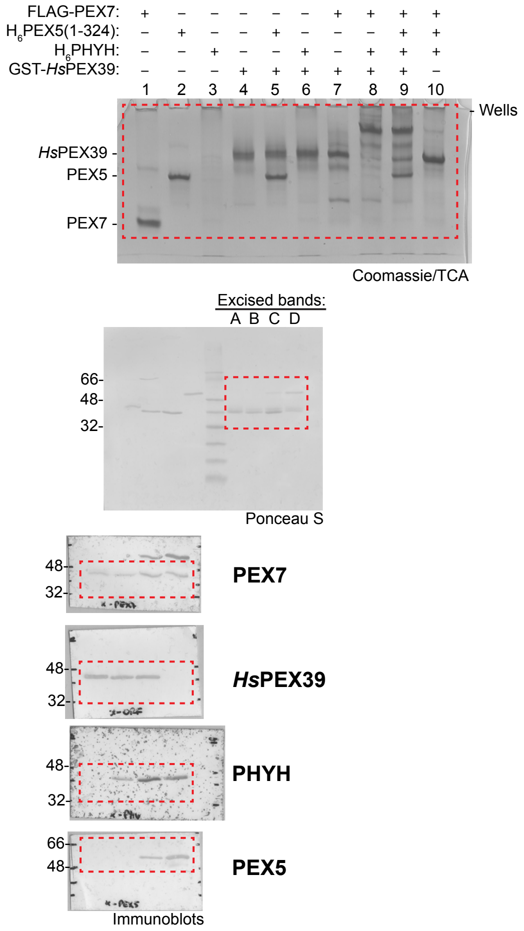

Ext. Data Figure 2b

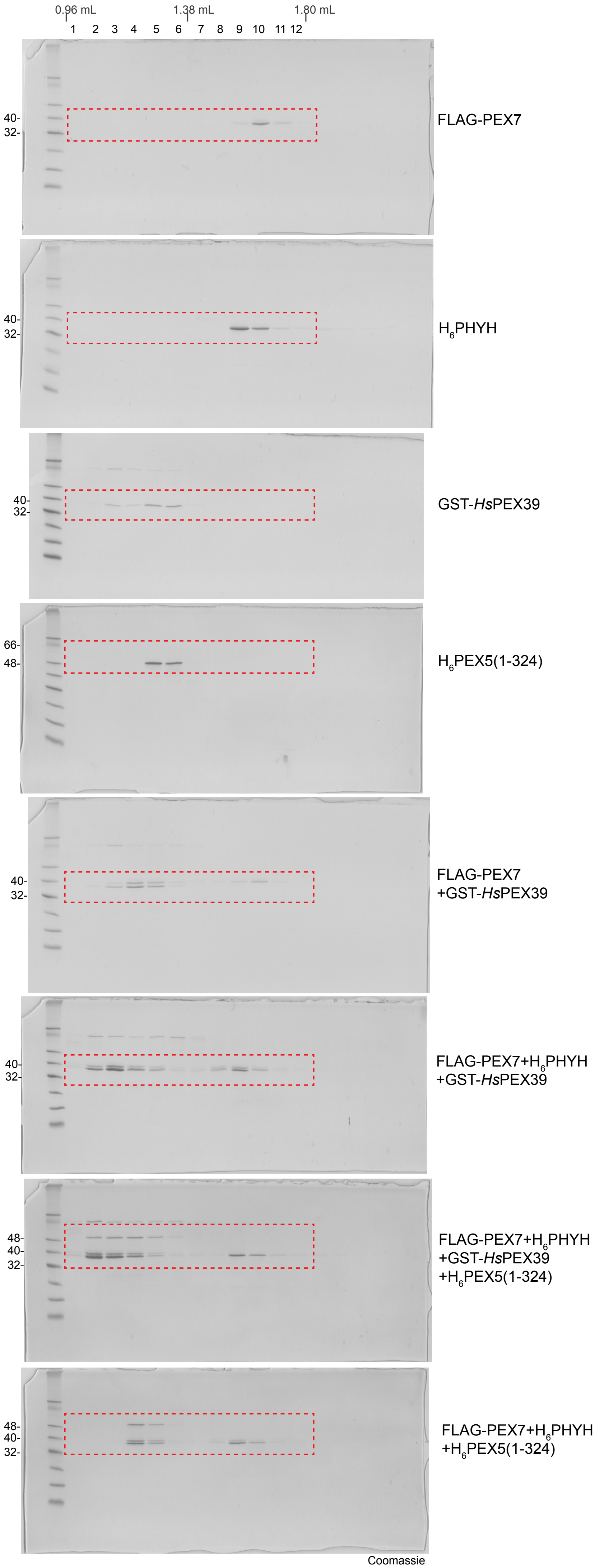

Ext. Data Figure 2c

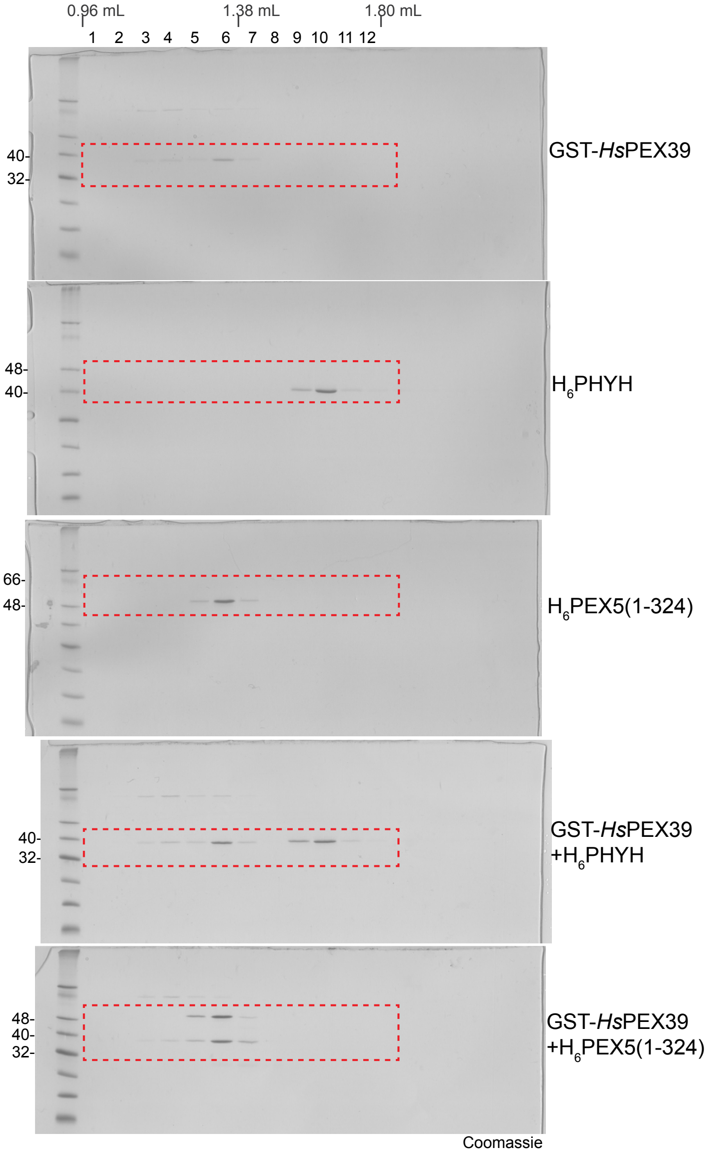

Ext. Data Figure 2d

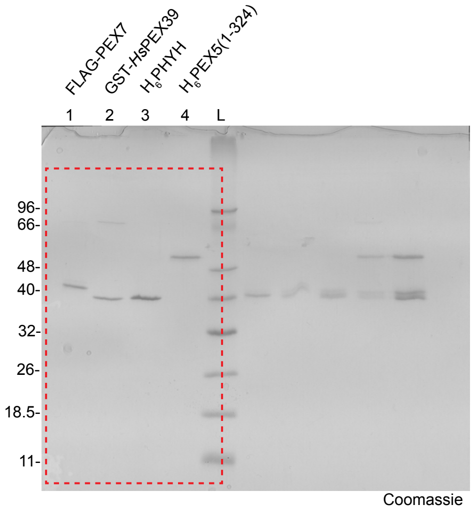

Ext. Data Figure 3c

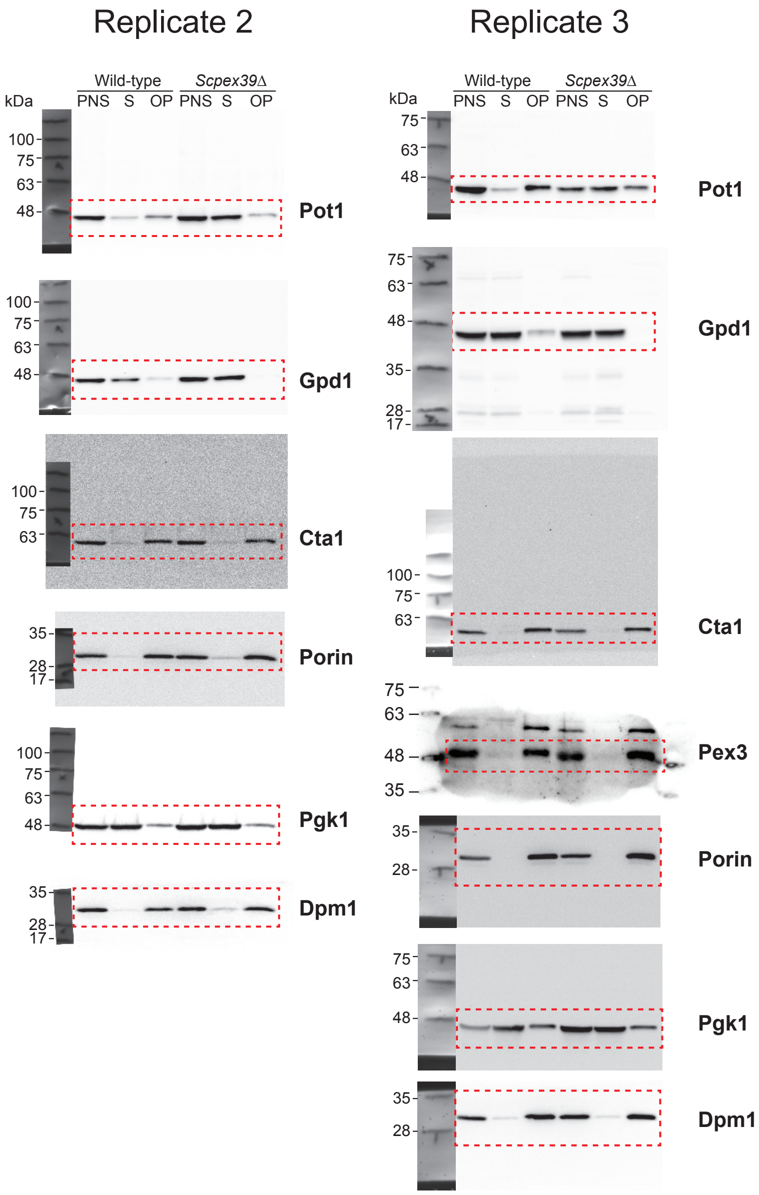

Ext. Data Figure 3d

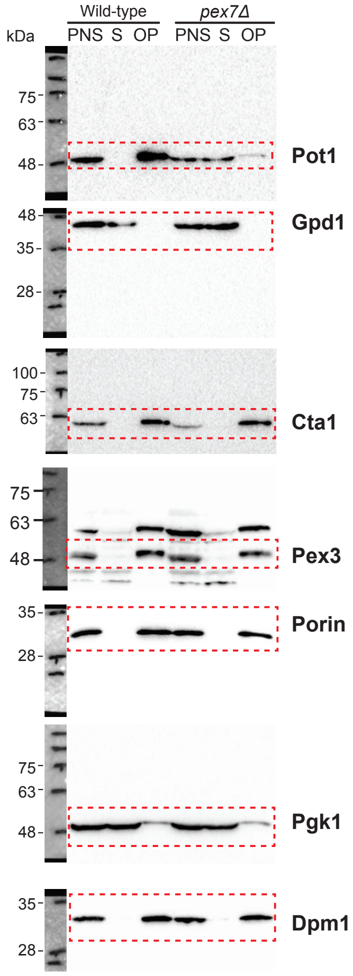

Ext. Data Figure 3e

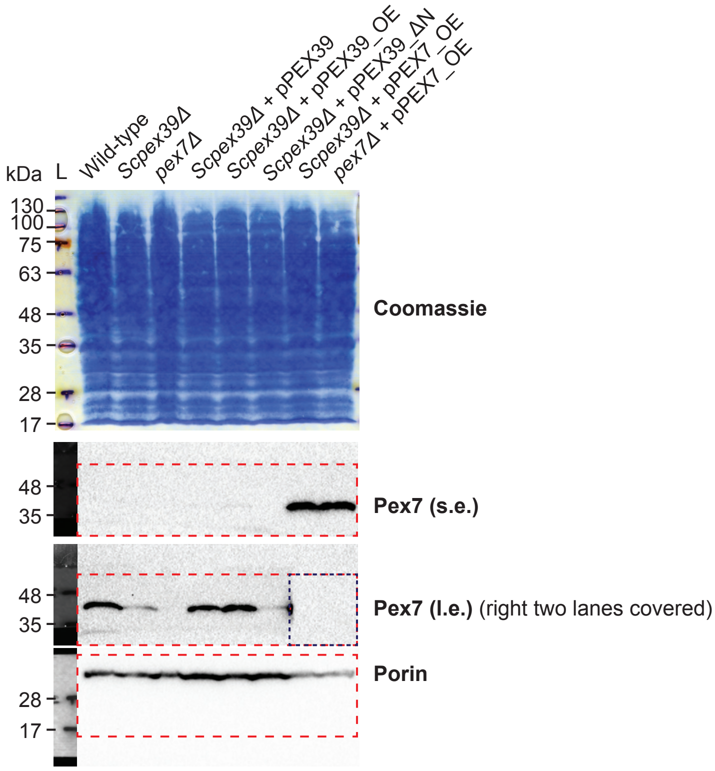

Ext. Data Figure 3f (upper)

Ext. Data Figure 3f (lower)

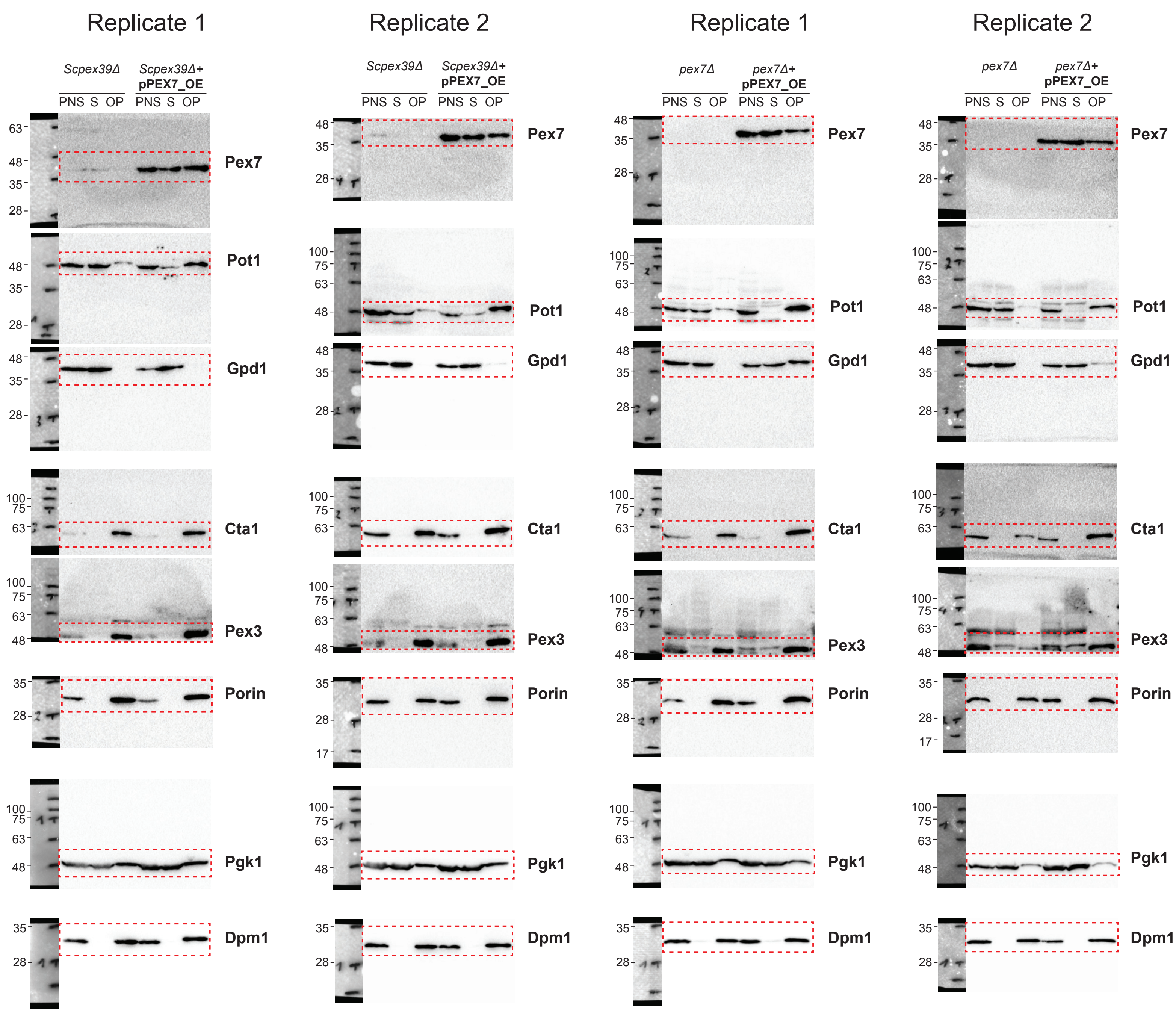

Ext. Data Figure 4b

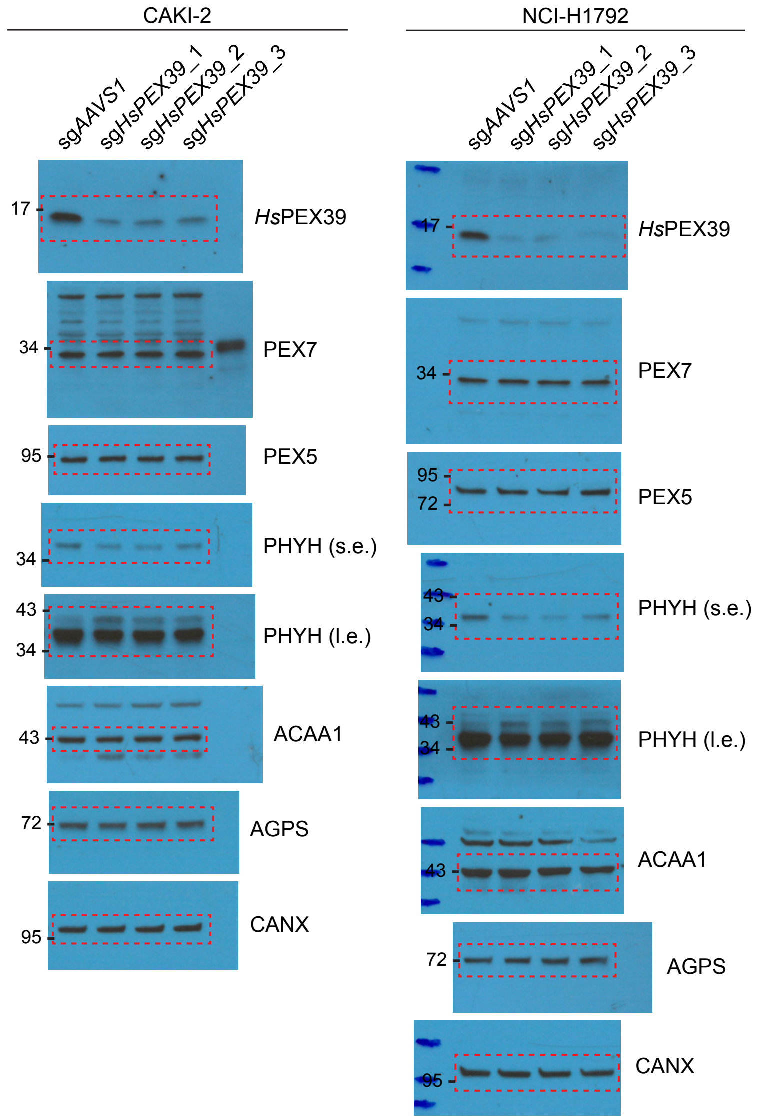

Ext. Data Figure 4c

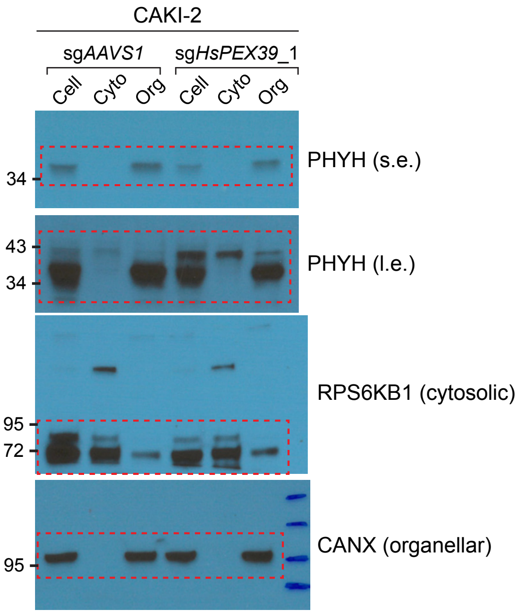

Ext. Data Figure 5a

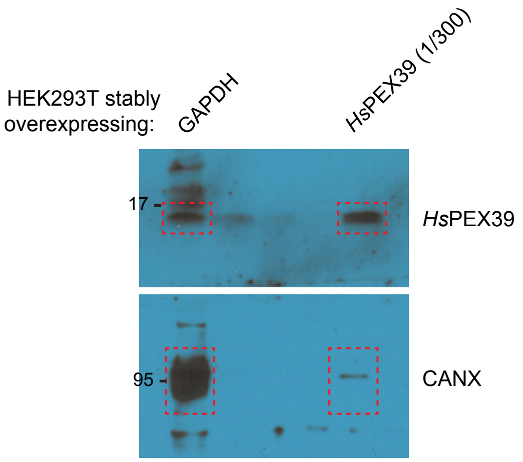

Ext. Data Figure 5b

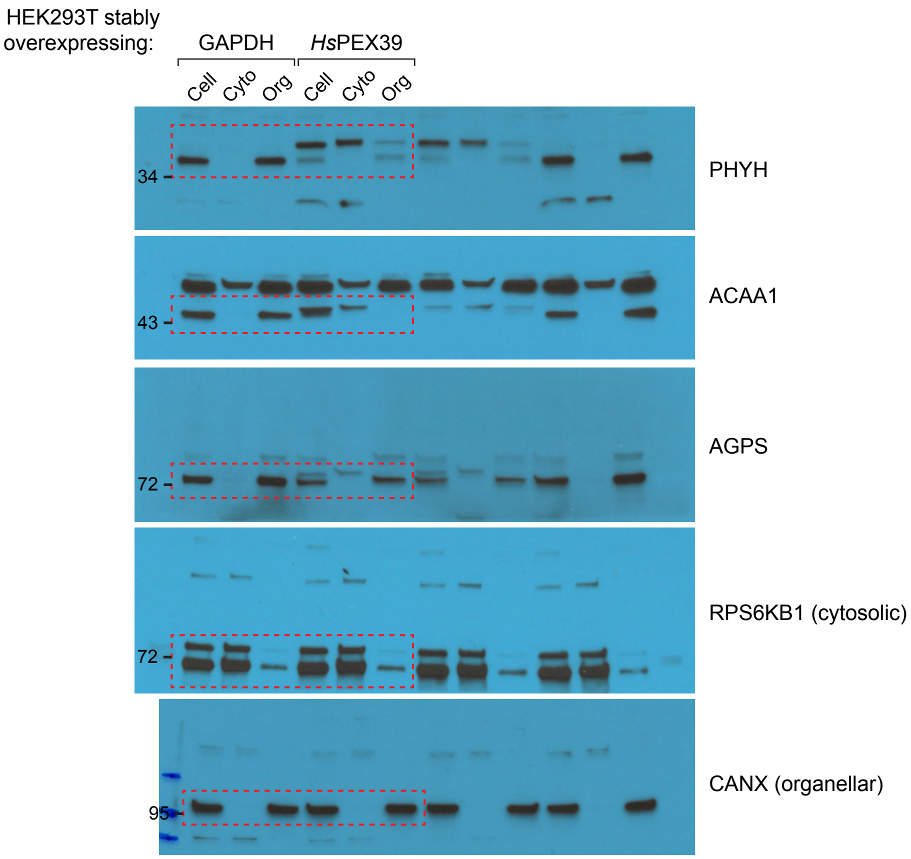

Ext. Data Figure 5d

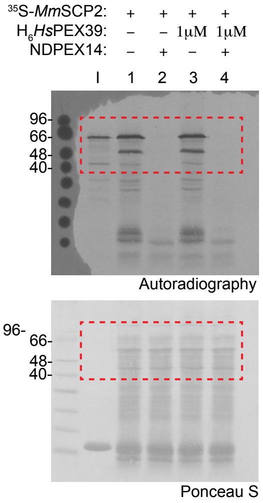

Ext. Data Figure 5e

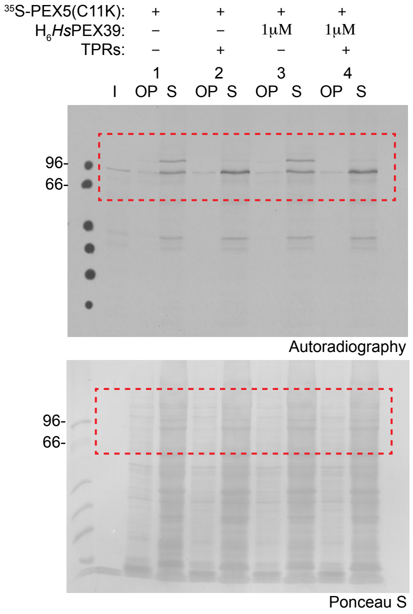

Ext. Data Figure 5h

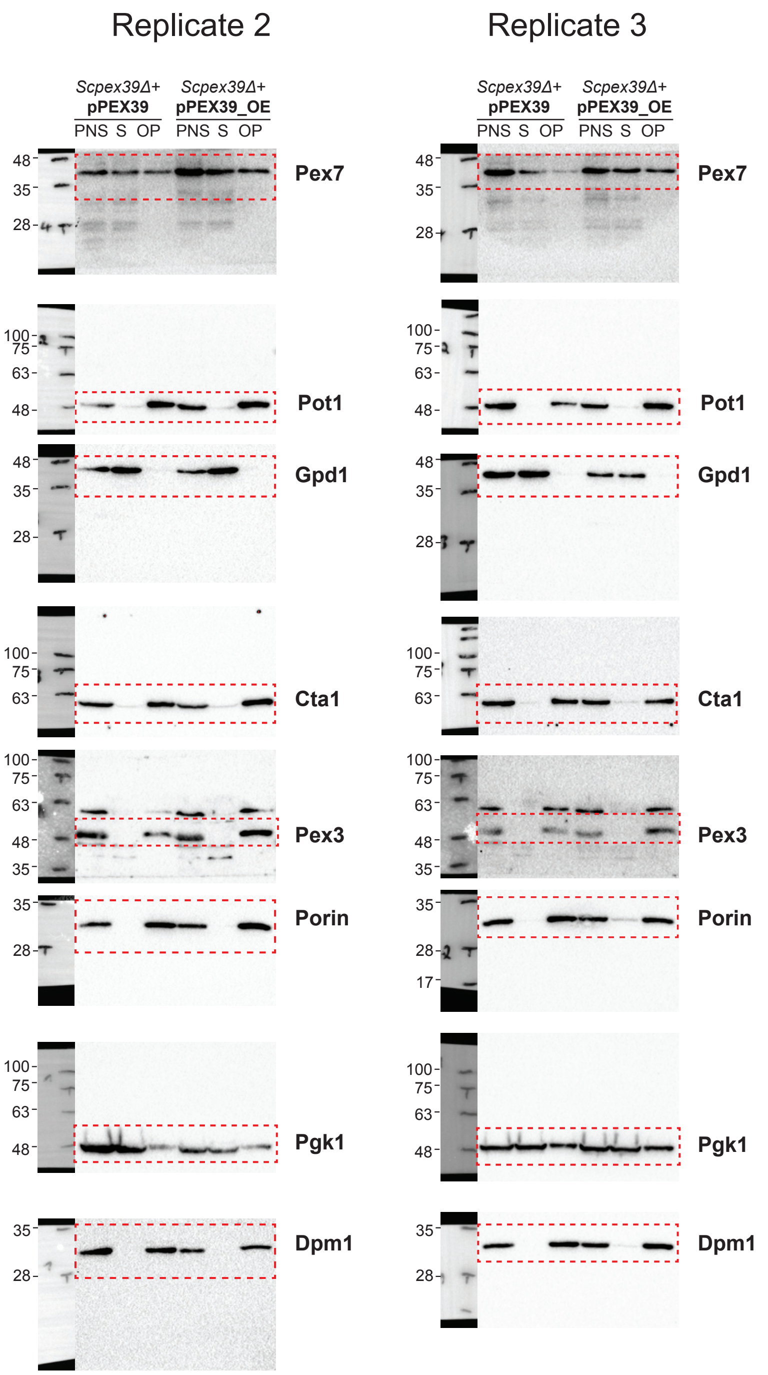

Ext. Data Figure 5i

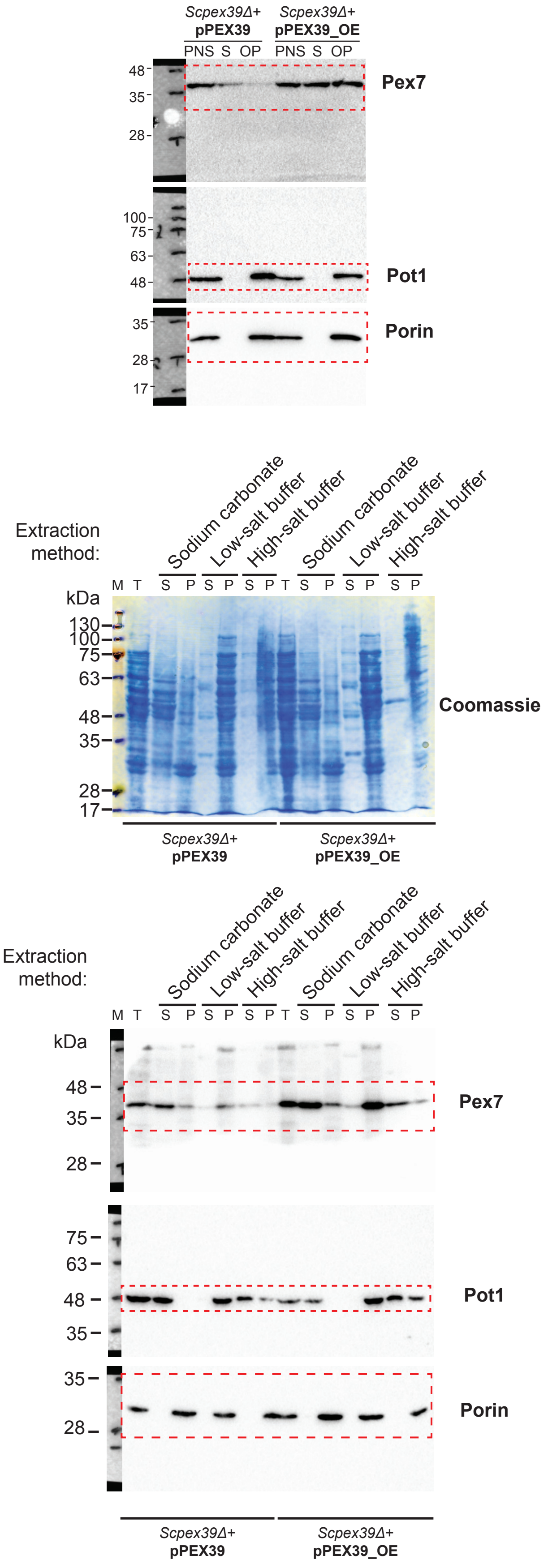

Ext. Data Figure 6a

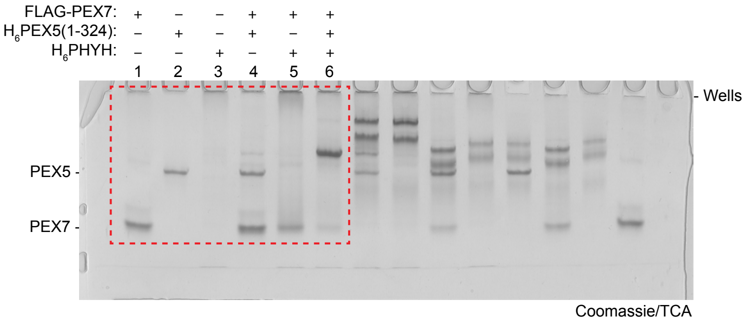

Ext. Data Figure 7a

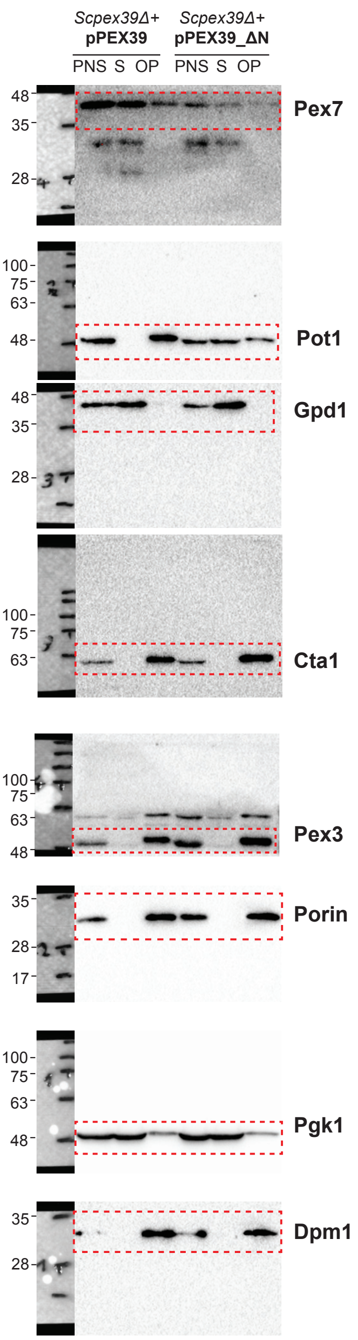

Ext. Data Figure 7b

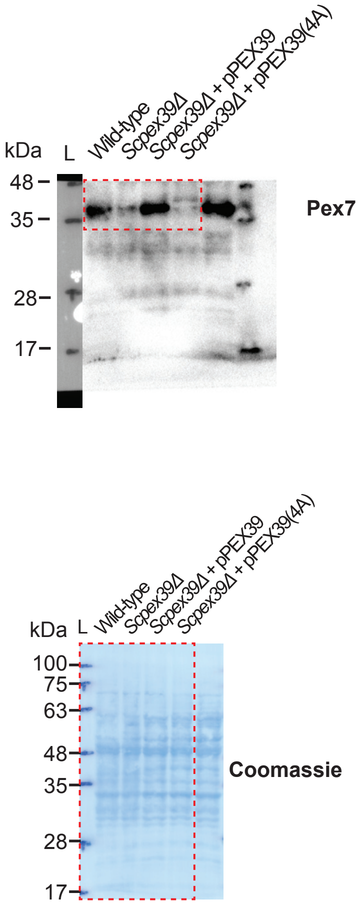

Ext. Data Figure 7c

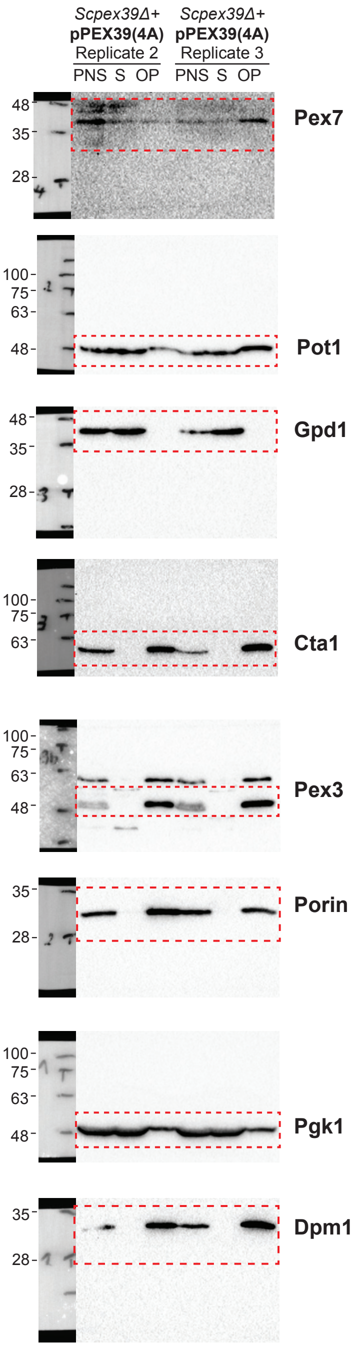

Ext. Data Figure 7g

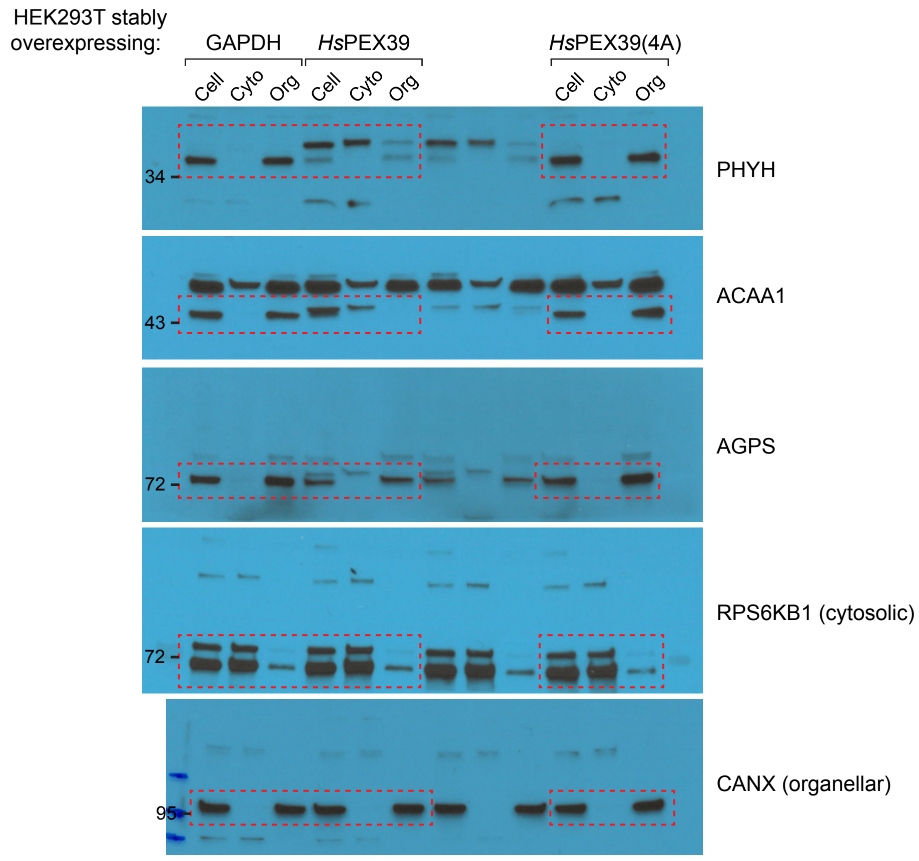

The data for cells overexpressing GAPDH and wild-type *Hs*PEX39 are the same as shown previously in Extended Data Fig. 5b and are used here as comparisons for overexpression of *Hs*PEX39 with mutation of the KPWE motif to AAAA [*Hs*PEX39(4A)]. The 3 overexpression lines were processed together in the same experiment to generate these data.

Ext. Data Figure 8b

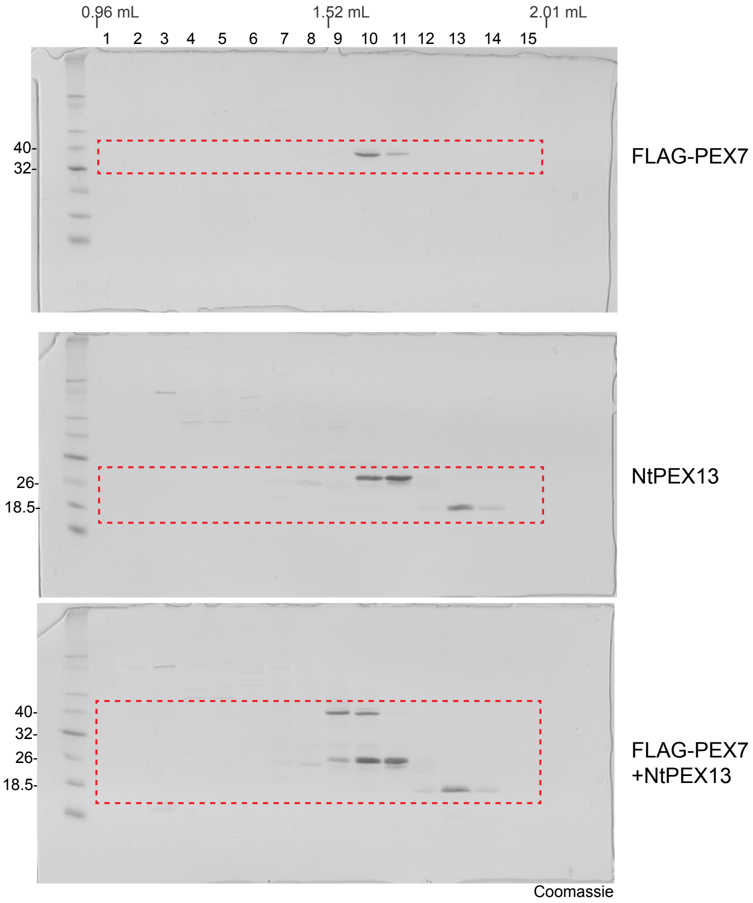

Ext. Data Figure 8d

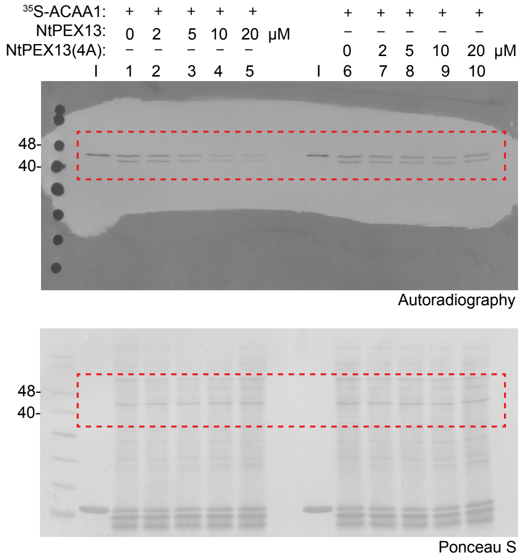

Ext. Data Figure 8e

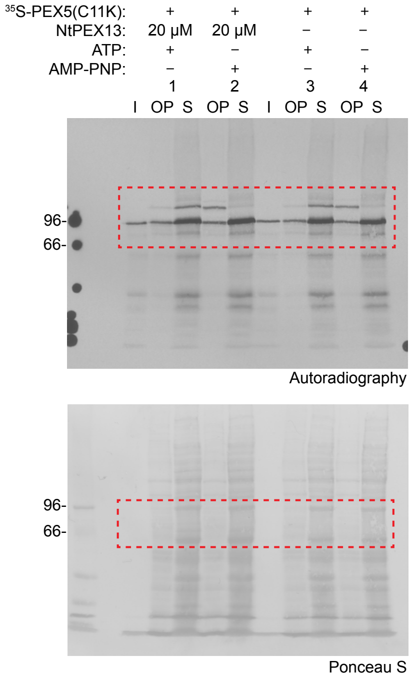

Ext. Data Figure 9a

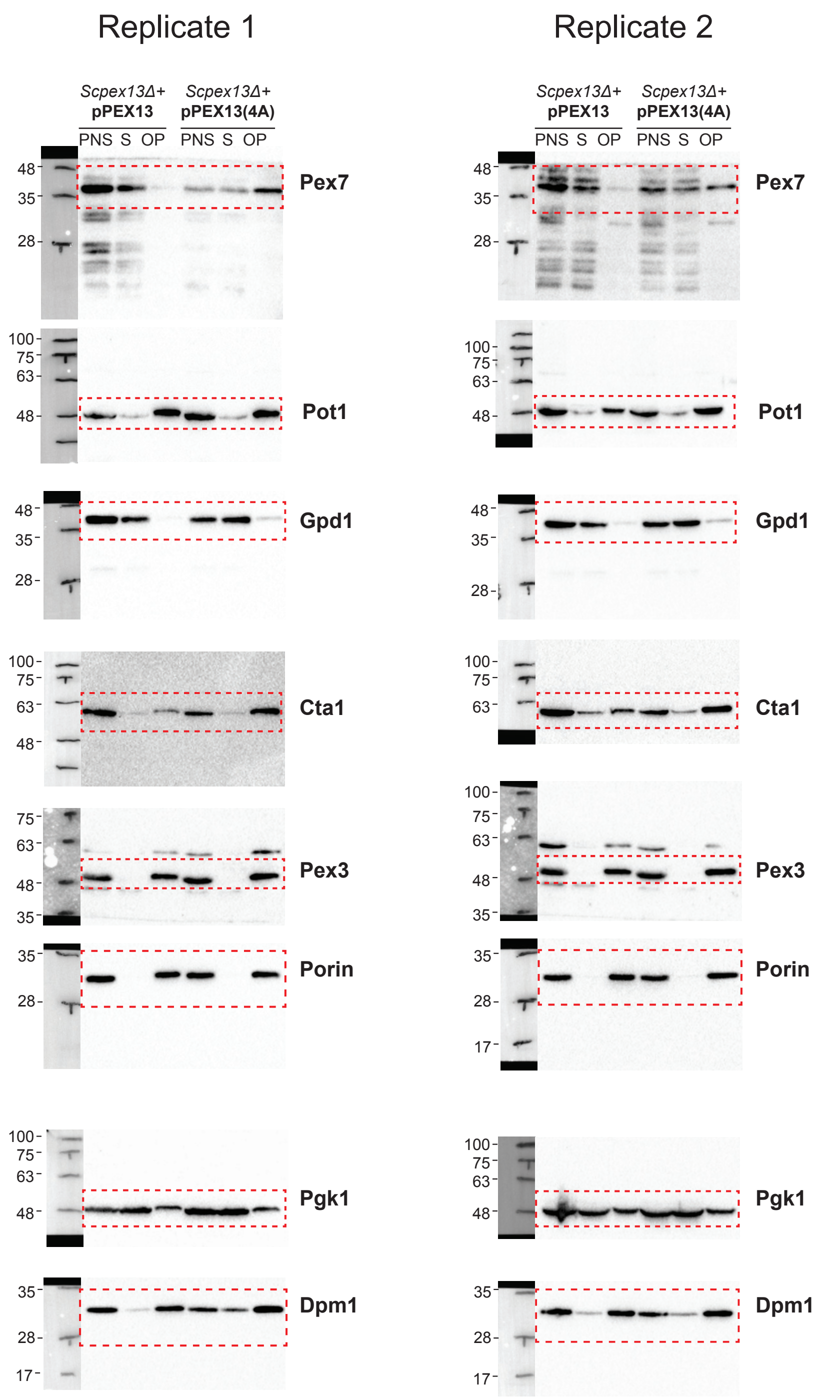

Ext. Data Figure 9b

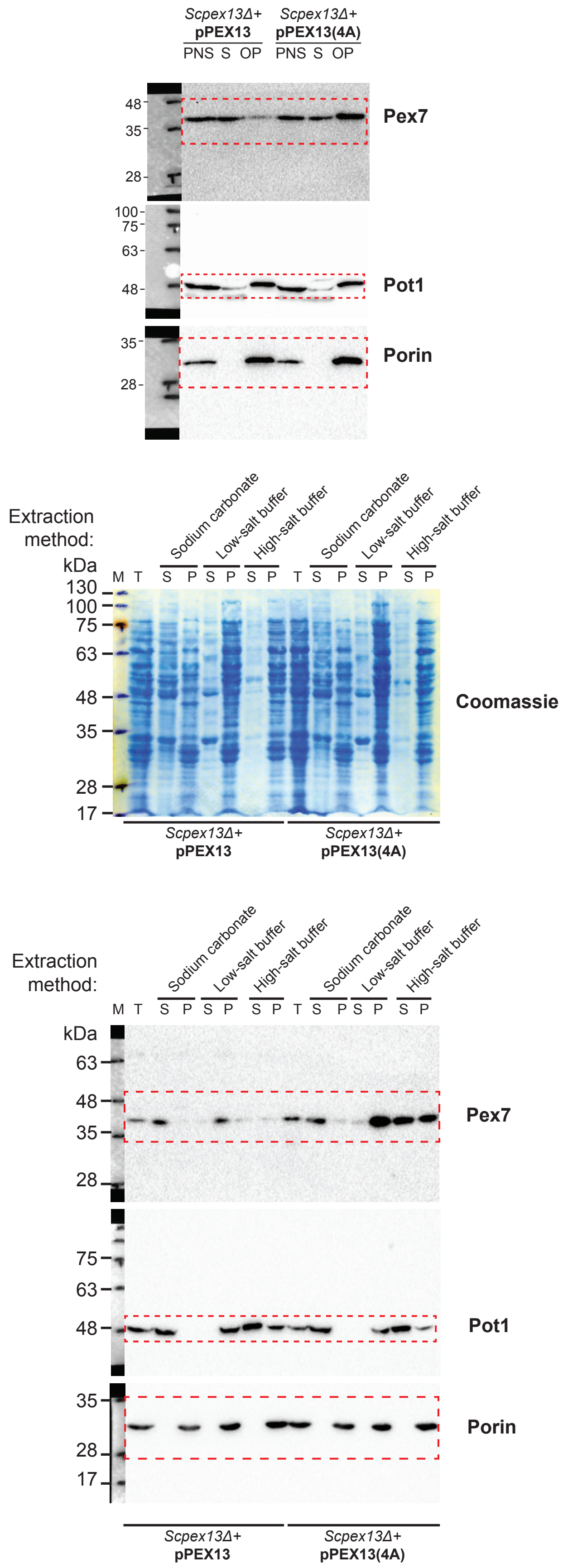

Supplement: Supplementary file 3 — Full-length, unprocessed gels and blots. [file 41556_2025_1711_MOESM3_ESM.pdf]
